# Riluzole for treating spasticity in patients with chronic traumatic spinal cord injury: Study protocol in the phase ib/iib adaptive multicenter randomized controlled RILUSCI trial

**DOI:** 10.1371/journal.pone.0276892

**Published:** 2023-01-20

**Authors:** Maëva Cotinat, Isabelle Boquet, Moreno Ursino, Cécile Brocard, Elisabeth Jouve, Corinne Alberti, Laurent Bensoussan, Jean-Michel Viton, Frédéric Brocard, Olivier Blin

**Affiliations:** 1 Institut de Neurosciences de la Timone (UMR7289), Aix-Marseille Université and CNRS, Marseille, France; 2 Department of Physical and Rehabilitation Medicine, Sainte Marguerite University Hospital, APHM, Marseille, France; 3 Unit of Clinical Epidemiology, Assistance Publique-Hôpitaux de Paris, Centre Hospitalier Universitaire Robert Debré, FCRIN PARTNERS Platform, Université de Paris, Sorbonne Paris-Cité, INSERM U1123 and CIC-EC 1426, Paris, France; 4 Centre de Recherche des Cordeliers, INSERM, Sorbonne Université, USPC, Université de Paris, F-75006 Paris, France; 5 Inria, Paris, France; 6 Aix Marseille University, APHM, INSERM, Inst Neurosci Syst, UMR1106, Service de Pharmacologie Clinique et Pharmacovigilance, Marseille, France; 7 Institut Universitaire de Réadaptation de Valmante Sud, UGECAM, Marseille, France; University of Toronto, CANADA

## Abstract

**Background:**

Satisfactory treatment is often lacking for spasticity, a highly prevalent motor disorder in patients with spinal cord injury (SCI). Low concentrations of riluzole potently reduce the persistent sodium current, the post-SCI increase in which contributes to spasticity. The repurposing of this drug may therefore constitute a useful potential therapeutic option for relieving SCI patients suffering from chronic traumatic spasticity.

**Objective:**

RILUSCI is a phase 1b–2b trial designed to assess whether riluzole is a safe and biologically effective means of managing spasticity in adult patients with traumatic chronic SCI.

**Methods:**

In this multicenter double-blind trial, adults (aged 18–65 years) suffering from spasticity after SCI (target enrollment: 90 participants) will be randomly assigned to be given either a placebo or a recommended daily oral dose of riluzole for two weeks. The latter dose will be previously determined in phase 1b of the study by performing double-blind dose-finding tests using a Bayesian continuous reassessment method. The primary endpoint of the trial will be an improvement in the Modified Ashworth Score (MAS) or the Numerical Rating Score (NRS) quantifying spasticity. The secondary outcomes will be based on the safety and pharmacokinetics of riluzole as well as its impact on muscle spasms, pain, bladder dysfunction and quality of life. Analyses will be performed before, during and after the treatment and the placebo-controlled period.

**Conclusion:**

To the best of our knowledge, this clinical trial will be the first to document the safety and efficacy of riluzole as a means of reducing spasticity in patients with chronic SCI.

**Trial registration:**

The clinical trial, which is already in progress, was registered on the ClinicalTrials.gov website on August 9, 2016 under the registration number NCT02859792.

**Trial sponsor:**

Assistance Publique–Hôpitaux de Marseille.

## Introduction

Spasticity, one of the most disabling motor impairments, has several pathological causes [such as spinal cord injury (SCI), stroke, multiple sclerosis, cerebral palsy, etc.] [1–3] and affects more than 12 million people worldwide. Spasticity has been mainly defined as heightened muscle tone (hypertonia) due to a velocity-dependent increase in the tonic stretch reflexes [4]. Spasticity is a highly prevalent medical condition occurring after SCI, in particular. It develops in approximately 75% of patients up to one year after SCI [5, 6]. Spasticity can impair patients’ ability to perform activities of daily living by decreasing their mobility, and can also result in symptoms such as pain, sleep disturbances and depression. While antispastic medications can be helpful, no single medication has a beneficial effect in all individuals and each medication has potentially serious side effects [7–10]. When this treatment no longer suffices or is poorly tolerated, no other effective means of treating generalized spasticity are available. There therefore exists a clinical need to improve the management of spasticity.

### Overview of the clinical management of spasticity after SCI

When spasticity becomes troublesome, the therapeutic options available include various pharmacological and non-pharmacological approaches [7, 11, 12]. It is generally agreed that physical rehabilitation is an essential component in the management of spasticity as the first line of defense [13]. As an adjunct to physical rehabilitation, peripheral chemical denervation induced by the intramuscular injection of botulinum toxin (BoNT/A) has been found to relieve focal spasticity effectively, but the effects wear off within about 3 months [14]. Systemic pharmacological drugs for treating spasticity are often prescribed to SCI patients [10]. Baclofen is often the first-line medication used to treat spasticity, for example, but it can be necessary to switch to other medications because of the side effects, the commonest of which are drowsiness (7–70%), excessive weakness (1–69%), somnolence (4.5–70%), vertigo (4.5–13%) and psychological disturbance (1.7–19%) [15, 16]. The rate of treatment discontinuation due to intolerable adverse effects has been reported between 4 to 27%. The main risks of oral baclofen administration are related to withdrawal: seizures, psychic symptoms and hyperthermia can occur [16]. In some cases, an intrathecal delivery can be envisaged. This is obviously an invasive and expensive treatment which requires careful selection of the patients. It is worth noting in particular that withdrawal of baclofen can lead to a severe rebound of spasticity. It is therefore important to take into account the risks associated with the medication prescribed, depending on the patients’ comorbidities. In view of these problems, efforts to expand the range of antispastic drugs available are now urgently required in order to be able to dispense more closely tailored medical solutions, depending on individual patients’ condition. For this purpose, we have adopted Sir James Black’s famous strategy, as stated in 1988: “*The most fruitful basis for the discovery of a new drug is to start with an old drug*”. In line with this statement, we must be able to identify new therapeutic opportunities for treating spasticity based on previously existing approved drugs.

### Pathophysiology of spasticity and the reasons for repurposing riluzole as an antispastic drug

Maladaptive mechanisms promoting a generalized spinal hyperexcitability take place after SCI, and typical signs of spasticity then appear [17–19]. Impaired electrophysiological properties of sublesional neurons are closely involved in the emergence of spasticity [20]. In particular, a switch in the excitability of neurons innervating muscles (motoneurons) from the hypoexcitable state during spinal shock to the hyperexcitable state often occurs several weeks to months after SCI [19–23]. This switch induces motoneurons to express a self-sustained spiking activity triggered by a prolonged state of depolarization known as a *“plateau potential”* [24, 25]. Therefore, plateau potentials constitute a sustained excitatory drive that causes motoneurons to fire beyond the offset of the relevant stimuli, and ultimately generate the exaggeratedly strong, sustained motor responses typical of spasticity [23, 24, 26]. In a series of publications, we have established that the persistent Na^+^ current (*I*_NaP_) is largely expressed in the spinal locomotor network [27–32] and plays a critical role in generating self-sustained spiking activity in motoneurons [30]. SCI upregulates *I*_NaP_, leading to muscle spasms and hyperreflexia in both humans and adult rats [33–39]. In addition, a concomitant synaptic disinhibition of motoneurons due to a decrease in the levels of the main chloride extruder KCC2 also takes place after SCI in both humans and adult rodents [40–42]. We recently established that calpains, which are Ca^2+^-activated proteases recruited after SCI, typically cleave both sodium (Nav) channels, increasing *I*_NaP_, and KCC2, disinhibiting motoneurons [18]. We have therefore assumed that calpains together with KCC2 and *I*_NaP_ might be suitable therapeutic targets for reducing spasticity after SCI. Interestingly, we have established that low concentrations of riluzole specifically and potently block *I*_NaP_, and we have provided strong preclinical evidence in favor of the application of this drug to chronic SCI subjects [18, 38]. More specifically, acute administration of a single dose of riluzole reduces spasticity in rats with chronic SCI. Similar effects have been observed in humans with chronic SCI [39]. Therefore, the chronic administration of riluzole seems to be a potentially useful form of oral medication for obtaining long-lasting relief of spasticity in patients with SCI. The repurposing process should be facilitated as rilutek (the brand name for riluzole) is the only disease-modifying drug approved so far for attenuating neurodegeneration in amyotrophic lateral sclerosis [43]. In addition, a successful phase 1 clinical trial has shown that riluzole taken orally is well tolerated in patients with acute SCI [44–46]. The European Medicines Agency has given Riluzole the status of orphan drug for the treatment of spasticity following SCI. To provide an ever-broadening range of treatment options for reducing spasticity after SCI, we have designed the phase 1b/2b clinical study protocol (RILUSCI) presented here in order to examine the safety and efficacy of orally administered riluzole in patients with chronic SCI.

## Methods and analysis

### Study aims

The primary objectives of the trial will be to define the minimal effective dose of riluzole for treating spasticity, and to assess how effective it is as a means of reducing spasticity. The secondary objectives will be to examine the possible dose/effect relationship and to determine the safety and the pharmacokinetic profile of riluzole, as well as its impact on muscle spasms, pain, health-related quality of life and the mobility of SCI participants.

### Study design

**Fig 1:**
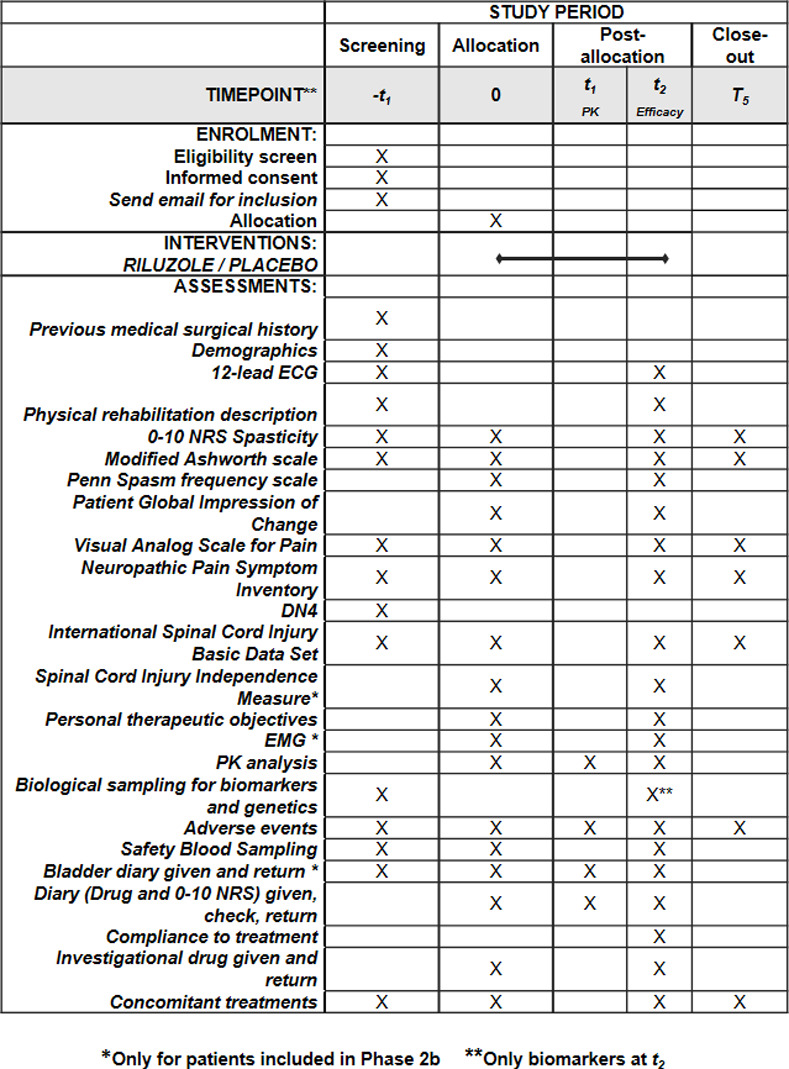
Standard protocol items recommended for interventional trials (SPIRIT). Schedule of enrolment, intervention and assessments. The study schedule will be the same in step 1 and in step 2 and it includes the following visits and clinical examinations. Patients with chronic SCI will undergo a screening period (v1) with a maximum duration of 2 weeks and then they will be randomized to one of the two groups (v2): riluzole twice daily or placebo twice daily for a 2 weeks period. Three follow up visits are planned: at day3 after the beginning of treatment for PK analysis (v3), and at the end of the 2 weeks treatment period (v4) and one week after withdrawal of study medication (v5).

**Fig 2:**
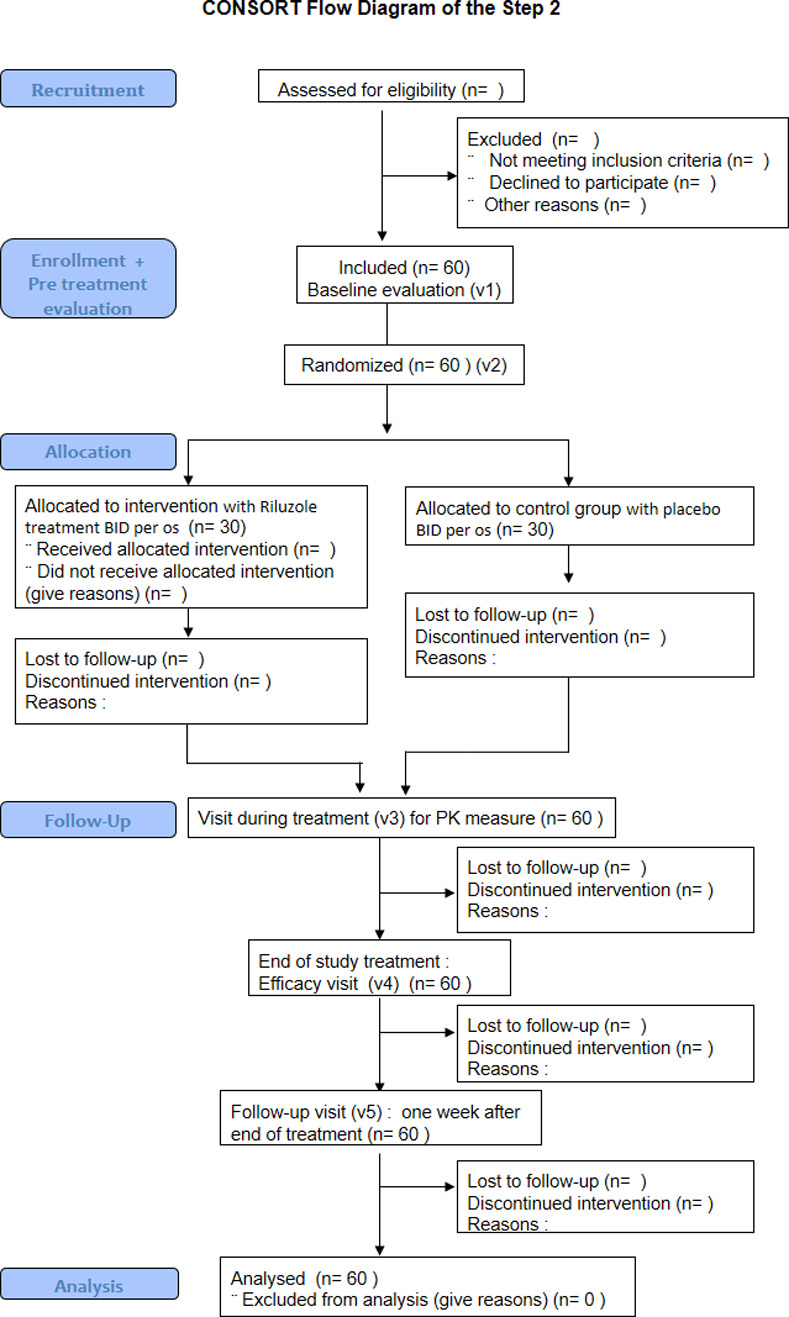
CONSORT diagram.

RILUSCI is an adaptive phase 1b–2b trial conducted on spastic patients with chronic SCI in order to determine the potential benefits of administering riluzole as compared with placebo. The protocol has been designed in keeping with the SPIRIT and CONSORT statements (see S1 Checklist: CONSORT 2010 and S2 Checklist: SPIRIT 2013 checklist). The SPIRIT schedule on enrolment, interventions and assessments is presented in **Fig 1**. **Fig 2** is a flowchart of the participants in the study based on the CONSORT guidelines [47].

Phase 1b will be a single-center double-blind trial involving a continuous reassessment method based on Bayesian inferences. The principle underlying this method is to identify the appropriate drug dosage required to obtain a level of efficacy which resembles as closely as possible a predetermined target level of efficacy in the population. The aim will be to determine the minimal effective dose (MED) of riluzole recorded in 75% of the patients tested. The phase 1 trial will be conducted at the Physical Medicine and Rehabilitation departments of Marseille’s public hospital group.

Phase 2b will be a parallel placebo-controlled multicentre randomised double-blind trial designed to compare the effects of a BID per os treatment with riluzole (daily dose to be defined during Phase 1b) *versus* placebo in participants with chronic SCI. Double-blind conditions will apply to both participants and investigators. The phase 2b trial will be conducted at 7 hospitals all over France. The participating centres include the Physical Medicine and Rehabilitation departments of Marseille’s public hopitals and the Valmante University Institute of Rehabilitation in Marseille (UGECAM), the University Hospitals of Limoges, Montpellier, Nantes, the Propara Rehabilitation centre in Montpellier, and the Saint Martin Rehabilitation Centre in Marseille.

### Participants and recruitment

Participants will be screened and recruited among outpatients consulting for SCI at the various centres participating in the trial during a period of 36 months. Patients’ associations will be contacted by the OrphanDev network (FCRIN) and patients referred by these associations will benefit from a phone interview to check the main inclusion/non-inclusion criteria before being referred to the nearest investigation centre. Participants must meet the following inclusion criteria at the time of the screening visit:

- Chronic traumatic spinal cord injury defined as:
  ◦ Lasting for at least 12 months
  ◦ Level C4-T12
  ◦ Complete or incomplete SCI (ASIA A, B, C, D)
  ◦ With spasticity (MAS > 1 and <5 on adductors and/or sural triceps and NRS > 4
- Male or Female patients affiliated to the French national health system
- Patients aged between 18 and 65. The rationale for limiting the participant’s age to 65 was the poor functional recovery in older individuals [48].
- Judged by the investigator to be capable of undergoing all the assessments completing all the questionnaires used in the study
- The last injection of BTX-A must have occurred more than 3 months before taking part in the trial and the participants must have regained a comparable level of spasticity to their previous level.
- The last intrathecal injection of baclofen or the intake per os of any other muscle relaxant must have taken place more than 14 days previously (phase 1b) or be stable for at least 30 days before the screening visit (phase 2b) and must remain stable throughout the protocol.
- The intake of all other medications, including painkillers, must have been stable for at least 30 days prior to the screening visit.
- The number of rehabilitation sessions and the methods used should not vary during the 15 days prior to the screening visit.
- Participants must give their prior informed written consent to the procedure used in the trial.

The exclusion criteria include:

- Occurrence of spinal cord injury less than 12 months previously,
- Any associated brain lesion liable to be the cause of spasticity,
- MAS≤1 or = 5 in at least adductor muscles and/or sural triceps muscles or NRS < 4
- Presence of urinary infection, fever, pressure ulcer or other spasticity-aggravating factors.
- Presence of other significant neurological or mental disorder or other illness liable to prevent accurate evaluation,
- Recent history (less than 1 year) of chemical substance dependency or any significant psychosocial disturbance,
- Insufficient fluency in the local language for the patient to be able complete neuropsychological, global and spasticity assessments.
- Active liver disease or clinically proven jaundice
- Active malignancy or history of invasive malignancy within the last five years
- Clinically significant neutropenia in the investigator’s opinion, Liver enzymes (ALT/SGPT or AST/SGOT) twice the upper normal limit at screening visit, Baseline elevations in several liver function tests (especially elevated bilirubin) which are clinically significant in the investigator’s opinion.
- AIDS or AIDS-related complex,
- The systolic blood pressure measurements > 190 or < 85 mm Hg and/or the diastolic blood pressure measurements > 105 or < 50 mm Hg at screening.
- An abnormal ECG at screening judged by the site investigator to be clinically significant.
- Treatment with any investigational drugs or devices less than 60 days before screening
- Any myorelaxant medication including intrathecal baclofen taken by the subject during the last 14 days prior to screening (step 1)
- Patient’s condition not stable under intrathecal baclofen or per os myorelaxant medication for at least 30 days prior to screening (step 2)
- Patient’s condition not stable under any other chronic medication, including analgesics, for ≥ 30 days prior to screening.
- Injection of BTX-A into striated muscle less than 3 months previously (apart from BTX-A injection into the detrusor)
- Subject is currently using, and will continue to use for the next 14 days, any of the following medications, which are classified as Inhibitors of CYP 1A2 (diclofenac, diazepam, nicergoline, clomipramine, imipramine, fluvoxamine, phenacetin, theophylline, amitriptyline and quinolones) or inducers of CYP 1A2 (rifampicin and omeprazole)
- Ongoing pregnancy and women with childbearing potential not using any form of efficacious contraception during the study and 3 months after the end of the study.
- Ongoing lactation and during the 3 months after the end of the study.
- Known hypersensitivity to riluzole

After checking the inclusion and exclusion criteria, each participant will receive extensive information about the treatment tested, the assessment procedures, the objectives and risks associated with the trial. Prior to the start of medication in the study, participants’ informed consent must be obtained in writing by the investigator. Subjects will be free to withdraw from the study at any time without having to give any explanations or being penalized in any way.

### Justification of sample size

In phase 1b, we will use an adaptive Bayesian design in order to minimize the number of subjects to be included. Because of the characteristics of the continuous reassessment method with Bayesian inferences, the inclusion of 30 subjects has been found to suffice for studies of this kind [49, 50]. Stopping rules will be applied sequentially to stop further inclusions before this number is reached. In phase 2b, the expected number of participants is arbitrary because it depends on the opportunities for recruitment at all the centers involved in the study, and the lack of knowledge about the success rate of riluzole is likely to result in a sample size which is too large for a frequentist approach to be used. The number of participants included during 24 months will be 30 participants per randomized group (i.e., 60 patients in all). Participants in phase 1b might also participate in phase 2b.

### Trial protocol

The timeline of the trial summarized in **Fig 1** will be the same in both 2 phases of the study. Subjects will participate for up to 5 weeks. The first 2 weeks will constitute the screening period, starting on the day the subject signs the Informed Consent Form. At the end of the screening period, participants will be randomized (1:1) to either the riluzole or the placebo groupsfor a 2-week period of treatment. Block randomisation stratified per center will be performed. A 1-week post-treatment period will ensue. It is planned to carry out three follow up visits on each participant: on day 3 after the beginning of treatment, at the end of the 2-week period of treatment, and one week after withdrawal of the medication. Details of the assessments and interventions occurring at each visit are presented in **Fig 1**.

At the screening visit, experimental drugs prepared by the coordinating pharmacy will be distributed to the investigating pharmacy at each study site. For phase 1b, riluzole oral suspension (5 mg/ml) will be prepared in 300 ml vial + syringe. For phase 2b, riluzole oral suspension will be diluted in Orablend SF* with a view to administering the same volume (20 ml) of oral suspension to each group. The investigating pharmacy will prepare clinically indiscernible galenic forms of riluzole and placebo. After being prescribed by the investigator, the treatments will be dispensed to patients at the second visit. All the documents required for drug traceability will be completed by the investigating pharmacist. The pharmacist will report the name of the drug, the dosage and the specific number of allocated drugs dispensed to each patient.

In phase 1b, four doses of riluzole will be tested: 25 mg BID, 50 mg BID; 75 mg BID, and 100 mg BID. The four dosage levels were chosen based on previous studies on amyotrophic lateral sclerosis and acute SCI patients treated with riluzole in the standard regimen [46]. The starting dose of riluzole will be randomly selected by the CIC-EC 1426/FCRIN PARTNERS platform. This dose will be communicated only to the pharmacist responsibe for preparing the experimental treatment in order to ensure the blinding of the investigator and the participant. Patients will take the treatment orally for 2 weeks, twice a day (morning and evening), before meals since co-ingestion of the drug with food can reduce the absorption of the drug by up to 20% [44]. The one-week follow-up period will allow for the elimination of riluzole between phases 1b and 2b. Each response to the treatment will be communicated to the biostatistician in charge of performing the Bayesian analysis. The following doses will be allocated, depending on the results of the statistical analysis performed. Dose level assignment will be determined by the CIC-EC 1426/FCRIN PARTNERS platform. Every two inclusions and in view of the outcomes, the success rates will be updated. Clinicians have attributed initial success rate predictions to each dosage, in the light of their clinical experience and the information available in the literature. The following initial predictions have been made: a dose of 50 mg/day: a success rate of 10%; 100 mg/day: 50%; 150 mg/day: 75%; 200 mg/day: 85%. A target probability of success of 75% will be adopted.

During phase 2b, the minimal effective dose defined in phase 1b will be administered to the patients. The CIC-EC 1426/FCRIN PARTNERS platform will inform the pharmacist (at the Timone Hospital in Marseille) about the treatment to be prepared and delivered to each participant. Patients included in the treatment group will take riluzole orally, BID, for 2 weeks at the established ME dose. The same administration schedule will be applied to the placebo patients. Each investigator, participant and pharmacist will be blinded about the identity of the participants in the intervention.

In phase 1 and 2, two 5-ml blood samples for the riluzole PK study and baclofen observance will be consistently collected prior to riluzole intake and 2 h postdose in order to determine the trough and peak concentrations, respectively, on days 0, 3 and 14 after the initial dose (amounting to 6 samples/participant in all). If possible, samples will be sent on the same day to the laboratory in charge of the pharmacokinetic tests. If this is not possible, all the samples will be frozen and stored at -20°C after the pre-analytic process.

All the drugs currently used to treat chronic SCI participants will be recorded, specifying: the name, dose, regimen, dates of start and end of intake. In step 1, participants will need to stop oral myorelaxant medication 14 days prior to screening and during the study. In this step 2, oral myorelaxant medication of participants should be stable for at least 30 days prior screening. Other treatments, including analgesics should be stable for ≥ 30 days prior to screening. Due to the risk of drug interactions, any drugs that interfere with CYP1A2 will be withdrawn at least 5 half-lives before the patients’ inclusion.

### Outcome measures

The primary outcome will be the efficacy of riluzole as a means of reducing spasticity. The description of this main outome will be binary (success or failure). Success has been defined as follows: “Improvement of the Modified Ashworth Scale (MAS) score by more than 1 point, or improvement of the 0–10 Numerical Rating Scale (NRS) spasticity score by more than 20% between Week 0 and Week 2”.

- The MAS is the most commonly used clinical method of measurement for assessing spasticity [1]. This score measures the resistance perceived by the rater when passively moving a joint through its full range of movement. MAS scores will be assessed by an experienced clinician on at least the adductor and/or triceps surae muscles at screening, randomization, end of treatment and follow-up visits [51]. The Minimal Clinically Important Difference in MAS lower limb muscles is 0.73 [52].
- The 0–10 NRS score (0 = “no spasticity” to 10 = “wo8rst possible spasticity”) is a participant-reported outcome measure designed to reflect participants’ perception of their spasticity. This score has been previously used on SCI participants and has been validated for Multiple Sclerosis spasticity assessment purposes [53]. The 20% cut-off was chosen because the Minimal Clinically Important Difference in the NRS is 18% [53]. The 0–10 NRS score will be assessed by each participant at screening, randomization, during the treatment period (every 24 hours) and at the follow up visits. The patients will be asked to record their scores in the “drug and 0–10 NRS” diary.

These two scales assessed by either the participant (NRS) or the clinician (MAS) will allow us to have a clearer picture and a greater sensitivity to the changes in spasticity before versus after the treatment period.

Secondary outcomes will include neurological, functional, and pain outcomes such as:

- Penn Spasms Frequency Scale: This is a self-reported perception of patients’ spasticity based on frequency ratings (from 0 = no spasms to 4 = spasms occurring more than ten times per hour) and severity ratings (1 = mild to 3 = severe) of their spontaneous muscle spasms. This tool is widely used to complement clinical assessments of spasticity and provide a more comprehensive understanding of an individual’s spasticity status [54].
- Participant Global Impression of Change: This scale is a 7-point categorical scale rated by the participant from 1 (very much improved) to 7 (very much worse). Participants are asked to assess the overall change in their condition since joining the study.
- Visual Analog Pain Scale: This scale is an 11-point continuous scale rated by participants from 0 (no pain) to 10 (worst possible pain).
- Neuropathic Pain Symptom Inventory: This questionnaire can be used by clinicians to classify various types of neuropathic pain and assess the changes induced by pharmacological treatment. This scale includes: 10 descriptors of various symptoms and 2 items quantifying the duration of spontaneous ongoing and paroxysmal pain. A total intensity score can be calculated as the sum of the scores obtained on the 10 descriptors [55].
- DN4 questionnaire: This questionnaire can be used by clinicians to discriminate between nociceptive and neuropathic pain. This scale has been validated with a high level diagnostic accuracy for use on individuals with SCI [56]. A score of three or more indicates neuropathic pain [57]. This questionnaire will be applied at the screening visit.
- International Spinal Cord Injury Basic Pain Data Set: This questionnaire contains a minimal amount of clinically relevant information about pain that can be collected in the everyday practice of healthcare professionals’ with experience of SCI. This questionnaire applies to all the effects of pain (how it interferes with patients’ day-to-day activities, their overall mood, and their ability to get a good night’s sleep) rating from zero (no interference) to six (extremely strong interference), specifying the number of pain-related problems and a description of the worst 3 [58].
- Spinal Cord Injury Independence Measure: This measure addresses three specific types of functional problems in participants with SCI, including an assessment of self-care problems (feeding, grooming, bathing, and dressing), respiratory and sphincter management, and the participant’s mobility levels (getting in and out of bed and going indoors/outdoors) [59]. The participant’s score is rated here by the clinician.
- Personal therapeutic objectives: Personal objectives will be determined at baseline (e.g. Adductor muscle spasticity, etc.) by the clinician along with the participant.
- Electrophysiology: A nerve conduction study and an Electromyography (EMG) will be performed using standard techniques. The tibial nerve will be stimulated at the ankle and popliteal fossa, and compound muscle action potentials (M-Wave) will be recorded over the abductor hallucis. Measurements include negative peak duration, baseline-to-peak amplitude, mean F-wave amplitude and soleus H reflex. The maximal H-reflex and M-wave responses can be used to compare maximum H-reflex amplitude and maximum M-wave amplitude (Hmax/Mmax) ratios between subjects under different recording conditions. Surface EMG will be performed at experienced centers only. Subjects will be seated in an adjustable chair with their test foot securely strapped to an instrumented footplate attached to a mechanical fixture. Surface electrodes will be attached to the skin above the muscle in order to measure the activity in the right and left tibialis anterior and soleus muscles. The EMG signal will be amplified and filtered (3Hz–3kHz, GRASS P511K). Electrophysiology examinations will be performed before drug intake and 2h post dose on Day 1 and Day 14.
- Riluzole pharmacokinetics (PK): Riluzole concentration in plasma is quantified by performing a high performance liquid chromatography assay with U.V. detection, adapted from Chow et al. [46], after a liquid-liquid extraction step from a 200-μl plasma sample. The assay is linear from 5 to 500 ng/ml, with a lower quantification limit of 5 ng/ml. For wash-out checking purposes, baclofen concentrations in plasma will be quantified by performing a liquid chromatography-tandem mass spectrometry assay (LC-MS/MS) after a solid-liquid extraction step. The assay is linear from 2 to 200 ng/ml, with a lower quantification limit which has been validated at 1 ng/ ml. The plasmatic concentrations of baclofen after IT administration can be expected to be low [60]. All the participants enrolled in the study will be asked to donate optional blood samples. These specimens will be used for research purposes to identify and/or check the presence of any inherited DNA polymorphisms known or thought to be associated with riluzole activity, which will be taken for DNA extraction from relevant participants after receiving their genetically informed consent at the predose stage.
- Safety and tolerability: to be assessed by the occurrence of Adverse Events (AE). All suspected unexpected serious AE are to be notified immediately to the sponsor.
- Laboratory safety parameters: Participants will undergo laboratory screening analysis including haematology (red blood cell count, Hematocrit, Hemoglobin, white blood cell count, platelets), biochemistry (sodium, potassium, chloride, calcium, phosphorus, lactic dehydrogenase, creatine phosphokinase, alkaline phosphatases, amylase, lipase, gamma-glutamyl transferase, total and conjugated bilirubin, creatinine, urea, uric acid, glucose, albumin, total proteins, total cholesterol, triglycerides), serologies (hepatitis B antigen, Hepatitis C antibodies, Anti-HIV1 and Anti HIV2 antibodies) and urinalysis (proteins, glucose, blood, ketone bodies, pH). All analyses will be performed by a local laboratory.
- Bladder diary: The participants will record in a diary the frequency of urinary probing and urinary leakages during 1 day during the 3 days preceding visit 2 (randomization) and during the 3 days preceding visit 4 (end of treatment).

### Risks and unblinding procedures

Results of previous studies on patients with amyotrophic lateral sclerosis have shown that the overall tolerability of riluzole is good [61] and that the drug can be used on all patients except for those with elevated transaminase levels or active liver disease. The most frequently encountered AEs are asthenia, gastrointestinal disorders, nausea and dizziness, which were more frequent at the 200-mg dose. The occurrence of these same AEs, although at a lower frequency, has also been reported in Phase IV observational studies and pharmacovigilance surveys. No unexpected AEs clearly associated with riluzole have been observed. A risk less commonly reported, but still very serious, is that of neutropenia, and physicians should watch out for this risk.

If any cases arise in which the spasticity worsens under treatment, the investigator will search for an intercurrent condition that is likely to explain this aggravation, including: infection, lithiasis, fecaloma, bedsores, etc. In the case of patients undergoing intrathecal baclofen infusions (phase 2b), the investigator will first check the pump. These conditions have first to be treated. In cases where no specific condition has been identified or the spasticity does not improve after treatment of the intercurrent condition, the administration of the experimental drug will be stopped.

Unblinding procedures: the need to unblind participants to their status can arise in the following cases:

- To make decisions about a patient’s clinical treatment or when an unexpected serious AE occurs and the intervention must be made known. If required, the investigator may apply to the sponsor for unblinding, with the coordinator’s approval,
- At the request of the Data Safety Monitoring Board,
- During an unmasked analysis in keeping with the study analysis plan.

Only the FCRIN platform and the clinical trial’s pharmacy team at the Timone hospital will be unblinded and will handle the unblinding procedures.

### Data management

In this trial, relevant data, AE, and safety assessments will be documented in both paper and electronic versions of the case report forms. Each participant has a unique numeric identifier. Data will be recorded and managed using a dedicated web-based software REDCap (Research Electronic Data Capture) with secured and restricted access. No personal data will be recorded in the case report forms apart from the unique numeric identifier. Access to the complete final trial dataset will be restricted to the clinical research unit’s statistician, who will analyse the study data for the purpose of reporting and publication of the results.

### Monitoring

The Data safety monitoring board, with no interests in either the drug tested or research on this drug, will be set up by the sponsor and entrusted with the mission of following up the security data. The Data safety monitoring board will also have an advisory function on points of security such as the tolerance and the reassessment of the benefit-to-risk ratio during the research process. This committee will also be responsible for making sure of the conformity of the protocol with current standards. The DSMB will meet at the end of Step 1. In the event of any anomaly, this committee will meet again as appropriate.

### Statistical analysis

Bayesian analysis will be performed by the CIC-EC 1426/FCRIN PARTNERS platform. Secondary analysis and safety analysis will be performed by the CIC-CPCET. Quantitative variables will be expressed as medians (quartiles) or means (standard deviations), depending on whether or not there is a Gaussian distribution. Qualitative variables will be expressed as numbers (percentages). Inter-group comparisons will be conducted using parametric or non-parametric tests, depending on the nature and the distributions of the variables. Unless otherwise specified, statistical significance is defined here as p<0.05. Baseline value will be the last value recorded before randomization. In phase 1b, all participants with whom the primary endpoint has been reached will be analysed with a view to applying the continuous reassessment method. In phase 2b, the probability of a response being associated with the minimum effective dose will be estimated using a Bayesian approach according to a beta-binomial model. Statistical analyses will be performed sequentially per participant at the end of the 2-week assessment period.

### Ethical approval and dissemination policy

This study has received the approval of the local ethical committee (CPP number 2016-000901-35 on May 31, 2016) and the authorization of the National Agency for Medicines and Health Products in France on July 10, 2017, as registered in the French National Clinical Portal https://clinicaltrials.gov (n° NCT02859792). The trial will be conducted in keeping with the Helsinki Declaration [62]. The reporting of the study will adhere to the Standard Protocol Items Recommendations for Interventional Trials (SPIRIT) [63]. The CONSORT checklist and the SPIRIT checklist are available as S1 and S2 Checklists. The results will be disseminated via publications in the scientific literature, oral and poster presentations by partners at international scientific meetings, wide-audience media and associations of patients and their families. The confidentiality of the research information about patients will be ensured by the people in charge of the quality control of biomedical research projects (article L.1121-3).

## Discussion

Recent developments have started in the US, focusing on the acute phase of SCI on the basis of the potential neuroprotective activity of riluzole. A phase 1b has been completed (NCT00876889) and a phase 2b/3 double-blinded randomized controlled trial is already in progress (NCT01597518/ RISCIS) [64]. The phase 1b study [45, 46] has shown that the riluzole PK was linear but not stationary (plasma concentration on day 14 < day 3) and that the inter-individual dispersion was high. The PK data published on amyotrophic lateral sclerosis, SMA and SCI have also suggested the existence of possible differences depending on the condition. Vegetative changes in acute SCI and/or PK interactions due to changes in CYP1A2 activity might partly explain this variability. No concentration-dependent responses or dose/response relationships have been observed so far with riluzole, and establishing PK/PD and K/PD relationships with riluzole in patients with SCI is a clinical challenge which still remains to be met.

The most unique aspect of the present RILUSCI trial in comparison with other registered trials is the fact that it involves the administration of riluzole to patients in the chronic phase of SCI. This is also the first time that the chronic administration of riluzole will be studied in patients suffering from spasticity. By acting on voltage-dependent sodium channels, riluzole decreases the persistent sodium current which, in the absence of presynaptic inhibition, favours neuronal hyperexcitability and therefore spasticity after SCI. The main advantage of riluzole is that it is already authorised and marketed in France for the treatment of amyotrophic lateral sclerosis [61] and cerebellar ataxias such as Friedreich’s ataxia Romano [65], and therefore its tolerance and AE have been well established. To our knowledge, this is the first pharmacological study on the efficacy of various doses of riluzole for treating spasticity in such a large sample of spastic patients after SCI. One of the most original features of this study is that it deals with the safety and efficacy of this molecule on spasticity, using an adaptive design to determine the optimal dose. Another strength of this study is the existence of a double blind, involving the investigators and participants, and even a triple blind, since the pharmacist responsible for preparing and delivering riluzole knows what doses have to be prepared, but is unaware of the identity of the participants, who have been given pseudonyms by the investigator on all the study documents. One of the main limitations of this study is the fact that the strict exclusion criteria have complicated and slowed down the rate of inclusion. In conclusion, the final goal of the present study is to propose a new treatment for spasticity in patients with SCI in the chronic phase.

## Supporting information

S1 File Public summary of opinion on orphan designation, European medicine agency. (PDF)

S2 File Detailed statistical analysis. (DOCX)

S3 File Original protocol. (DOC)

S4 File Model consent form and other related documents given to participants. (DOCX)

S1 Checklist CONSORT 2010 checklist of information to include when reporting a randomized trial. (DOC)

S2 Checklist The SPIRIT 2013 checklist. (DOC)

## Abbreviations

AE: Adverse Event
BID: bis in die, i.e. twice daily
DN4: questionnaire Douleur Neuropathique 4 questionnaire
EMG: Electromyography
*I*NaP: persistent sodium current
MAS: modified version of the Ashworth scale
MED: minimal effective dose
NRS: Numerical Rating Scale
PD: pharmacodynamics
PK: pharmacokinetics
SCI: Spinal cord injuries
