# Supplementary material for: Riluzole for treating spasticity in patients with chronic traumatic spinal cord injury: Study protocol in the phase ib/iib adaptive multicenter randomized controlled RILUSCI trial
Source: PLoS One. 2023 Jan 20;18(1):e0276892. doi: 10.1371/journal.pone.0276892 (PMC9858801; doi:10.1371/journal.pone.0276892)
Supplement: S1 File — (PDF) [file pone.0276892.s001.pdf]

23 February 2015
EMA/COMP/744269/2014
Committee for Orphan Medicinal Products

Public summary of opinion on orphan designation

Riluzole for the treatment of traumatic spinal cord injury

On 16 December 2014, orphan designation (EU/3/14/1401) was granted by the European Commission to Dr Laurent Vinay, France, for riluzole for the treatment of traumatic spinal cord injury.

What is traumatic spinal cord injury?

Traumatic spinal cord injury is damage to the spinal cord caused by an accident, such as a blow to the back. Injury to the spinal cord can damage the nerves that run through the cord and branch out from it. This can stop the flow of nerve impulses between the brain and the rest of the body, resulting in the loss of sensation, paralysis and even death, depending on the severity and location of the injury.

Many patients also experience spasticity (or muscle stiffness and spasm) and neuropathic pain (pain due to nerve damage).

Traumatic spinal cord injury is a life-threatening disease that is debilitating in the long term, because it can cause paralysis of the arms and legs, and reduces life expectancy.

What is the estimated number of patients?

At the time of designation, traumatic spinal cord injury affected approximately 3 in 10,000 people in the European Union (EU). This was equivalent to a total of around 153,000 people[*], and is below the ceiling for orphan designation, which is 5 people in 10,000. This is based on the information provided by the sponsor and the knowledge of the Committee for Orphan Medicinal Products (COMP).

What treatments are available?

At the time of designation, methylprednisolone (a steroid) was authorised for the treatment of spinal cord injury in some countries in the EU. Methylprednisolone reduces the inflammation and pressure on the spinal cord that can occur after it is damaged. In addition various medicines were used for the treatment of spasticity (baclofen, dantrolene, diazepam and tizanidine) and for neuropathic pain

[*]Disclaimer: For the purpose of the designation, the number of patients affected by the condition is estimated and assessed on the basis of data from the European Union (EU 28), Norway, Iceland and Liechtenstein. This represents a population of 511,100,000 (Eurostat 2014).

30 Churchill Place ● Canary Wharf ● London E14 5EU ● United Kingdom
Telephone +44 (0)20 3660 6000 **Facsimile** +44 (0)20 3660 5555
Send a question via our website www.ema.europa.eu/contact

An agency of the European Union

© European Medicines Agency, 2015. Reproduction is authorised provided the source is acknowledged.

(amitriptyline, lamotrigine, pregabalin and tramadol). Patients with spinal cord injury also had decompression surgery to reduce the pressure on the spine.

The sponsor has provided sufficient information to show that riluzole might be of significant benefit for patients with traumatic spinal cord injury. Data from clinical studies have shown that riluzole could improve symptoms of spasticity in these patients. This assumption will need to be confirmed at the time of marketing authorisation, in order to maintain the orphan status.

How is this medicine expected to work?

Riluzole is expected to reduce pain and muscle stiffness in patients with traumatic spinal cord injury by blocking sodium channels on the surface of nerve cells. These sodium channels play a role in the activating nerves responsible for spasticity and feeling pain. By blocking them, the medicine helps to reduce these effects. In addition the medicine affects other mechanisms that affect transmission of signals in the nervous system, which may contribute to its action in the condition.

Riluzole is already approved in the EU for the management of amyotrophic lateral sclerosis, a disease of the nervous system.

What is the stage of development of this medicine?

The effects of riluzole have been evaluated in experimental models.

At the time of submission of the application for orphan designation, clinical trials with riluzole in patients with traumatic spinal cord injury were ongoing.

At the time of submission, riluzole was not authorised anywhere in the EU for traumatic spinal cord injury or designated as an orphan medicinal product elsewhere for this condition.

In accordance with Regulation (EC) No 141/2000 of 16 December 1999, the COMP adopted a positive opinion on 13 November 2014 recommending the granting of this designation.

____

Opinions on orphan medicinal product designations are based on the following three criteria:

- the seriousness of the condition;
- the existence of alternative methods of diagnosis, prevention or treatment;
- either the rarity of the condition (affecting not more than 5 in 10,000 people in the EU) or insufficient returns on investment.

Designated orphan medicinal products are products that are still under investigation and are considered for orphan designation on the basis of potential activity. An orphan designation is not a marketing authorisation. As a consequence, demonstration of quality, safety and efficacy is necessary before a product can be granted a marketing authorisation.

For more information

Sponsor's contact details:

Dr Laurent Vinay
Institut des Neurosciences de la Timone
Campus Santé Timone
27 bd Jean Moulin
13385 Marseille cedex 05
France
Tel. +33 4 91 32 40 51
Fax +33 4 91 32 40 56
E-mail: laurent.vinay@univ-amu.fr

For contact details of patients' organisations whose activities are targeted at rare diseases see:

- Orphanet, a database containing information on rare diseases, which includes a directory of patients' organisations registered in Europe;
- European Organisation for Rare Diseases (EURORDIS), a non-governmental alliance of patient organisations and individuals active in the field of rare diseases.

Translations of the active ingredient and indication in all official EU languages[1], Norwegian and Icelandic

Language	Active ingredient	Indication
English	Riluzole	Treatment of traumatic spinal cord injury
Bulgarian	Riluzole	Лечение на травматична увреда на гръбначния мозък
Croatian	Riluzol	Liječenje traumatskih ozljeda kralježnične moždine
Czech	Riluzol	Léčba míšního traumatu
Danish	Riluzole	Behandling af traumatisk rygmarvslæsion
Dutch	Riluzole	Behandeling van traumatisch ruggenmergletsel
Estonian	Riluzool	Traumaatilise seljaaju kahjustuse ravi
Finnish	Riluzole	Traumaattisen selkäydinvamman hoito
French	Riluzole	Traitement du traumatisme de la moëlle épinière
German	Riluzol	Behandlung traumatischer Rueckenmarksverletzungen
Greek	Ριλουζόλη	Θεραπεία τραύματος της σπονδυλικής στήλης
Hungarian	Riluzol	Traumás gerincvelő sérülés kezelése
Italian	Riluzole	Trattamento del trauma acuto della colonna vertebrale
Latvian	Riluzols	Muguras smadzeņu traumatiska bojājuma ārstēšana
Lithuanian	Riluzolas	Nugaros smegenų trauminio pažeidimo gydymas
Maltese	Riluzole	Kura ta' korriment trawmatiku tan-nerv qawwi li jgħaddi minn ġos-sinsla
Polish	Riluzol	Leczenie pourazowego uszkodzenia rdzenia kręgowego
Portuguese	Riluzole	Tratamento da lesão traumática da medula espinal
Romanian	Riluzol	Tratamentul leziunilor traumatice ale măduvei spinării
Slovak	Riluzol	Liečba traumatického poškodenia miechy
Slovenian	Riluzol	Zdravljenje travmatske poškodbe hrbtenjače
Spanish	Riluzole	Tratamiento de las lesiones espinales medulares traumáticas
Swedish	Riluzole	Behandling av traumatisk ryggmärgsskada
Norwegian	Riluzol	Behandling av traumatisk ryggmargsskade
Icelandic	Rílúzól	Meðferð mænuskaða vegna slyss

[1] At the time of designation
