# Supplementary material for: Riluzole for treating spasticity in patients with chronic traumatic spinal cord injury: Study protocol in the phase ib/iib adaptive multicenter randomized controlled RILUSCI trial
Source: PLoS One. 2023 Jan 20;18(1):e0276892. doi: 10.1371/journal.pone.0276892 (PMC9858801; doi:10.1371/journal.pone.0276892)
Supplement: S2 File — (DOCX) [file pone.0276892.s002.docx]

**S2 Statistical Analysis for more details**

Bayesian analysis will be performed by URC Robert Debré Hospital. Secondary analysis and safety analysis will be performed by the CIC-CPCET. Analyses will be conducted in respect of international guidelines and recommendations for clinical trials (ICH, CONSORT) and following internal Standard Operating Procedures. Quantitative variables will be described as medians (quartiles) or means (standard deviation) depending on the Gaussian distribution or not. Qualitative variables will be described as numbers (percentages). Inter-group comparisons will be conducted using parametric or non-parametric tests according to the nature and the distributions of the variables. Unless otherwise specified, statistical significance is defined as p<0.05. Baseline value will be the last value before randomization.

Pharmacokinetics parameters for riluzole will be calculated using standard Bayesian population approach using a reference population matrix already made available in the literature (Bruno et al., 1997). Stationarity of the PK parameters over the treatement (i.e., D0-D14) will be evaluated. Exposure levels (i.e., truncated AUCs) will be calculated. Pharmacokinetics will be studied using compartimental modelling with dedicated tools (KineticPro, MonoLix).

To assess the dose-response relationship of riluzole for SCI, the continual reassessment method (CRM) (O'Quigley et al., 1990) based on Bayesian inference. The aim will be to determine the minimal effective dose (MED) of riluzole defined as the dose level among the four chosen doses that had a final response probability closest to the target success rate of 75%. The main judgment criteria will be binary (success or failure). The CRM is sequential Bayesian method based on a one-parameter model, which aims at estimating the percentile of dose-response among k distinct dose levels d_i (i=1,...,4)_. Each one of the four dose levels is associated by the investigators (according to his/her personal experience and available data in the literature at the time of initiation of the trial) with a prior estimated success probability. Then, a one-parameter power model will be used to fit the dose-response curve, with an exponential prior distribution for the model parameter. The posterior response probability of each dose level will be re-estimated after each new inclusion of cohort patients (2 patients per cohort). The allocated dose to each new cohort of patients will be the dose level with the updated posterior response probability closest to 0.75. The first cohort of patients will receive a loading dose randomly chosen by the statistician with blinding of both patients and investigators. The decision to end the study will be based on stopping criteria, in order to detect whether all doses were likely to be inefficient or a suitable estimation of the MED has been reached. The study will be conducted in two steps: 1) Determination of the MED among the four doses of the panel 2) Estimation of the probability of response associated to the MED. After the inclusion of 30 patients, the minimum efficient dose will be the dose whose mean updated success rate closest to the target success rate of 75%. However, the first stage analysis can also be terminated earlier if there is high evidence that a specific dose will remain unchanged for the next two hypothetical groups of two further patients: *P(unchanged dose|data, z=4)>0.85, stop of the first stage and passage in second stage* or if no dose is estimated to be efficient enough *P(d_4_<target)>0.85* (Zohar and Chevret, 2001; O'Quigley and Reiner, 1998). If the first criterion is satisfied (after at least 10 patients’ outcomes) then the dose allocation process will be stopped and the second stage analysis will begin and if the second criterion is satisfied then the trial will be stopped. The second stage of analysis will ensure that the trial will not terminate too early if a minimum efficient dose is previously selected. Thus, the inclusions will be pursued at this dose level in order to obtain reliable estimates of the success rate. Again, the second stage analysis will be terminated after the inclusion of the total number of patients (n=30 per group) or if there is a high evidence that outcomes from another two hypothetical groups of two patients would not provide further gain on precision of the estimation of the success rate *P(max of the width of the credibility interval |data, z=4)<0.05*. Early termination of the study will be validated by the investigator, the sponsor and the independent monitoring board of the study.

To assess the efficacy of Riluzole compared with placebo on the spasticity of chronic SCI patients, a Bayesian approach will be used. The latter is to update the *a priori* information on the expected success rate from the observations. This produces an expected response rate revalued *a posteriori*. Statistical analysis will be performed sequentially after each observation of the response at two weeks of patients enrolled. It is to sequentially estimate the probability of observing a success using a Bayesian approach with a beta-binomial model (Berry D A. 1996) . The Bayesian approach is to consider the success rate as a random variable with prior density centered on the expected rate of success. The construction of the prior density is a combination of data from the literature and examination of a panel of experts, before the beginning of the trial. Two beta densities will be chosen, defined by two parameters a and b considering two types of opinion (i) optimistic and (ii) pessimistic from the elicitation of experts and literature. The *a priori* expected mean of each density will therefore centered on $\left(\pi=\frac{a}{a+b}\right)$based on those opinions. Several choices will be made for the variance given by $\left(\frac{ab}{\left(a+b\right)^{2}\left(a+b+1\right)}\right)$ allowing giving more or less weight on observations relative to the initial *a priori* on the rate of success (more the variance is large, more the weight of observations increases). The Bayesian estimator of the success rate is the expected value of the *a posteriori* distribution, whose parameters are defined after *n* inclusions by $a_{n}=a+r$ and $b_{n}=b+n-r$ in each arm, where *r* is the number of successes observed on *n* inclusions. So we obtain the following expected value in each arm: $\tilde{\pi}=\frac{a+r}{a+b+n}$. The difference between the response rates in both arms of randomization is modeled by the random variable $\Delta$. We will study the random variable $\Delta$, i.e. its distribution and credibility intervals as depending on optimistic and pessimistic views of the expertise. We will also calculate the predictive distribution of the number of successes over the next *k* inclusions or the remaining patients scheduled in each arm. Because of the relative small number of patients, there are no stopping rules based on Bayesian sequential analysis.

Changes and cores of secondary outcomes will be compared between the two treatment groups using a Student t-test or a Mann-Whitney test. Few score cannot be analyzed as continuous variable (severity of spasms in particular) and will be compared as frequencies using a McNemar test. The significance level is set at 0.05 in a two-sided situation.

Descriptive statistical analyses will be performed with the SAS 9.3 (SAS Inc, Cary, NC, USA) software package for PC and the Bayesian statistical analysis will be performed with R scripts written by URC Robert Debré (R version 3.5.2 (2018-12-20)).

References

Berry D A. Statistics: a Bayesian perspective. Duxbury, Belmont Californie 1996.

O'Quigley, J., Pepe, M., & Fisher, L. (1990). Continual reassessment method: a practical design for phase 1 clinical trials in cancer. Biometrics, 33-48.

O'Quigley, J., & Reiner, E. (1998). A stopping rule for the continual reassessment method. Biometrika, 85(3), 741-748.

Zohar, S., & Chevret, S. (2001). The continual reassessment method: comparison of Bayesian stopping rules for dose‐ranging studies. Statistics in medicine, 20(19), 2827-2843.
