# Supplementary material for: Riluzole for treating spasticity in patients with chronic traumatic spinal cord injury: Study protocol in the phase ib/iib adaptive multicenter randomized controlled RILUSCI trial
Source: PLoS One. 2023 Jan 20;18(1):e0276892. doi: 10.1371/journal.pone.0276892 (PMC9858801; doi:10.1371/journal.pone.0276892)
Supplement: S3 File — (DOC) [file pone.0276892.s003.doc]

| **“Riluzole for the treatment of spasticity in the traumatic chronic spinal cord injury condition: Adaptive, Multicenter, placebo-controlled, randomised, double blind trial in a Rare Disorder”**  **RILUSCI** |
| --- |

Biomedical research protocol

Version 3 en date du 26/06/2017

Project code: PHRC-15-0107 / No EUDRACT:N°2016-000901-35

Program Coordinator

**Professor Olivier BLIN**

Pharmacologie, Hôpital de La Timone, Assistance Publique Hôpitaux de Marseille

[olivier.blin@ap-hm.fr](mailto:olivier.blin@ap-hm.fr)

0491387565

Coordinating investigator:

**Professor Jean Michel VITON**

Médecine Physique et Réadaptation, Hôpital de La Timone, Assistance Publique Hôpitaux de Marseille

[Jean-michel.viton@ap-hm.fr](mailto:Jean-michel.viton@ap-hm.fr)

0491384616

Scientific Director

**Frédéric BROCARD (Ph.D.)**

Frederic.brocard@univ-amu.fr

Sponsor : AP-HM

Département de la Recherche Clinique et de l’Innovation (DRCI)

80 Rue Brochier, 13005 Marseille

[drci@ap-hm.fr](mailto:drci@ap-hm.fr)

Tél : 0491382747

Clinical research unit: CIC-CPCET

Pr Joelle Micallef

Courriel : joelle.micallef@ap-hm.fr

Tél : 0491387563

Methodology : F-CRIN Platform: PARTNERS

Dr Corinne Alberti

Courriel : Corinne.alberti@inserm.fr

Tél : 0140032465

Pharmacy : Unité d’expertise pharmaceutique et recherche biomédicale (UEPRB), AP-HM

Pr Stéphane HONORE, Dr Anita COHEN

Courriel : [anita.cohen@ap-hm.fr](mailto:anita.cohen@ap-hm.fr)

Tel : 0491384442

**Signature page of a biomedical research protocol**

**Title:** “**Riluzole in the traumatic chronic spinal cord injury condition in the treatment of spasticity: Adaptive, Multicenter, placebo-controlled, randomised, double blind trial in a Rare Disorder**”

**Project code :** PHRC-15-0107

Version N° 3-26/06/2017

| Coordinating investigator : |  |
| --- | --- |
| Jean Michel Viton  Hôpital Timone  Marseille |  |
| Investigator : |  |
| Name  Center  City |  |
| Sponsor Representative  Assistance Publique – Hôpitaux de Marseille  Direction de la Recherche Clinique et de l’Innovation  Rue Brochier  13005 Marseille |  |
|  |  |

This research obtained a favorable opinion of Independent Ethics Committee (CPP Sud Mediterrannée 1) the 13/04/2016 and an authorisation from ANSM the 10/07/2017.

Table des matières

[**1** **SYNOPSIS 7**](#__RefHeading___Toc477862024)

[2 Rationale of the research 17](#__RefHeading___Toc477862025)

[2.1 Hypothesis of research 17](#__RefHeading___Toc477862026)

[2.2 Background 17](#__RefHeading___Toc477862027)

[2.3 Summary of preclinical and clinical research 21](#__RefHeading___Toc477862028)

[2.4 Study population 23](#__RefHeading___Toc477862029)

[2.5 Experimental treatment 23](#__RefHeading___Toc477862030)

[2.6 Drug dosage, route, duration of experimental treatment. 24](#__RefHeading___Toc477862031)

[2.7 Justification of primary endpoint 25](#__RefHeading___Toc477862032)

[2.8 Summary of beneficial effects and risks for the patients during the protocol 25](#__RefHeading___Toc477862033)

[3 objectives 26](#__RefHeading___Toc477862034)

[3.1 Main objective 26](#__RefHeading___Toc477862035)

[3.2 Secondary objectives 26](#__RefHeading___Toc477862036)

[4 Design of the research 27](#__RefHeading___Toc477862037)

[4.1 Main and secondary end points 27](#__RefHeading___Toc477862038)

[4.1.1 Primary endpoint 27](#__RefHeading___Toc477862039)

[4.1.2 Secondary endpoints 27](#__RefHeading___Toc477862040)

[4.2 Study design 27](#__RefHeading___Toc477862041)

[4.2.1 Experimental design 27](#__RefHeading___Toc477862042)

[4.2.2 Number of centers 28](#__RefHeading___Toc477862043)

[4.2.3 Randomisation 28](#__RefHeading___Toc477862044)

[4.2.4 Blind modalities 28](#__RefHeading___Toc477862045)

[4.2.5 Unblinding procedures 29](#__RefHeading___Toc477862046)

[4.2.6 Validation procedures 29](#__RefHeading___Toc477862047)

[5 Study description 29](#__RefHeading___Toc477862048)

[5.1 Overall study description 29](#__RefHeading___Toc477862049)

[5.2 Study assessments and procedures 30](#__RefHeading___Toc477862050)

[5.2.1 Primary outcome measure (at 2 weeks): 30](#__RefHeading___Toc477862051)

[5.2.2 Secondary outcome measure (at 2 weeks): 31](#__RefHeading___Toc477862052)

[5.3 Visits in clinical department 33](#__RefHeading___Toc477862053)

[5.3.1 Visit 1: screening visit (V1) (in the 2 weeks preceeding randomisation) 33](#__RefHeading___Toc477862054)

[5.3.2 Visit 2: Day 1randomisation visit (V2) 34](#__RefHeading___Toc477862055)

[5.3.3 Visit 3: Day 4 PK Visit (V3) 35](#__RefHeading___Toc477862056)

[5.3.4 Visit 4: Day 14 Efficacy Visit (V4) 35](#__RefHeading___Toc477862057)

[5.3.5 Visit 5:follow-up visit (V5 Day 21) 36](#__RefHeading___Toc477862058)

[5.3.6 Chronological synopsis(for the 2 steps) 37](#__RefHeading___Toc477862059)

[5.4 Rules for stopping treatment or protocol 38](#__RefHeading___Toc477862060)

[6 Selection criteria 38](#__RefHeading___Toc477862061)

[6.1 Inclusion criteria 38](#__RefHeading___Toc477862062)

[6.2 Exclusion criteria 39](#__RefHeading___Toc477862063)

[6.3 Informed consent 40](#__RefHeading___Toc477862064)

[6.4 Recruitment modalities 41](#__RefHeading___Toc477862065)

[7 STUDy TREATMENTS 42](#__RefHeading___Toc477862066)

[7.1 Experimental drugs: name, description, administration and dosage 42](#__RefHeading___Toc477862067)

[7.2 Concomitant treatments 44](#__RefHeading___Toc477862068)

[7.3 Drugs interaction 45](#__RefHeading___Toc477862069)

[8 DRUG CONCENTRATIONS 45](#__RefHeading___Toc477862070)

[8.1 Drug Concentration Determination 45](#__RefHeading___Toc477862071)

[8.2 Plasma samples collection 46](#__RefHeading___Toc477862072)

[9 STATISTICAL METHODS 46](#__RefHeading___Toc477862073)

[9.1 Sample size justification 47](#__RefHeading___Toc477862074)

[9.2 Populations analysed 47](#__RefHeading___Toc477862075)

[9.3 Patient description 47](#__RefHeading___Toc477862076)

[9.4 Pharmacokinetics Analysis 48](#__RefHeading___Toc477862077)

[9.5 Statistical Analysis of primary endpoint (F-CRIN Platform, Dr Corinne Alberti) 48](#__RefHeading___Toc477862078)

[9.6 Statistical Analysis of secondary endpoints 50](#__RefHeading___Toc477862079)

[9.7 Safety analysis 51](#__RefHeading___Toc477862080)

[10 SAFETY – RISkS 52](#__RefHeading___Toc477862081)

[10.1 Description of laboratory safety parameters 52](#__RefHeading___Toc477862082)

[10.2 Management of adverse events 52](#__RefHeading___Toc477862083)

[10.3 Investigator’s liability 53](#__RefHeading___Toc477862084)

[10.3.1 Regulatory obligations of investigator (Art. R. 1123-54) 53](#__RefHeading___Toc477862085)

[10.3.2 Protocol particularities 54](#__RefHeading___Toc477862086)

[10.3.3 Notification to the sponsor by the investigator 54](#__RefHeading___Toc477862087)

[10.3.4 Notification period to the sponsor 55](#__RefHeading___Toc477862088)

[10.3.5 Sponsor’s role 55](#__RefHeading___Toc477862089)

[10.3.6 Annual safety report 55](#__RefHeading___Toc477862090)

[10.4 Data safety monitoring board (DSMB) 56](#__RefHeading___Toc477862091)

[11 DATA COLLECTION 57](#__RefHeading___Toc477862092)

[11.1 Data’s access 57](#__RefHeading___Toc477862093)

[11.2 Source documents and record retention 57](#__RefHeading___Toc477862094)

[11.3 Data confidentiality 57](#__RefHeading___Toc477862095)

[11.4 Data ownership 57](#__RefHeading___Toc477862096)

[12 Data management 58](#__RefHeading___Toc477862097)

[13 Quality contrOl 58](#__RefHeading___Toc477862098)

[13.1 General organization 58](#__RefHeading___Toc477862099)

[13.1.1 Opening strategy 59](#__RefHeading___Toc477862100)

[13.1.2 Monitoring of the centers 59](#__RefHeading___Toc477862101)

[13.2 Quality control 59](#__RefHeading___Toc477862102)

[13.3 CRF 60](#__RefHeading___Toc477862103)

[13.4 Study Deviations 60](#__RefHeading___Toc477862104)

[13.5 Audit / inspections 60](#__RefHeading___Toc477862105)

[14 ETHIc and LEGAl aspects 61](#__RefHeading___Toc477862106)

[14.1 Information of patient and consent collect 61](#__RefHeading___Toc477862107)

[14.1.1 Information of the study’s patients 61](#__RefHeading___Toc477862108)

[14.1.2 Patient’s consent 61](#__RefHeading___Toc477862109)

[14.1.3 Information in the medical records 62](#__RefHeading___Toc477862110)

[14.1.4 Modification of the informed consent form 62](#__RefHeading___Toc477862111)

[14.2 **Forbidden of participation IN other research** 62](#__RefHeading___Toc477862112)

[14.3 Recruitment 62](#__RefHeading___Toc477862113)

[14.4 Study taken charge 62](#__RefHeading___Toc477862114)

[14.5 Compensatory damages 62](#__RefHeading___Toc477862115)

[14.6 Legal obligations 63](#__RefHeading___Toc477862116)

[14.6.1 Sponsor’s role 63](#__RefHeading___Toc477862117)

[14.6.2 Investigator’s role 63](#__RefHeading___Toc477862118)

[14.6.3 Request of Independent Ethics Committee opinion (CPP according to French Law) 63](#__RefHeading___Toc477862119)

[14.6.4 Request of ANSM authorization 63](#__RefHeading___Toc477862120)

[14.6.5 Commitment of conformity to the CNIL « Méthodology of reference » MR 001 63](#__RefHeading___Toc477862121)

[14.7 Study’s modifications 63](#__RefHeading___Toc477862122)

[14.8 Clinical study report 63](#__RefHeading___Toc477862123)

[15 Financing and insurance 64](#__RefHeading___Toc477862124)

[15.1 Research’s budget 64](#__RefHeading___Toc477862125)

[15.2 Insurance 64](#__RefHeading___Toc477862126)

[16 publication’s rules 64](#__RefHeading___Toc477862127)

[17 ANNEXES 66](#__RefHeading___Toc477862128)

[17.1 List of participants 66](#__RefHeading___Toc477862129)

[17.2 Echelles 67](#__RefHeading___Toc477862130)

[17.2.1 Score d’Ashworth modifié 67](#__RefHeading___Toc477862131)

[17.2.2 NRS spasticity 67](#__RefHeading___Toc477862132)

[17.2.3 Patient global impression to change 68](#__RefHeading___Toc477862133)

[17.2.4 Echelle de Penn 68](#__RefHeading___Toc477862134)

[17.2.5 EVA Douleur 69](#__RefHeading___Toc477862135)

[17.2.6 Neuropathic Pain Symptom Inventory 70](#__RefHeading___Toc477862136)

[17.2.7 International Spinal Cord Injury Pain Basic Data Set (ISCIPBDS) 73](#__RefHeading___Toc477862137)

[17.2.8 Spinal Cord Independence Measure 77](#__RefHeading___Toc477862138)

[17.2.9 ASIA 82](#__RefHeading___Toc477862139)

[17.2.10 DN4 83](#__RefHeading___Toc477862140)

[18 BIBLIOGRAPHY 85](#__RefHeading___Toc477862141)

# SYNOPSIS

| **TITLE** | Riluzole in the treatment of spasticity in the traumatic chronic spinal cord injury condition: Adaptive, Multicenter, placebo-controlled, randomised, double blind trial in a Rare Disorder |
| --- | --- |
| **ACRONYM** | **RILUSCI** |
| **DEVELOPMENT PHASE** | Ib/IIb |
| **COORDINATING INVESTIGATOR** | Prof Jean Michel Viton  Service de Médecine Physique et de Réadaptation  Hôpital Timone  Marseille |
| **PROJECT COORDINATOR** | Prof Olivier Blin  Service de Pharmacologie Clinique  Hôpital Timone  Marseille |
| **SCIENTIFIC COORDINATOR** | Dr Frédéric Brocard (Ph.D.)  Institut Neurosciences Timone, Faculté Médecine, Marseille |
| **SPONSOR** | Assistance Publique – Hôpitaux de Marseille |
| **RATIONALE** | Spasticity is a very common symptom in patients with an upper motor neuron lesion. At present there is an unmet expectation of improvement in the quality of life for spinal cord injured patients in the chronic phase, due to spasticity and neuropathic pain, in addition to muscle weakness and bladder/bowel disturbances. Spasticity in chronic Spinal Cord Injury (SCI) is a debilitating condition with an impact on mobility, hygiene and daily activities. Spasticity can limit the transition from bed to chair, comfortable sitting position, and also make the routine hygiene difficult to maintain. It can also be accompanied by painful spasms and ultimately to muscle shortening and loss of range of motion. The current therapeutic arsenal consists mainly in the administration of oral benzodiazepines, intrathecal or oral baclofen, oral Dantrolène sodium, oral tizanidine or local Botulinum Toxin A (BTX-A) to reduce spasticity. Despite the approval of several drugs for the management of spasticity and pain related to SCI, the relative efficacy assessed by professionals and patients clearly highlight an unmet need in the therapeutic options. Therefore, the need to expand opportunities to identify an effective treatment for functional improvement of spasticity and neuropathic pain is a clinical reality.  Riluzole is currently on the market and indicated in Amyotrophic Lateral Sclerosis. Treatment with riluzole 100 mg/d has shown to be effective to increase life expectancy in ALS patients.  The effect of riluzole has been recently tested by our group in a rat model of T8 spinal cord injury. Results showed that riluzole (single intraperitoneal (IP) dose of 8 mg/kg or 8 mg/kg/day for 2 weeks) reduced spasticity (Brocard et al. unpublished data) and increases the mechanical pain threshold after spinal cord contusion.  Recent development has started in the US for acute Spinal Cord Injury (SCI) on the basis of potential neuroprotective activity of Riluzole. A phase Ib has been completed (NCT00876889) and a Phase II is in progress (NCT01597518/ RISCIS). In the phase Ib study, it has been shown that the Riluzole Pharmacokinetics (PK) was linear but not stationary (plasma concentration day 14 < day 3), and that the inter-individual dispersion was high. The PK data reported for amyotrophic lateral sclerosis, spinal muscular atrophy and SCI also suggest a possible difference related to the condition. Vegetative changes in acute SCI and/or PK interaction due to CYP1A2 activity changes might explain part of this variability.  Due to riluzole PK variability and absence of demonstrated dose or concentration/effect relationship, a dose ranging study is required to achieve confidence as regards to exposure and target engagement (Step 1 of the present study).  In Step 2 we will determine the efficacy of Riluzole in improving spasticity and neuropathic pain in chronic SCI condition. The Ashworth Scale (AS) is usually used to assess spasticity. It relies upon the examination performed by the physician. The 0-10 Numeral Rating Scale (NRS) score is an alternative method (auto-evaluation by the patient) to evaluate spasticity. It has been already used for SCI patients as secondary criteria (Sativex trial and Ultramicronized PEA Normast) and has been validated for Multiple Sclerosis spasticity assessment. In this study these two scales will be used to evaluate spasticity from a patient and investigator perspective in order to have a better evaluation and a higher sensitivity to spasticity changes before and after the treatment period. |
| **Main objective** | The study will be conducted in two steps: 1) Determination of the Minimal Effective Dose (MED) among the four doses of the panel 2) Estimation of the probability of response associated to the MED.Each step has a main objective:  **Step 1 Objective**: To determine a daily dose of Riluzole that improves spasticity in patients with chronic SCI  **Step 2 Objective**: To demonstrate, in a phase 2b trial, the efficacy of Riluzole to improve spasticity versus placebo, in patients with chronic SCI. |
| **Secondary objectives** | The secondary objectives of this study (step 1 and step 2) are the following:  To determine the safety of Riluzole in SCI patients  To determine the PK of Riluzole in SCI patients  To determine the pain relieving effect of Riluzole in SCI patients  To determine the effects of Riluzole on activities and participation in SCI patients  To determine the effects of Riluzole on bladder dysfunction |
| **Primary and secondary end points** | The primary end point of this study (step 1 and step 2) is defined as the improvement of Modified Ashworth Score better than 1 point, or 11 points Numerical Rating Scale (0-10 NRS) spasticity score better than 20% between Week 0 and Week 2.  The secondary endpoints of this study (step 1 and step2) are:  **Safety** : side effects  **Pharmacokinetics (PK)**: Blood sampling time points will be estimated using Limited Sampling Strategy (according to the selected galenic form and sex). Individual PK parameters will be calculated using standard compartimental approaches. In particular, exposure parameters (i.e., Cmax, Ctrough, AUC) will be evaluated.  **Electrophysiology**: H reflex, mean F wave amplitude, surface EMG at T0 (before drug intake) at T2h (2h after drug intake) (only in Step 2)  **Efficacy** : 0-10 NRS score, Modified Ashworth score (MAS) on at least adductor muscles and/or triceps surae, Patient Global Impression of Change, Penn Spasm frequency scale  **Pain** : Visual Analog Scales, Neuropathic Pain Symptom Inventory, International Spinal Cord Injury Basic Data Set (ISCIPDS)  **Activities and Participation:** Spinal Cord Injury Independence Measure (SCIM scale) (only in Step 2); Personal therapeutic objectives determined at baseline, Goal Attainment Scale (GAS) (Adductor muscle spasticity...)  **Bladder dysfunction:** Bladder diary (items on urinary frequency, daytime incontinence...) (Only in Step 2) |
| **Experimental design** | An Adaptive, phase 1b/2b multicenter, randomised, placebo-controlled, double blind trial of Riluzole in treatment of spasticity in patients with Spinal Cord Injury.  Step 1:To assess the dose-response relationship of Riluzole for spinal cord injury, the continual reassessment method (CRM) based on Bayesian inference with a modification in order to control outlier observations (Resche-Rigon, 2008) will be used. The aim will be to determine the Minimal effective dose (MED) of Riluzole in 75% of patients. The principle of this method will be to identify the adequate drug dosage to obtain a level of efficacy as close as possible to a predetermined target level of efficacy in the population. Four dosages of Riluzole will be tested from 50 mg to 200 mg/day and a target probability of success of 75% will be chosen. Dose will be reassessed every two patients, based on outcomes evaluated at the end of the 2-week treatment period. Each patient will be treated at the dose set for his cohort. The study will be double blind and the main judgment criteria will be binary (success or failure).  Step 2: The estimation of the probability of response associated with the resulting MED will be estimated in a phase IIb, comparative (Riluzole versus placebo), randomized, double blind trial. |
| **STUDY POPULATION** | Male and female patients with chronic SCI |
| **INCLUSION CRITERIA** | 1. Chronic traumatic SCI defined as:    1. At least a 12-month history of       1. C4-T12 traumatic SCI       2. Complete and incomplete ( AIS A,B,C,D)       3. With Spasticity (5>MAS>1 on at least adductor muscles and/or triceps surae muscles and NRS ≥ 4) 2. Male or Female 3. Aged 18 to 65 years at the time of screening 4. Judged by site investigator to be able to comply with evaluations at baseline and throughout the study 5. Last injection of BTX-A in striated muscle more than 3 months ago and patients must have returned to their level of spasticity before BTX-A injection 6. Last intrathecal (IT) injection of baclofen or per os administration of any myorelaxant should be more than 14 days ago (Step 1) 7. The dose of myorelaxant or Baclofen should be stable for ≥ 30 days prior to screening and kept at stable daily dose until the end of the protocol (Step 2). 8. Stable on all other chronic medications for ≥ 30 days prior to screening, including analgesics 9. Stable on rehabilitation (methods and frequency) for ≥ 15 days prior to screening 10. Written informed consent provided by subject |
| **EXCLUSION CRITERIA** | 1. Spinal cord injury of less than 12 months, 2. Associated Brain lesion that might be the cause of spasticity, 3. MAS≤1 or =5on at least adductor muscles and/or triceps surae muscles or NRS < 4 4. Presence of urinary infection, fever, pressure ulcer or other spasticity-aggravating factors. 5. Presence of other significant neurological or mental disorder or other illness, which would preclude accurate evaluation, 6. Recent history (less than 1 year) of chemical substance dependency or significant psychosocial disturbance, 7. Insufficient fluency in local language to complete neuropsychological, global and spasticity assessments 8. Active liver disease or clinical jaundice 9. Active malignancy or history of invasive malignancy within the last five years 10. Neutropenia, liver enzymes (ALT/SGPT or AST/SGOT) 2 times the upper limit of normal (ULN) at screening visit, baseline elevations of several liver function tests (especially elevated bilirubin). 11. AIDS or AIDS-related complex, 12. The systolic blood pressure measurement is > 190 or < 85 mm Hg and/or the diastolic blood pressure measurement is > 105 or < 50 mm Hg at screening. 13. The ECG is abnormal at screening and judged to be clinically significant by the site investigator. Particular attention will be given to any sign suggesting conduction disorders. 14. Treatment with any investigational drugs or device within 60 days of screening 15. Any myorelaxant medication including IT baclofen, taken by the subject in the last 14 days prior to screening (step 1) 16. Not stable under IT baclofen or per os myorelaxant medication for at least 30 days prior screening (step 2) 17. Not stable on all other chronic medications for ≥ 30 days prior to screening, including analgesics 18. Injection of BTX-A in striated muscle less than 3 months ago 19. Subject is currently using, and will continue to use for the next 14 days any of the following medications which are classified as Inhibitors of CYP 1A2 (e.g. diclofenac, diazepam, nicergoline, clomipramine, imipramine, fluvoxamine, phenacetin, theophylline, amitriptyline and quinolones) or Inducers of CYP 1A2 (e.g. rifampicin and omeprazole) 20. Ongoing pregnancy and women with childbearing potential not using any form of efficacious contraception during study and 3 months after the end of study. 21. Ongoing lactation and during 3 months after the end of study. 22. Known hypersensitivity to Riluzole |
| **EXPERIMENTAL DRUGS** | Riluzole capsules (25 mg and 50 mg) and related placebo will be distributed to investigating centers by the PUI (UEPRB) of AP-HM. |
| **EXPERIMENTAL TREATMENT** | Step1: Dose finding phase: Riluzole capsules (25 or 50 mg) will be administered in the four dose level groups (i.e. 25 mg bid; 50 mg bid; 75 mg bid; 100 mg bid). The number of capsules administered per dose will be 2, twice daily from Day 1 to Day 14:  -group 25 mg : 1 capsule of Riluzole 25 mg and 1 capsule of placebo  -group 50 mg : 1 capsule of Riluzole 50 mg and 1 capsule of placebo  -group 75 mg : 1 capsule of Riluzole 25 mg and 1 capsule of Riluzole 50 mg  -group 100 mg : 2 capsules of Riluzole 50 mg  Step2: Comparative phase: Riluzole capsules (25 or 50 mg) or Placebo will be administered at the selected dose, twice daily, from Day 1 to Day 14.  Patients who terminated Step 1 will be proposed to also participate to Step 2. |
| **COMPARATOR** | Placebo (step 2) |
| **Others research acts** | Biological sampling for biomarkers and genetics (optional) |
| **Additional risks of the research** | Drug-induced Hepatitis |
| **Visit schedule and assessments** | **Visit 1 (**Week-2):   - Informed consent prior to any procedures, - Screening assessments: - Score ASIA and grade AIS, - General data, - Vital signs, - Medical and surgery history, - Inclusion/exclusion criteria, - Systematic clinical examination, - 12-lead ECG, - Safety blood sampling and urinary pregnancy test (for female patients), - Biological sampling for biomarkers and genetics, - Rehabilitation (type and frequency), - Modified Ashworth scale on at least adductor muscles and/or triceps surae, - 0-10 NRS spasticity, - DN4 questionnaire (« Douleur neuropathique en 4 questions »), - VAS pain, Neuropathic Pain Symptom Inventory, - ISCIPDS, - Adverse events, - Concomitant treatments   **Only in Step 2**   - Bladder diary given (to be filled 1 day within 3 days before randomisation),   Duration of screening period: from Week -2 to Week 0.  **Visit 2** (Week 0 Day 1):   - Inclusion and exclusion criteria verification, - Vital signs, physical examination - Safety blood sampling. - PK analysis (PK1): PK samples at T0 (before drug intake) and T2h (post dose), - Modified Ashworth scale on at least adductor muscles and/or triceps surae, - 0-10 NRS spasticity score, - Penn Spasm frequency scale, - VAS pain, - Neuropathic Pain Symptom Inventory, - ISCIPDS, PGIC , - Personal therapeutic objectives determined at baseline (Adductor muscle spasticity...), - Randomization and 1^st^ study drug administration - Adverse events, concomitant treatments, - Study drug and 0-10 NRS diary given,   **Only in Step 2**   - Spinal Cord Injury Independence Measure (SCIM scale), - Bladder diary return - EMG before drug intake and at T2h post dose,   **Visit 3** (Day 4 from randomization) 72h post 1^st^ dose:   - PK analysis: PK samples (PK2) at T0 (before drug intake) and T2h (post dose, - Safety blood sampling (haematology and biochemistry) - Vital signs, physical exam - Drug and 0-10 NRS diary check, - Adverse events, concomitant treatments,   **Only in Step 2**   - Bladder diary given (to be filed 1 day within 3 days before next visit).   **Visit 4** (Week2 Day 14):   - vital signs, physical exam - Modified Ashworth scale on at least adductor muscles and/or triceps surae, - 0-10 NRS spasticity score, - Penn Spasm frequency scale, - VAS pain, - Neuropathic Pain Symptom Inventory, - ISCIPDS, PGIC , - Personal therapeutic objectives determined at baseline (Adductor muscle spasticity...), - 12-lead ECG, - PK analysis (PK3): PK samples at T0 (before drug intake) and T2h (post dose, - Safety blood sampling - Biological sampling for biomarkers, - Rehabilitation (type and frequency), - study drug return, compliance to treatment - adverse event, concomitant treatments, - drug and 0-10 NRS diary return   **Only in Step 2**   - Spinal Cord Injury Independence Measure (SCIM scale), - Bladder diary return - EMG before drug intake and at T2h post dose   **Visit 5 (Week 3 Day 21): follow up visit:**   - Vital signs, physical exam - Modified Ashworth scale on at least adductor muscles and/or triceps surae, - 0-10 NRS spasticity score - VAS pain, - Neuropathic Pain Symptom Inventory, - ISCIPDS, - Adverse events, concomitant treatments. |
| **NUMBER OF PATIENTS** | The characteristics of the sequential Bayesian method have shown that the inclusion of 30 patients (step 1) and 60 patients (step 2) is sufficient for this type of study. Stopping rules will be sequentially applied to eventually stop the inclusions before this number. |
| **NUMBER OF CENTERS** | 7 French national centers |
| **RESEARCH TIMETABLE** | - Enrolment period: 24 months (8 months + 16 months) - Duration of subject participation (screening + treatment + follow-up): 5 weeks (each Step)   Total study duration: 36 months |
| **Number of patient by center -by month** | 1 (Step 1)  0.4 (Step 2) |
| **STATISTIC ANALYSIS** | A Statistical Analysis Plan (SAP) containing detailed methods will be provided before the beginning of the study. Bayesians analysis will be performed by F-CRIN Platform (Dr. Corinne Alberti). Patients’ description, secondary analysis and safety analysis will be performed by the CIC-CPCET (Elisabeth Jouve). Analyses will be conducted in respect of international guidelines and recommendations for clinical trials (ICH, CONSORT) and following internal Standard Operating Procedures.  Quantitative variables will be described as medians [quartiles] or means (standard deviation) depending on the Gaussian distribution or not. Qualitative variables will be described as numbers (percentages). Inter-group comparisons will be conducted using parametric or non-parametric tests according to the nature and the distributions of the variables. Unless otherwise specified, statistical significance is defined as p<0.05. |
| **FUNDER** | PHRC |
| **DSMB** | Yes |
| **CLINICAL TRIAL REGISTRY** | Clinicaltrial.gov |

# Rationale of the research

## Hypothesis of research

This trial aims at (1) evaluating the minimal effective dose (MED) of Riluzole for the treatment of spasticity in patients following chronic Spinal Cord Injury (SCI), (2) assessing, in a phase 2 trial, the efficacy of Riluzole to improve spasticity and neuropathic pain versus placebo, (3) investigating a possible dose/effect relationship and (4) determining the safety and the efficacy of Riluzole in chronic SCI patients.

## Background

The causes of SCI are most often traumatic, [motor vehicle accidents](http://en.wikipedia.org/wiki/Motor_vehicle_accident) being the most common cause of SCIs, while other causes include [falls](http://en.wikipedia.org/wiki/Falling_(accident)), work-related accidents, sports injuries, and [penetrating trauma](http://en.wikipedia.org/wiki/Penetrating_trauma) such as stab or gunshot wounds (Ward, 2003). SCIs can also be of a non-traumatic origin, as in the case of cancer, infection, neurodegenerative diseases, intervertebral disc disease, and spinal cord vascular disease (Bogdanov, 2014).The American Spinal Injury Association (ASIA) first published an international classification of spinal cord injury that is widely used to document sensory and motor impairments following SCI (Maynard et al., 1990). Based on the ASIA impairment scale, the severity of the injury, also called "the completeness", is classified into five categories ranging between A and E score where A indicates a "complete" spinal cord injury where no motor or sensory function is preserved and E indicates "normal" where motor and sensory scores are normal. It is possible to have spinal cord injury and neurological deficits with completely normal motor and sensory scores (Maynard et al., 1990). Published results of global incidence for traumatic SCI vary from 9.2 to 246.0 cases per million inhabitants a year (van den Berg et al., 2010). The estimated incidence varied considerably according to the geographic region as follows: (i) the Americas: 20.7 to 83.0 per million inhabitants a year; (ii) Europe: 8.0 to 130.6; (iii) Asia and the Middle East: 14.6 to 246; and (iv) Oceania: 10.0 to 77.0 (van den Berg et al., 2010). The global prevalence varied from 236 to 1,298 per million inhabitants with an increasing trend over the last three decades.

Spasticity has been defined as an increase in muscle tone due to hyper excitability of the stretch reflex and is characterized by a velocity-dependent increase in tonic stretch reflexes (Lance, 1980; Sheean, 2002). A more clinically relevant definition which also includes clonus, spasms, and hyper reflexia is also used (Skold, 2000). Spasticity is a frequent complication in patients with injury of the central nervous system, and is a sign of damage to upper motor neurons, (Decq, 2003; Sheean, 2002). When the injury that leads to spasticity is acute, muscle tone is flaccid with hypo reflexia before the appearance of spasticity. The interval between injury and the appearance of spasticity varies from days to months according to the level of the lesion. In addition to increased muscle tone, the signs in spasticity include clonus, the clasp-knife phenomenon, hyper reflexia.

Previous studies have shown that 65–78% of sample populations of individuals with chronic SCI (more than 1 year post-injury) have symptoms of spasticity (Skold et al., 1999; Skold, 2000). Although unclear and strongly debated, it has been suggested that the ASIA classification of SCI (severity) and the level of injury may predict the likelihood of developing spasticity (Skold, 2000).

In some patients spasticity has some advantages as it substitutes for strength and thus facilitating transfer, standing and ambulation (Adams and Hicks, 2005). Light to moderate spasticity contributes to better circulation in the legs, thereby avoiding oedema. However, spasticity in chronic SCI is a chronic debilitating conditionwith many negative aspects influencing quality of life (QOL), including interference with functional mobility, hygiene and daily activities. Spasticity can limit the transition from bed to chair, comfortable sitting position, and also make the routine hygiene difficult to maintain. It may also cause painful spasms, fatigue, result in fracture, interfere with sleep, loss of range of motion (ROM) or increase the risk of pressure ulceres and contribute to infections, negative self image, complicating the role of the caretaker, and impeding rehabilitation efforts (Burchiel and Hsu, 2001; Jozefczyk, 2002; Kirshblum, 1999; Parziale et al., 1993; St George, 1993). Untreated spasticity can lead to permanent muscle contractures and ultimately to muscle shortening. Finally, severe spasticity could require surgical procedures with associated risks, burden and costs. To avoid such negative developments, treatment should start as soon as possible (Yelnik et al., 2009; Yelnik et al., 2010). Spasticity may be general, regional or localized and associated with tetra paresis, hemiparesis, para paresis or mono paresis. Currently, none of the available treatments can reduce efficiently and globally all symptoms associated with spasticity.

Multiple spinal mechanisms appear to be involved in the pathogenesis of spasticity after SCI (Ward, 2008). A change in the excitability of various supra-spinal inhibitory nerve pathways seems to be the main explanation. More recent research has shown a change in the excitability of both motor neurons and interneurons (Boulenguez et al., 2010; Furlan et al., 2013). Thus an increase in the ratio of excitatory-to-inhibitory inputs to motoneurons originating predominantly from spinal interneurons appears to be involved in the development of spasticity. The alteration of synaptic inhibition appears to play critical roles in the manifestation of spasticity after SCI, leading to increased alpha motor neuron excitability at the segmental cord level and subsequent increase in muscle tone (Boulenguez et al., 2010).

To assess spasticity, clinical, biomechanical, and neurophysiological approaches have been used. Clinical scales for the assessment of spasticity mainly concentrate on resistance to passive movement (Platz et al., 2005). Many of them are single item scales that can be used in different circumstances, that is, different joints and different underlying diseases. The Ashworth scale (AS)(Ashworth, 1964) or its’ modified version (MAS)(Bohannon and Smith, 1987) are the most commonly used clinical measurement methods for the assessment of tone. Both versions of the AS and MAS measure the resistance perceived by the rater when passively rotating a joint, which is scored on an ordinal scale. The perceived resistance to passive movement is a sum total of neural stretch reflex activity and non-neural viscoelastic properties of joint structures and soft tissues (Gorassini et al., 2004; Jayaraman et al., 2006; Vattanasilp et al., 2000). Moreover the AS and MAS take into account an immediate status of spasticity and might not reflect the overall spasticity over time.

The 0-10 NRS spasticity score is an alternative method used to evaluate spasticity. It has been already used for SCI patients as secondary criteria (Sativex trial and Ultramicronized PEA Normast) and has been validated for Multiple Sclerosis spasticity assessment (Farrar, 2008). The 0-10 NRS is an auto-evaluation by the patient and takes into account overall spasticity over the last 24 hours.

These 2 scales have in common that they depend on the perception of the examiner or patient, that differentiation between neural and non-neural contributions is not possible, and that the methodological quality of the scales is difficult to establish. For this reason patients are often evaluated using a combination of different scales/tests that consider both clinician and patient perception of spasticity changes.

Currently approved therapies against spasticity related to SCI include benzodiazepines, baclofen either orally or intrathecally administered, Tizanidine (Temporary Use Authorization in France), and Dantrolene sodium. Local intramuscular Botulinum Toxin A is also administered in case of focal spasticity.

Pain following SCI is present in approximately two-thirds of patients after SCI, with nearly one-third rating their pain as severe (Siddall et al., 1997). A taxonomy has been proposed by the Spinal cord Injury Pain Task Force of the International Association of the Study of Pain (Siddall and Loeser, 2001), classifying pain as neuropathic and nociceptive. Chronic pain (either nociceptive or neuropathic) may be associated with mood changes, sleep disturbance, fatigue and may have an impact on physical and social functioning.

Nociceptive pain activated by the somatic or visceral nociceptors, often in association with the trauma, is usually not related to a sensory or motor deficit.

In opposition, neuropathic pain following spinal cord injury has been defined as pain initiated or caused by a primary lesion or dysfunction of the nervous system (Merskey and Bogduk, 1986). The way that the patients usually describe this type of pain includes sharp, shooting, electric, burning, and stabbing. These syndromes comprise a complex combination of symptoms as sensory deficits, dysaesthesia, allodynia, hyperalgesia and paresthesia. The pain may be more or less persistent, fluctuating in time or even periodic. Chronic central neuropathic pain occurs in approximately 40% of patients with SCI (Siddall et al., 2003).

Standard diagnostic tools for pain are used, including the Visual Analogic Scale (VAS), the Numerical Rating Scale (NRS). Some diagnostic tools specifically developed for neuropathic pain have been validated recently, including the Leeds assessment of neuropathic symptoms and signs (LANSS), the neuropathic pain questionnaire (NPQ), the “douleur neuropathique en 4 questions” (DN4) (Bennett et al., 2005; Haanpaa et al., 2011), and the Neuropathic Pain Symptom Inventory (Bouhassira, 2004).

Finally, a SCI specific scale, the International SCI Pain basic data set (ISCIPDS) has been developed and validated by ISCOS (Widerström-Noga et al., 2008).

Currently therapies against neuropathic pain include tricyclic antidepressant, antiepileptic-medications (lamotrigine, pregabalin) and analgesics (tramadol). Baclofen and BTX-A are given to all eviate the nociceptive pain directly related to spasticity (Merskey and Bogduk, 1986). All these treatments currently in use lead to a reduction of 20-30% of pain sensation, which is not fully satisfactory (Siddall and Loeser, 2001).

**Therefore there is an impellent need to identify a new drug which can reduce both spasticity and neuropathic pain in SCI with relatively few side effects.**

Riluzole is the only disease-modifying drug approved for the treatment of amyotrophic lateral sclerosis (ALS), in which it has been demonstrated to extend survival and/or delay the use of mechanical ventilation. It is known to have several modes of action, including glutamate release inhibition and voltage-gated sodium channel blocking capabilities (Estevez et al., 1995), the latter being involved at all phases following SCI and in several symptoms:.

1) Early mechanisms involved in secondary axonal loss after SCI have been shown to be largely related to the deregulation of Na+ homeostasis, i.e. to the accumulation of intracellular Na+ through voltage-gated sodium channels (Agrawal and Fehlings, 1996; Stys et al., 1992).

2) The pathophysiological mechanisms of spasticity are diverse but alterations in intrinsic motoneuron properties have been postulated to play a central role (Bennett et al., 2001; Boulenguez and Vinay, 2009; Gorassini et al., 2009; Sadlaoud et al., 2010). In particular, the SCI predisposes motoneurons to express exuberant self-sustained plateau potentials contributing to uncontrollable muscle spasms (Eken et al., 1989). The persistent sodium current (*I*_NaP_), is one of the key conductance driving motoneuronal self-sustained plateau potentials through voltage-gated sodium channels (Bouhadfane et al., 2013). The increase of the *I*_NaP_ after SCI has been assumed to mediate abnormal tonic firing in motor units (Bennett et al., 2001; Gorassini et al., 2004; Harvey et al., 2006; Heckman et al., 2008; Li and Bennett, 2003; Powers and Rymer, 1988).

3) Interestingly it has been shown that after experimental SCI in rats, lumbar dorsal horn nociceptive neurons become hyper responsive linked to aberrant re-expression of voltage-gated sodium channel Nav1.3. The implication of this channel in hyper-responsiveness and pain-related behaviours after SCI was demonstrated by knock-down experiments (Hains et al., 2003; Waxman and Hains, 2006). *I*_NaP_ currents generated by Nav1.3 channels have been shown to be also amplified in nociceptive neurons of the dorsal horn after SCI (Lampert et al., 2006).

4) The density of Tetrodotoxin-sensitive (thus potentially Riluzole-sensible) sodium currents is increased in bladder afferent neurons of rat model of SCI (Yoshimura and de Groat, 1997), suggesting that *I*_NaP_ could be involved in bladder hyperactivity through voltage-gated Na+ channels.

It follows that voltage-sensitive Na+ channels are an important therapeutic target for the treatment of SCI.Based on these findings, Riluzole has attracted considerable interest as a potential neuroprotective, antispastic and antinociceptive drug for the treatment of SCI patients. The purpose of the present study is to assess the safety and efficacy of orally administered Riluzole on spasticity, neuropathic pain and bladder dysfunction, in patients with spinal cord injury in chronic phase.

## Summary of preclinical and clinical research

The pathophysiology of SCI involves a primary mechanical insult to the spinal cord and activation of a delayed secondary cascade of events, which ultimately causes progressive degeneration of the spinal cord. Two pathophysiological mechanisms explaining spasticity have been recently reviewed (Boulenguez and Vinay, 2009): 1. an hyperexcitability of motoneurons resulting from a change in their intrinsic electrophysiological properties, 2. an increase of synaptic excitatory inputs resulting from an impairment of inhibitory systems.

Under normal conditions motoneurons develop continued depolarizations which are due to Persistent calcium/sodium Inward Currents" PICs(Bennett et al., 2001). After SCI, in the absence of control by descending pathways, these PICs would be largely responsible for the hyperexcitability of motoneurons and for the onset of long term reflex activities and spasms (Boulenguez and Vinay, 2009). A recent work by the Vinay’s team showed that persistent sodium currents play a major role in the development of spasticity and other teams have shown that these currents might be involved in the development of neuropathic pain after SCI(Hama and Sagen, 2011; Lampert et al., 2006). This sodium current has the peculiarity of being blocked by Riluzole (Bouhadfane et al., 2013; Brocard et al., 2013; Cifra et al., 2013; Tazerart et al., 2007; Tazerart et al., 2008). In this regard Riluzole has been tested in adult female rats of Wistar strain with thoracic section (complete spinal cord injury atT8-T9 level). Preclinical results showed that the systemic administration of Riluzole (IP single dose 8mg/kg or repeated doses of: 4mg/kg twice a day for two weeks) was able to significantly reduce spasticity (Brocard et al. unpublished data). However a lower dailydoseof2mg/kg(1mg/kg twice daily)had no effect. In addition, this study demonstrated that Riluzole has no function in the modulation of the glutamatergic neurotransmission at the doses used, which suggests a different mechanism from that proposed for Riluzole in the treatment of Amyotrophic Lateral Sclerosis.

In order to further understand the mechanisms of action of Riluzole on spasticity, L Vinay and his team (C Brocard, P Boulenguez, V Plantier, S Liabeuf, Institut Neurosciences Timone, Marseille) performed recordings of the motoneurons activity in vitro on a whole isolated spinal cord preparation from new born rats (Wistar strain) that underwent SCI (T8-T9) at birth. The results showed that in these pathological conditions, by its direct action on sodium channels, Riluzole was able to reduce the excitability of the motoneurons (Brocard et al., unpublished data). Besides, recent studies show that single systemic injection of Riluzole (8mg/kg) after spinal cord contusion increases the threshold of response to mechanical stimulation in a rat model of SCI-induced neuropathic pain (Hama and Sagen, 2011). Riluzole (8mg/kg daily for 7 weeks) attenuates neuropathic pain and enhances functional recovery in a rodent model of cervical spondylotic myelopathy (Moon et al., 2014). Riluzole delayed the onset of tactile hypersensitivity, slowed the development of thermal hypersensitivity and partially reversed established pain behavior following avulsion of the fifth lumbar spinal root (Chew et al., 2014). Administration of a single dose of riluzole (3 mg/kg IP) at Day 1 after a painful nerve root injury resulted in immediate resolution of mechanical allodynia and thermal hyperalgesia. This effect was maintained at Day 7 (Nicholson et al., 2014).

**Taken together, these results suggest that treatment with Riluzole could provide patients with spinal cord injury with benefits on both spasticity and neuropathic pain consecutive to trauma.**

Recent development has started in the US for acute Spinal Cord Injury on the basis of potential neuroprotective activity of Riluzole. A phase Ib has been completed (NCT00876889) and a Phase II is in progress (NCT01597518/ RISCIS) (Nagoshi et al., 2015). In the phase Ib study (Chow et al., 2012; Grossman et al., 2013), it has been shown that the Riluzole PK was linear but not stationary (plasma concentration day 14 < day 3), that the inter-individual dispersion was high. Also, the PK data reported for ALS, SMA and SCI suggest a possible difference related to the condition. Vegetative changes in acute SCI and/or PK interaction due to CYP1A2 activity changes might explain part of this variability. Also, no concentration/response or dose/response relationship for Riluzole has been evidenced yet and establishing PK/PD and K/PD relationships for Riluzole in patients with SCI remains an unmet clinical need.

Due to PK variability and absence of demonstrated dose or concentration/effect relationship, a dose ranging study is required to achieve confidence as regards to exposure and target engagement.

Interestingly Theiss et al. (2011) showed that a single dose of Riluzole (50 mg) induces significant change in long latency reflexes in chronic SCI patients suggesting that electromyography should evidence target engagement and pertinent mechanism of action.

**Originality and innovative aspects**

This is the first clinical study worldwide of the efficacy of Riluzole on spasticity and pain in chronic SCI. We will address the question of PK/PD relationships of Riluzole (in plasma). Using experimental medicine approaches, we will study the acute effect of Riluzole on electrophysiological parameters. Therefore we will fulfill the development criteria as defined by Morgan, 2012. In this rare condition, we will use a 2 steps Adaptive Bayesian design in order to minimize the number of patients to be included. This innovative multimodal and multidisciplinary approach is possible thanks to the full involvement of complementary facilities related to FCRIN platform and network (Partners, OrphanDev), Assistance Publique - Hôpitaux de Marseille (involved as sponsor and through Pharmacology, Neurology, Pharmacy and Physical Medicine and Readaptation Departments), UMR CNRS-AMU Institute of Neurosciences Timone, as well as French reference Physical Medicine and Readaptation Departments (Marseille, Nantes, Limoges, Montpellier) (see Annexes).In the 4 urban areas (2.55 million inhabitants), the number of patients with SCI is estimated to be 15700 with an annual number of new patients of 50 (estimation from Schema Regional Orientation Santé 2007-2012).

## Study population

Male and female patients with stable chronic SCI and spasticity will be included in the study.

## Experimental treatment

Riluzole, a benzothiazole Na+channel blocker, also known to inhibit presynaptic Ca2+-dependent glutamate release (Wang et al., 2004), is the only drug approved by FDA and EMA to prolong life expectancy or delay the use of mechanical ventilation in patient suffering from amyotrophic lateral sclerosis, a progressive neurodegenerative disorder characterized by motor neuron and corticospinal tract degeneration (Bensimon et al., 1994; Lacomblez et al., 1996). Riluzole is on the market under the trade mark of Rilutek®, Sanofi-Aventis being the Market Authorization holder. Another Market Authorization has been obtained on 19/11/2013 (5mg/ml oral suspension) by Italpharmaco (Spain). The approved regimen in ALS is fixed oral doses of 50 mg twice daily. There are also potential merits of riluzole, as an Na+ channel blocker, to offer neuroprotective activity in primary immediate (≤ 2 hours) and early acute (≤ 48 hours) injury phases of SCI. Indeed, studies have demonstrated that riluzole is neuroprotective and promotes functional neurological recovery in various species of animal models of brain and spinal cord ischemic and traumatic injury (Ates et al., 2007; Heurteaux et al., 2006; Lang-Lazdunski et al., 1999; Schwartz and Fehlings, 2001; Wu et al., 2013). A clinical phase II/III multicenter, placebo-controlled, randomized, double blind trial (NCT01597518), is currently ongoing with the objective to evaluate efficacy as neuroprotective agent and safety of Riluzole in the treatment of patients with acute SCI. The primary endpoint is International standards for neurological classification of SCI (ISNCSCI) examination motor score after 180 days.

For Spinal Cord Injury, Orphan Drug Designation (EU/3/14/1401, 16 December 2014) has been granted for riluzole by our group

(<http://www.ema.europa.eu/ema/index.jsp?curl=pages/medicines/human/orphans/2015/02/human_orphan_001491.jsp&mid=WC0b01ac058001d12b>).

This designation has been made according to prevalence of SCI in the EU and the medical plausibility for riluzole to be active on spasticity related to SCI.

## Drug dosage, route, duration of experimental treatment.

The study will be conducted in two steps in order to first identify the Minimal Effective Dose (MED) of riluzole, and second to confirm the antispastic effect of riluzole in the subsequent comparative study.

**Step 1:**

The four dose levels were chosen based on previous studies performed on ALS and acute SCI patients treated with Riluzole at standard regimen (Chow et al., 2012). Four dose levels are planned for this study: 25 mg BID, 50 mg BID; 75 mg (BID), 100 mg (BID). Then, daily doses will be 50 mg, 100 mg, 150mg or 200 mg. Dose level assignment will be determined by FCRIN platform (*see 4.2.1)*.

Patients will take the treatment from Day 1 to Day 14, orally, BID, before meals since co-administration of the drug with food can reduce absorption up to 20% (Fehlings et al., 2012)

The one week follow-up period (between V4 (Day 14) and V5 (Day 21)) will allow the elimination of riluzole between Step 1 and Step 2.

**Step2:**

The resulting ME dose will be used for patients’ administration in the following phase IIb, comparative (Riluzole versus placebo), randomized, double blind trial. Patients included in the active group (group 1) will take Riluzole orally, BID, fasting, from Day 1 to Day 14 at the established ME dose. Same administration schedule will apply to the placebo group patients (group 2).

**Duration of Treatment:**

The efficacy of Riluzole on neurophysiological mechanisms of spasticity is expected to be immediate (Theiss et al., 2011). However spasticity is a symptom subject to fluctuation in particular when a new treatment is introduced, and a period of 2 weeks for stabilization is usually required. Thus two weeks of drug intake are long enough to measure an improvement of spasticity.

## Justification of primary endpoint

This Phase 1b/2b study will determine the efficacy of Riluzole in improving spasticity in chronic SCI condition. Now, the clinical evaluation of spasticity remains difficult. Although the Ashworth scale or its modified version are the ones commonly used, their sensitivity to change and their clinical relevance is questioned by clinicians and health authorities. The 0-10 NRS score is an alternative method used to evaluate spasticity. It has been already used for SCI patients as secondary criteria (Sativex trial and Ultramicronized PEA Normast trial). In a recent Market Authorization (cannabinoid acting drug for MS spasticity), 0–10 NRS spasticity score has been adopted as a relevant endpoint by EMA. It has been validated for Multiple Sclerosis spasticity assessment (Farrar et al., 2008). Data from a total of 189 patients with MS (114 women, 75 men; mean age, 49.1 years) show that the test-retest reliability analysis found an interclass correlation coefficient of 0.83 (P < 0.001) between 2 measures of the 0–10 NRS spasticity scores recorded over a 7- to 14-day period before randomization. A significant correlation was found between change on 0–10 NRS and change in the Spasm Frequency Scale (r = 0.63; P < 0.001), and a moderate correlation was found between the change on 0–10 NRS and the Patient Global Impression of Change (r = 0.47; P < 0.001). A reduction of ≈30% in the spasticity 0–10 NRS score best represented the Clinically Important Difference (CID) and a change of 18% the Minimal CID (Farrar, 2008). In a recent trial, 74% of patients randomised to Sativex achieved an improvement of ≥30% from baseline by the end of the study (Novotna et al., 2011).

In this study, these two scales will be used in order to have a better evaluation and a higher sensitivity to spasticity changes before and after the treatment period, as assessed either by the patient (NRS) or the clinician (MAS).The primary endpoint will be the improvement of Modified Ashworth score better than 1 point, or 11 points Numerical Rating Scale (0-10 NRS) spasticity score better than 20% between Week 0 and Week 2.

## Summary of beneficial effects and risks for the patients during the protocol

Risks: Riluzole is the only disease-modifying drug approved for the treatment of amyotrophic lateral sclerosis (ALS), in which it has been demonstrated to extend survival. In the last decade Riluzole has attracted considerable interest as a potential neuroprotective, antispastic and anti-nociceptive drug for the treatment of SCI patients (see 2.2 and 2.3 sections). Results of previous studies in ALS patients showed that the overall tolerability of riluzole is good (Bensimon and Doble, 2004) and the drug can be used in all patients except those with elevated transaminase levels or active liver disease. The most frequently encountered adverse events (AEs), that appear to be attributed to riluzole, are asthenia, gastrointestinal disorders, nausea and dizziness that were more frequent in the 200-mg dose. These same AEs, albeit at a lower frequency, are also reported in Phase IV observational studies and in pharmacovigilance surveys. No unexpected AE clearly related to riluzole has emerged. The most important potential safety issue with riluzole is hepatic impact with elevations of transaminases. Serum alanine aminotransferase levels more than three times the upper limit of normal are observed in 10 – 15% of patients. Aminotransferase elevations were more frequent in the 100 and 200mg groups than in control group and were reversible after treatment discontinuation. Therefore, Riluzole should be prescribed with care in patients with a history of abnormal liver function, or in patients with slightly elevated serum transaminases (ALT/SGPT; AST/SGOT up to 3 times the upper limit of the normal range (ULN)), bilirubin and/or gamma-glutamyl transferase (GGT) levels. Baseline elevations of several liver function tests (especially elevated bilirubin) should preclude the use of riluzole. For this reason, ALT should be measured every month during the first 3 months of treatment, every 3 months during the remainder of the first year, and periodically thereafter. Riluzole should be discontinued if the ALT levels increase to 5 times the ULN.

Not as commonly reported, but still very serious, is neutropenia, and physicians should be vigilant towards this risk.

Benefits: Riluzole has shown efficacy in the improvement of spasticity and increment of the mechanical pain threshold after SCI in preclinical studies. One preliminary study has confirmed the positive effects of riluzole on spasticity related electrophysiological changes in 7 patients with SCI and spasticity (Theiss et al., 2011).

# objectives

## Main objective

This Adaptive design clinical study comprises two steps:

**Step 1**: To determine the Minimum Effective Dose (MED) of Riluzole, among the four doses of the panel, that improves spasticity in patients with chronic SCI

**Step 2**: To demonstrate, the efficacy of the MED in improving spasticity versus placebo, in patients with chronic SCI.

## Secondary objectives

- To determine the safety of Riluzole in SCI patients
- To determine the pharmacokinetic (PK) of Riluzole in SCI patients
- To determine the pain relieving effect of Riluzole in SCI patients
- To determine the effects of Riluzole on activities and participation in SCI patients
- To determine the effects of Riluzole on bladder dysfunction

# Design of the research

## Main and secondary end points

### Primary endpoint

The primary criterion is binary (success or failure). Success will be defined as follows: “Improvement of Modified Ashworth Scale score better than 1 point or 0-10 NRS spasticity score better than 20% between Week 0 and Week 2.

### Secondary endpoints

-**Safety**: To evaluate the safety and the side effects of riluzole after two weeks treatment.

**-Pharmacokinetics**: Blood sampling time points will be estimated using Limited Sampling Strategy (according to the selected galenic form and sex). Individual parameters will be calculated using standard compartmental approaches. In particular, exposure parameters (i.e., Cmax, Cthrough, AUC) will be evaluated.

**-Concentration/effect relationship**

**-Efficacy**: Patient Global Impression of Change, 0-10 NRS score, Modified Ashworth score, Penn Spasm frequency scale

**-Pain**: VAS, Neuropathic Pain Symptom Inventory, ISCIPDS

**- Activities and participation**:

- Personal therapeutic objectives determined at baseline (Adductor muscle spasticity...)

- Spinal Cord Injury Independence Measure (SCIM scale) (only for Step 2)

**-Electrophysiology**: H reflex, F, surface EMG at Tmax ((only for Step 2)

- **Bladder dysfunction**: bladder diary(only for Step 2)

## Study design

### Experimental design

**Step 1**: A double-blinded trial will be designed using a continual reassessment method based on Bayesian inference. The principle of this method is to identify the adequate drug dosage to obtain a level of efficacy as close as possible to a predetermined target level of efficacy in the population. Four dosages of Riluzole will be tested from 50 to 200 mg/day (50, 100, 150 and 200 mg/day) and a target probability of success of 75% will be chosen. Clinicians have attributed initial guesses of success rate to each dosage according to their clinical experience and information available in the existing literature. The following initial guesses are: dose of 50 mg/day: 10% of success rate; 100 mg/day: 50%; 150 mg/day: 75%; 200 mg/day: 85%.

At the first stage, the starting dose of Riluzole will be selected by FCRIN platform. Each response to the treatment will be communicated to the biostatistician of FCRIN platform who will perform the Bayesian analysis. Every two inclusions and in accordance with the outcomes assessed at the end of the 2- week treatment, the success rate will be updated. The dose for the two next patients will be allocated according to the results of the statistical analysis that will be undertaken. Doses will be reassessed every two patients. Each patient will be treated at the dose set for his cohort. A maximum of 30 patients will participate at this stage.

**Step 2**: A phase IIb, placebo-controlled, parallel-group, multicentre, randomised, double-blind trial will compare the effects of treatment with riluzole (daily dose defined during step1) versus placebo in patients with chronic SCI:

-group 1 with riluzole treatment BID per os

-group 2 with placebo BID per os

The statistical analysis will use the Bayesian inference mainly because the maximum number of patients that could be recruited is 60. This does not allow answering the clinical question in the frequentist framework.

### Number of centers

7 French national centers (list in Annexe)

### Randomisation

Step1: Dose level assignment will be determined by FCRIN platform.

Step 2: Randomization will be performed as block randomisation stratified for center. Patients will be randomized to either riluzole (groupe 1) or placebo (groupe 2).

At the end of the screening visit, the patients will be randomized to one of the treatment groups.

### Blind modalities

The double-blind will be maintained for patients and investigators. FCRIN platform will only inform the pharmacist (Timone Hospital Marseille) who will prepare the experimental treatment of the dose, in order to maintain the blinding of the investigator and the patient.

### Unblinding procedures

Unblinding is the process by which the allocation code is broken so that the investigator, clinical staff and/or the trial statistician becomes aware of the intervention for a person participating in a trial.

It is important to ensure that participating people are not unblinded unnecessarily and the study results are not compromised. Equally, unblinding should occur in a responsive manner when it is clinically indicated. Unblinding is required:

- To make clinical treatment decisions or when an unexpected serious adverse event occurs and the intervention must be made known. If required, the investigator may request unblinding, with the coordinator's approval, from the sponsor,
- At the request of the Data Safety Monitoring Board,
- During an unmasked analysis in accordance with the study analysis plan.
- Only the FCRIN platform and the clinical trial pharmacy team of the Timone hospital will be unblended and will manage unblinding procedures. Unblinding will be possible 24/24; 7/7 by contacting the pharmacy of the Timone hospital.

### Validation procedures

During the study, each CRF will be validated (Primary endpoint and drug observance to be between 90 and 105% according to WHO recommendation) before being used for subsequent dose assignment.

# Study description

## Overall study description

The study consists of an enrollment period of 24 months. During the screening visit, investigator will explain the study to patients and will supply information letter in order to allow the patient to have reflexion time.

**Step1**: Up to 30 patients with chronic SCI will undergo a screening period (V1) with a maximum duration of 2 weeks and then will be assigned to one riluzole treatment dose level (V2)

**Step 2**: Up to 60 patients with chronic SCI will undergo a screening period (V1) with a maximum duration of 2 weeks and then they will be randomized to one of the two groups (v2): riluzole twice daily or placebo twice daily for a 2 weeks period.

For Step 1 and Step 2, three additional visits are planned: at Day 4 after the beginning of treatment for PK analysis (V3), at the end of the 2 weeks treatment (V4) and one week after withdrawal of study treatment (V5) for a total participation period of approximately 5 weeks. Patients who terminated Step 1 will be offered to participate to Step 2.

**Treatment assignment**

**Day 1**

**End of**

**Treatment**

**Day 14**

**End of study**

**Day 21**

**Pkvisit**

**Day 4**

1 week FU

2 weeks treatment

-2 weeks screening

**V2 V3**

**V1**

**V 4**

**V5**

## Study assessments and procedures

Patients, clinicians and evaluators will be blinded to treatment assignment. Outcome measures will include the following:

### Primary outcome measure (at 2 weeks):

The primary criterion is binary (success or failure). Success will be defined as follows: “Improvement of Modified Ashworth Scale score better than 1 point or 0-10 NRS spasticity score better than 20% between Week 0 and Week 2”

The 0-10 NRS is a patient reported outcome measure (PROM) which has been shown to be a valid and reliable tool in the assessment of spasticity, with a moderate to high level of correlation with other clinician-rated instruments used to assess spasticity, such as the Modified Ashworth Scale (Farrar, 2008). 0-10 NRS is measured according to the level of spasticity over the preceding 24 hours on a 0–10 NRS (0 = “no spasticity” to 10 = “worst possible spasticity”). 0-10 NRS score will be assessed by the patient at screening, randomization, end of treatment and follow up visits and every 24 hours of the treatment period. The patient will be asked to report the scores in the “drug and 0-10 NRS” diary.

The Ashworth scale or its’ modified version are the most commonly used clinical measurement methods for the assessment of tone. Both versions of Ashworth scale measure the resistance perceived by the rater when passively rotating a joint. The modified version of Ashworth scale (MAS, Bohannon and Smith, 1987) allows better assessment of spasticity since differentiation of patients at an important threshold of spasticity is possible (0, 1, 1+, 2, 3, 4 versus 0, 1, 2, 3, 4 for the original Ashworth scale (Bohannon and Smith, 1987; Farrar et al., 2008). However the 1+ does not allow quantification, thus the version of MAS from 0 to 5, which is currently used and validated, will be used (Haute Autorité de Santé, 2006). MAS score will be assessed by the clinician on at least adductor muscles and/or triceps surae muscles at screening (V1), randomization (V2), end of treatment (V4) and follow-up (V5) visits.

### Secondary outcome measure (at 2 weeks):

- Penn Spasms Frequency Scale:

This scale is a 2 component self-report measure of the frequency of reported muscle spasms which is commonly used to quantify spasticity developed to augment clinical ratings of spasticity and provide a more comprehensive understanding of an individual’s spasticity status. The first component is a 5 points scale assessing the frequency with which spasms occur ranging from “0 = No spasms” to “4 = Spontaneous spasms occurring more than ten times per hour”. The second component is a 3 points scale assessing the severity of spasms ranging from “1=Mild” to “3=Severe”. The second component is not answered if the person indicates they have no spasms in part 1.

- Patient Global Impression of Change (PGIC):

This scale is a 7-points categorical scale reported by the patient from 1 (very much improved) to 7 (very much worse). The patient assesses the overall change in his/her condition since entry into the study.

- Visual Analog Scale for Pain:

This scale is a 11-points ordinal scale reported by the patient from 0 (no pain) to 10 (worst possible pain).

- Neuropathic Pain Symptom Inventory (NPSI):

This questionnaire allows the clinician to classify types of neuropathic pain and to evaluate the change induced by pharmacological treatment.

- International Spinal Cord Injury Basic Data Set (ISCIPDS):

This questionnaire applies to all pain interferences (day-to-day activities, overall mood, ability to get a good night’s sleep), the number of different pain problems and the description of the worst 3 ([http://www.iscos.org.uk/sitefiles/2013%2006%2011_International%20SCI%20Pain%20Basic%20Data%20Set_Version%202%200.pdf](http://www.iscos.org.uk/sitefiles/2013 06 11_International SCI Pain Basic Data Set_Version 2 0.pdf))

- Spinal Cord Injury Independence Measure (SCIM scale):

The SCIM addresses three specific areas of function in patients with SCI. It has been validated in this population (Catz and Itzkovich, 2007) and includes assessment of self-care (feeding, grooming, bathing, and dressing), respiration and sphincter management, and a patient’s mobility abilities (bed and transfers and indoors/outdoors). The patient is scored by the clinician. This outcome will be analyzed only in Step 2.

- Personal therapeutic objectives:

Personal objectives will be determined at baseline (e.g. Adductor muscle spasticity...) by the clinician together with the patient.

- Electrophysiology: Nerve conduction study and Electromyography

Nerve conduction studies will be performed using standard techniques at a temperature of 32°C using customized EMG machine available in participant centers. The studies will include tibial motor nerves. The tibial nerve will be stimulated at the ankle and popliteal fossa, and compound muscle action potential (M-Wave) will be recorded over the abductor hallucis. Measurements included negative peak duration, baseline-to-peak amplitude, mean F-wave amplitude and soleus H reflex. The maximal H-reflex and M-wave responses allow for comparison of H-reflex maximum amplitude and M-wave maximum amplitude (Hmax/Mmax) ratios across patients during different recording conditions.

Surface EMG will be performed in experienced centers only. Patients will be seated in an adjustable chair with their test foot securely strapped to an instrumented footplate attached to a mechanical fixture. Surface electrodes will be attached to the skin above the muscle to measure the activity in the right and left tibialis anterior (TA) and soleus (SOL). The EMG signal will be amplified and filtered (3Hz–3kHz, GRASS P511K). Electrophysiological signals will be recorded and stored on a computer using the CED 1401+ device and the Spike 2-6 software program (CED, Cambridge, UK). EMG activity will be sampled at rates of 5 kHz. TA and SOL EMG responses will be normalized to the area of the maximal M-waves. Electrophysiology examinations will be performed at T0 (before drug intake) and T2h (2h post dose) on Day 1and Day 14.

This outcome will be analyzed only in Step 2

- Riluzole Pharmacokinetics:

Riluzole concentrations in plasma will be quantified using a high performance liquid chromatography (HPLC) assay with U.V. detection, adapted from Chow et al.)2012), after a liquid-liquid extraction step from a 200µl plasma sample (see section 8).

- Safety and tolerability,

As assessed by the occurrence of adverse events and serious adverse events;

- Laboratory safety parameters:

Laboratory analysis will all be performed locally (cf section 10.1).

- Concomitant treatments:

All drugs currently used for chronic SCI patients will be recorded with: name, dose, regimen, dates of start and stop (cf section 7.2)

- Bladder diary:

The patient will report on a diary the frequency of urinary probing and urinary leakages during 1 day in the 3 days preceding visit V2 (randomization V2 Day 1) and in the 3 days preceding the visit 4 (end of treatment V4 Day 14)

- Other measures:

DN4 questionnaire: this questionnaire allows the clinician to discriminate nociceptive from neuropathic pain. It will be performed at V1 (screening).

## Visits in clinical department

The study schedule will be the same in step 1 and in step 2 and it includes the following visits and clinical examinations:

### Visit 1: screening visit (V1) (in the 2 weeks preceeding randomisation)

Before any research examination or acts, investigator obtains from the patient informed and written consent to participate in the trial. During the screening visit in clinical department the patient will have the opportunity to ask questions before signature of the informed consent and then informed consent will be obtained.

Then, study procedures as outlined will be proceeded:

- ASIA impairment scale, AIS

- General data,

- Vital signs (Systolic and diastolic blood pressure, heart rate)

- Medical and surgery history, including tobacco and caffeine use (since it is an inducer of CYP1A2)

- Inclusion/exclusion criteria,

- Systematic clinical examination,

- 12-lead ECG

- Safety blood sampling (haematology, biochemistry, serologies hepatitis B and C, HIV; urinalysis) cf 10.1,

- Urinary pregnancy test (for female patients),

- Biomarkers and genetics samples (optional),

- Biological sampling for baclofene determination,

- Adverse events,

- Rehabilitation (type and frequency),

- Modified Ashworth score on at least adductor muscles and/or triceps surae,

- 0-10 NRS spasticity,

- Bladder diary given (to be filled in within 3 days before randomisation) (only for patients in Step 2),

- DN4

- VAS pain,

- Neuropathic Pain Symptom Inventory,

- ISCIPDS

- Concomitant treatments

### Visit 2: Day 1randomisation visit (V2)

-Inclusion and exclusion criteria verification,

- Vital signs, physical exam

-randomisation,

- study drug dispensation,

- 0-10 NRS spasticity score,

- Drug and 0-10 NRS diary given,

- Modified Ashworth score on at least adductor muscles and/or triceps surae,

- Penn Spasm frequency scale,

- VAS pain,

- Neuropathic Pain Symptom Inventory,

- ISCIPDS

- Personal therapeutic objectives determined at baseline (e.g. Adductor muscle spasticity...),

- PK analysis (PK1): PK samples at T0 (before drug intake) and T2h (post dose,

- Safety blood sampling (haematology, biochemistry, urinalysis)

- Adverse events,

- Concomitant treatments.

- Spinal Cord Injury Independence Measure (SCIM scale) (only for patients in Step 2),

- EMG before drug intake and at T2h post dose (only for patients in Step 2),

### Visit 3: Day 4 PK Visit (V3)

- PK analysis: PK samples (PK2) at T0 (before drug intake) and T2h (post dose),

-Safety blood sampling (haematology and biochemistry)

- Vital signs,

- Drug diary check for compliance

- 0-10 NRS diary check

- Adverse events.

### Visit 4: Day 14 Efficacy Visit (V4)

- Vital signs, physical exam

- 0-10 NRS spasticity score,

- 0-10 NRS diary return

- Modified Ashworth score on at least adductor muscles and/or triceps surae,

- Penn Spasm frequency scale,

- Patient Global Impression of Change,

- VAS pain,

- Neuropathic Pain Symptom Inventory,

- ISCIPDS

- Spinal Cord Injury Independence Measure (SCIM scale),

- Personal therapeutic objectives determined at baseline (e.g. Adductor muscle spasticity...),

- 12-lead ECG,

- PK analysis (PK3): PK samples at T0 (before drug intake) and T2h (post dose),

- Safety blood sampling (haematology, biochemistry, urinalysis)

- Biological sampling for biomarkers (optional),

- Rehabilitation (type and frequency),

- Drug return

- Compliance to treatment (drug diary check and accountability),

- Adverse event,

- Concomitant treatments

- EMG at T0 (pre dose) and T2h (post dose)(only for patients in Step 2)

- Bladder Diary return (to be filled in 1 day within 3 days before the visit) ((only for patients in Step 2)

### Visit 5:follow-up visit (V5 Day 21)

- Vital signs, physical exam

- 0-10 NRS spasticity score,

- Modified Ashworth Scale on at least adductor muscles and/or triceps surae,

- VAS pain,

- Neuropathic Pain Symptom Inventory,

- ISCIPDS

- Concomitant treatments

- Adverse events.

### Chronological synopsis(for the 2 steps)

| Visits | v1 | v2 | V3 | V4 | V5 |
| --- | --- | --- | --- | --- | --- |
|  | Screening | Randomisation | PK | Efficacy | Follow Up |
|  | In the 2 weeks before Day 1 | Day 1 Week 0 | Day 4 | Day 14 Week 2 | Day 21 |
| Informed Consent signature | X |  |  |  |  |
| Inclusion/non inclusion criteria | X | X |  |  |  |
| Send fax/email for inclusion | X |  |  |  |  |
| Previous medical surgical history | X |  |  |  |  |
| Demographics | X |  |  |  |  |
| Vital signs, clinical and  neurological examination | X | X | X | X | X |
| Safety Blood Sampling | X | X | X | X |  |
| Biological sampling for biomarkers and genetics | X |  |  | X** |  |
| Randomisation |  | X |  |  |  |
| Bladder diary given and return***** | X | X | X | X |  |
| 0-10 NRS Spasticity | X | X |  | X | X |
| Modified Ashworth scale | X | X |  | X | X |
| SCIM***** |  | X |  | X |  |
| Penn Spasm frequency scale |  | X |  | X |  |
| PGIC |  | X |  | X |  |
| Personal therapeutic objectives |  | X |  | X |  |
| 12-lead ECG | X |  |  | X |  |
| PK analysis |  | X | X | X |  |
| EMG***** |  | X |  | X |  |
| DN4 | X |  |  |  |  |
| Neuropathic Pain Symptom Inventory | X | X |  | X | X |
| ISCIPDS | X | X |  | X | X |
| VAS pain | X | X |  | X | X |
| Investigational drug given and return |  | X |  | X |  |
| Concomitant treatments | X | X |  | X | X |
| Adverse events | X | X | X | X | X |
| Compliance to treatment |  |  |  | X |  |
| Physical rehabilitation description | X |  |  | X |  |
| Diary (Drug and 0-10 NRS) given, check, return |  | X | X | X |  |

***Only for patients included in Step 2**

****Only biomarkers at V4**

## Rules for stopping treatment or protocol

Each patient is informed that he/she can withdraw from this study at any time. He/she is asked to notify the investigating physician.

A patient can withdraw from the study for the following reasons:

- Specific request of the patient

- Serious adverse event (serious morbidity, hospitalisation, death)

- Intolerance

- Inefficacy

- Withdrawal of consent

- Non-compliant selection criteria

- Lost-to-follow-up

- Investigator decision

For each drop out, the following medical data should be collected insofar as possible:

The reason for dropout, the nature of the event and the clinical course will be recorded on the 'final assessment' CRF (completed during the final dropout visit).

Temporary or definitive cessations of treatment will be recorded of events in the CRF.

# Selection criteria

The trial will include up to 30 patients (Step 1) and up to 60 patients (Step 2) with chronic SCI, fulfilling the following inclusion and exclusion criteria. Patients who participated to Step 1 might be enrolled to Step 2.

## Inclusion criteria

1. Chronic traumatic SCI defined as:

At least a 12-month history of:

- - 1. C4-T12 traumatic SCI
    2. Complete and incomplete (AIS A,B,C,D)
    3. With Spasticity (5>MAS>1 on at least adductor muscles and/or triceps surae muscles and NRS ≥ 4)

1. Male or Female
2. Aged 18 to 65 years at the time of screening
3. Judged by site investigator to be able to comply with evaluations at baseline and throughout the study
4. Last injection of BTX-A in striated muscle more than 3 months ago and patients must have returned to their level of spasticity before botulinum toxin(BTX-A) injection.
5. Last intrathecal (IT) injection of baclofen or per os administration of any myorelaxant should be more than 14 days ago (Step 1)
6. The dose of myorelaxant or Baclofen should be stable for ≥ 30 days prior to screening and kept at stable daily dose until the end of the protocol (Step 2).
7. Stable on all other chronic medications for ≥ 30 days prior to screening, including analgesics
8. Stable on rehabilitation (methods and frequency) for ≥ 15 days prior to screening
9. Written informed consent provided by subject

## Exclusion criteria

1. Spinal cord injury of less than 12 months,
2. Associated Brain lesion that might be the cause of spasticity,
3. MAS≤1 or =5on at least adductor muscles and/or triceps surae muscles or NRS < 4
4. Presence of urinary infection, fever, pressure ulcer or other spasticity-aggravating factors.
5. Presence of other significant neurological or mental disorder or other illness, which would preclude accurate evaluation,
6. Recent history (less than 1 year) of chemical substance dependency or significant psychosocial disturbance,
7. Insufficient fluency in local language to complete neuropsychological, global and spasticity assessments
8. Active liver disease or clinical jaundice
9. Active malignancy or history of invasive malignancy within the last five years
10. Neutropenia clinically significant according to the investigator, Liver enzymes (ALT/SGPT or AST/SGOT) 2 times the upper limit of normal (ULN) at screening visit, Baseline elevations of several liver function tests (especially elevated bilirubin) clinically significant according to the investigator.
11. AIDS or AIDS-related complex,
12. The systolic blood pressure measurement is > 190 or < 85 mm Hg and/or the diastolic blood pressure measurement is > 105 or < 50 mm Hg at screening.
13. The ECG is abnormal at screening and judged to be clinically significant by the site investigator.
14. Treatment with any investigational drugs or device within 60 days of screening
15. Any myorelaxant medication including IT baclofen, taken by the subject in the last 14 days prior to screening (step 1)
16. Not stable under IT baclofen or per os myorelaxant medication for at least 30 days prior screening (step 2)
17. Not stable on all other chronic medications for ≥ 30 days prior to screening, including analgesics
18. Injection of BTX-A in striated muscle less than 3 months ago (BTX-A injection in detrusor is allowed)
19. Subject is currently using, and will continue to use for the next 14 days any of the following medications which are classified as Inhibitors of CYP 1A2 (e.g.diclofenac, diazepam, nicergoline, clomipramine, imipramine, fluvoxamine, phenacetin, theophylline, amitriptyline and quinolones) or Inducers of CYP 1A2 (e.g. rifampicin and omeprazole)
20. Ongoing pregnancy and women with childbearing potential not using any form of efficacious contraception during study and 3 months after the end of study.
21. Ongoing lactation and during the 3 months after the end of study.
22. Known hypersensitivity to Riluzole

## Informed consent

Written informed consent will be provided by each patient before any study-related procedures will be performed. After having received extensive information about the objectives, character and risks of the trial, and after sufficient time and opportunity for the patient to inquire about details of the trial and to take his (her) decision, the patient has to give his / her written consent by signing and dating the Informed Consent Form. Simultaneously this form will also be dated and signed by the Investigator. Obtaining of consent will be confirmed in the subject’s medical chart.

Three copies of the Informed Consent Form will be signed. The patient will receive one and the other will be filed in the Study File. The last one (sealed in anonymous envelop) will be kept in secure place by the sponsor at the end of the study.

A subject is considered as enrolled in the trial when the informed consent form has been signed.

If any new information that could influence the patient’s decision to stay in the trial becomes available, this will be transmitted without delay to the patient and the Investigator.

## Recruitment modalities

Patients will be screened and recruited among outpatients consulting for SCI in the different centers participating to the trial, over 24 months. In the 4 urban areas (2.55 million inhabitants), the number of patients with SCI is estimated to be 15700 (estimation from Schema Regional Orientation Santé 2007-2012). More precisely, active list of patients from the 7 centres show that the expected recruitment is realistic and achievable (overall recruitment rate of 0.6/month).

| Number of patients with chronic post traumatic SCI and Spasticity | Number of patients with BTX-A treatment per year | Number of patients with IT baclofene treatment per year | Number of new patients with chronic SCI/year |
| --- | --- | --- | --- |
| 931 | 210 | 93 | 161 |

In addition, patient’s associations will be contacted by OrphanDev network (FCRIN) and patients referred by the associations will benefit from a phone interview to check the main inclusion/non inclusion criteria before being referred to the closest investigation centre.

Patients fulfilling the eligibility criteria will be asked to participate in the study. Additional patients will be selected if some drop out from the study before first drug administration (whatever the reason : informed consent withdrawal, patients whose availability does not match with the study agenda...). Patients who drop out after first drug administration will not be replaced.

**Step 1**

| **Total necessary patients** | **30** |
| --- | --- |
| Number of active centres | 4 |
| Inclusion duration (months) | 8 |
| Number of patients / centre | 8 |
| Number of patients / centre / month | 1 |

**Step2:**

| **Total necessary patients** | **60** |
| --- | --- |
| Number of active centres | 7 |
| Inclusion duration (months) | 16 |
| Number of patients / centre | 8 to 10 |
| Number of patients / centre / month | 0.5 |

# STUDy TREATMENTS

## Experimental drugs: name, description, administration and dosage

Riluzole, a benzothiazole Na^+^ channel blocker, also known to inhibit presynaptic Ca^2+^-dependent glutamate release (Wang et al., 2004), is the only drug approved by FDA and EMA to prolong life expectancy or delay the use of mechanical ventilation in patient suffering from amyotrophic lateral sclerosis. Riluzole is rapidly absorbed after oral administration with a peak of plasma concentrations within 60 to 90 minutes (Cmax = 173 ± 72 (DS) ng/mL). Approximately 90% of the dose is absorbed. The absolute bioavailability of riluzole is 60% ± 18%. It is extensively metabolized by cytochrome P450 and subsequent it undergoes glucuronidation. In vitro studies on preparations of human liver cells have shown that cytochrome P450 1A2 is the principal isoenzyme involved in the metabolism of riluzole.

*Description of experimental drug:*

The management and the circuit of experimental drug will be realized within the pharmacy at the Sponsor site, under the accountability of the pharmacists associated to this project (Pr HONORE, Dr COHEN, UF3536, Expertise pharmaceutique et recherche biomédicale).

Preparation:

Preparation of experimental drugs will be realized within the pharmacy at the Sponsor site. Capsules of riluzole 25 or 50 mg will be conditioned in ivory/ivory capsules size 0. Placebos, only containing microcrystalline cellulose, will be conditioned in the same capsules (ivory/ivory capsules size 0).

Packaging and labeling:

Then, prepared capsules will be packaged by 28 in a labeled bottle.

Labeling of bottles will be realized in accordance with GCP and local regulations with the following elements: Clinical trial number, Sponsor name and address, principal investigator, dose, batch number and expiry date, as proposed below:

- Riluzole or placebo capsules 25 or 50 mg (step 1).

The experimental drug will be labeled as follows:

| **ASSISTANCE PUBLIQUE - HOPITAUX DE MARSEILLE**  **80 rue Brochier 13005 Marseille 05 (04 91 38 27 47)**  **Riluzole for the treatment of spasticity in the traumatic chronic spinal cord injury condition: Adaptive, Multicenter, placebo-controlled, randomised, double blind trial in a Rare Disorder”RILUSCI Study – Phase 1**  **(EudraCt : 2016-000901-35)**  Investigateur coordinateur : Pr Jean Michel VITON  **Riluzole ou placebo 25 mg**  **28 gélules**  **Initiales patient :_____/_____ N° patient : ………….**  **N° traitement : ………………**  **N° de lot : …………….. DLU : ………………………**  *Tenir hors de la portée et de la vue des enfants*  *Respecter les doses prescrites*  *Utilisation sous stricte surveillance médicale (art R5123 du CSP) - Conservation à température < 25°C*  **MEDICAMENT POUR ESSAI CLINIQUE UNIQUEMENT** | **ASSISTANCE PUBLIQUE - HOPITAUX DE MARSEILLE**  **80 rue Brochier 13005 Marseille 05 (04 91 38 27 47)**  **Riluzole for the treatment of spasticity in the traumatic chronic spinal cord injury condition: Adaptive, Multicenter, placebo-controlled, randomised, double blind trial in a Rare Disorder”RILUSCI Study – Phase 1**  **(EudraCt : 2016-000901-35)**  Investigateur coordinateur : Pr Jean Michel VITON  **Riluzole ou placebo 50 mg**  **28 gélules**  **Initiales patient :_____/_____ N° patient : ………….**  **N° traitement : ………………**  **N° de lot : …………….. DLU : ………………………**  *Tenir hors de la portée et de la vue des enfants*  *Respecter les doses prescrites*  *Utilisation sous stricte surveillance médicale (art R5123 du CSP) - Conservation à température < 25°C*  **MEDICAMENT POUR ESSAI CLINIQUE UNIQUEMENT** |
| --- | --- |

- Riluzole or placebo capsules 25 or 50 mg (step 2).

The experimental drug will be labeled as follows:

| **ASSISTANCE PUBLIQUE - HOPITAUX DE MARSEILLE**  **80 rue Brochier 13005 Marseille 05 (04 91 38 27 47)**  **Riluzole for the treatment of spasticity in the traumatic chronic spinal cord injury condition: Adaptive, Multicenter, placebo-controlled, randomised, double blind trial in a Rare Disorder”RILUSCI Study – Phase 2**  **(EudraCt : 2016-000901-35)**  Investigateur coordinateur : Pr Jean Michel VITON  **Riluzole ou placebo 25 mg**  **28 gélules**  **Initiales patient :_____/_____ N° patient : ………….**  **N° traitement : ………………**  **N° de lot : …………….. DLU : ………………………**  *Tenir hors de la portée et de la vue des enfants*  *Respecter les doses prescrites*  *Utilisation sous stricte surveillance médicale (art R5123 du CSP) - Conservation à température < 25°C*  **MEDICAMENT POUR ESSAI CLINIQUE UNIQUEMENT** | **ASSISTANCE PUBLIQUE - HOPITAUX DE MARSEILLE**  **80 rue Brochier 13005 Marseille 05 (04 91 38 27 47)**  **Riluzole for the treatment of spasticity in the traumatic chronic spinal cord injury condition: Adaptive, Multicenter, placebo-controlled, randomised, double blind trial in a Rare Disorder”RILUSCI Study – Phase 2**  **(EudraCt : 2016-000901-35)**  Investigateur coordinateur : Pr Jean Michel VITON  **Riluzole ou placebo 50 mg**  **28 gélules**  **Initiales patient :_____/_____ N° patient : ………….**  **N° traitement : ………………**  **N° de lot : …………….. DLU : ………………………**  *Tenir hors de la portée et de la vue des enfants*  *Respecter les doses prescrites*  *Utilisation sous stricte surveillance médicale (art R5123 du CSP) - Conservation à température < 25°C*  **MEDICAMENT POUR ESSAI CLINIQUE UNIQUEMENT** |
| --- | --- |

Whatever the step of the clinical trial, Riluzole or Placebo capsules will be administered, twice daily (morning and evening), before meals from Day 1 to Day 14.

For the step 2, the daily dose administration will be determined in the first step of this study using a Bayesian method.

The Sponsor will provide blinded experimental drug for all subjects to the investigating centers, by the Timone hospital pharmacy which has the proper agreement. Pharmacy documents for traceability (Reception form, Prescription form, patient log, product log, destruction log and pharmacy delegation task) will be designed by the coordinating pharmacy.

Storage and Handling:

Labeled bottles of experimental drug prepared capsules will be stored within the pharmacy at the Sponsor site at a controlled temperature < 25 °C before shipment to the pharmacy of other recruiting centers.

No special care is required to handle the experimental drugs.

Methods of assigning subjects to treatments, accountability, and destruction:

The experimental drug will be supplied to each subject in a kit containing sufficient labeled bottles of experimental drug for fourteen days at the visit 2 (V2) together with the drug diary. This delivery will be realized by the pharmacy which has first received a nominative prescription from the authorized investigator of the recruiting center. All documents for drug traceability will be completed by the investigating pharmacist. The pharmacist reports the name, dosage of drug and the specific number of allocated drugs given to patient at dispensation.

Patients will be asked to return all used and unused bottles together with the drug diary at the visit V4. Medication compliance will be checked at the visit V4 by the investigating pharmacy.

Destruction of experimental drugs will be performed by the investigating pharmacist upon request of the sponsor.

Blinding :

The experimental drugs will be namely supplied to each subject by the pharmacy of each recruiting center. In order to maintain the double blind process as to investigators and subjects, the randomization will be managed by the pharmacist accountable of clinical trials in each recruiting center. Advices concerning the experimental drugs administration will be dispensed by this pharmacist.

## Concomitant treatments

All drugs currently used in chronic SCI patients will be recorded with: name, dose, regimen, dates of start and stop.

Last injection of BTX-A in striated muscles should be more than 3 months ago and patients must have returned to their usual level of spasticity before BTX-A injection. Last injection IT of baclofen or per os administration of any myorelaxant should be more than 14 days ago (Step 1) or should be stable for ≥ 30 days prior to screening also be kept at stable daily dose until the end of the protocol (Step 2).

Due to potential drug/drug interaction, drugs that interfere with CYP1A2 will be withdrawn at least 5 half life before inclusion.

Other drug treatments, including analgesics should be stable for ≥ 30 days prior to screening. BTX-A injection in detrusor in allowed.

All change of drug treatment decided by the investigator should be written in the CRF, with the reason of such change (AE or other reason).

## Drugs interaction

There have been no clinical studies evaluating the interactions of riluzole with other drugs. *In vitro* studies using preparations of human liver microsomes suggest thatCYP1A2is the principal isoenzyme involved in the initial oxidative metabolism of riluzole. Inhibitors of CYP1A2 (as diclofenac, diazepam, nicergoline, clomipramine, imipramine, fluvoxamine, theophylline, amitriptyline and quinolones) could potentially decrease the rate of riluzole elimination, while inducers of CYP1A2(including cigarette smoke, food smoke charcoal, rifampicin and omeprazole) could increase the rate of riluzole elimination

(http://www.ema.europa.eu/docs/en_GB/document_library/EPAR_Product_Information/human/000109/WC500056586.pdf).

# DRUG CONCENTRATIONS

## Drug Concentration Determination

Riluzole concentration in plasma is quantified using a high performance liquid chromatography (HPLC) assay with U.V. detection, adapted from Chow et al, (2012), after a liquid-liquid extraction step from a 200µl plasma sample. The assay is linear from 5 to 500 ng/ml, with a lower limit of quantification of 5 ng/ml.

For wash-out checking purpose, baclofen concentrations in plasma will be quantified using a liquid chromatography-tandem mass spectrometry assay (LC-MS/MS) after a solid-liquid extraction step. The assay is linear from 2 to 200 ng/ml, with a lower limit of quantification validated at 1 ng/ ml. Expected plasmatic concentrations of baclofen after IT administration are low (Albright and Shultz, 1999; Sallerin and Lazorthes, 2003).

## Plasma samples collection

Blood samples (one 5 ml tube red top without additive) for riluzole PK study will be repeatedly collected at predose (immediately prior to riluzole intake) and 2 h postdose for trough and peak concentrations, respectively, on Days 1, 3 and 14 after the initial dose. (6 samples/patient).One blood sample (one 5 ml tube red top without additive) for baclofen determination will be collected upon inclusion visit (Step 1). For the patients (Step 2) who are under baclofen treatment, two blood samples for baclofen observance study will be repeatedly collected immediately prior to riluzole intake and 2 h post-dose after riluzole intake respectively, on Days 1, 4 and 14 after the initial dose. (6 samples/patient).

If possible samples will sent in the same day to the laboratory in charge of pharmacokinetic (whole blood in ambient conditions) If not possible, all samples after pre-analytic process, will be frozen and stored at -20°C.

All patients who have been enrolled in the study will be asked to donate optional blood samples. These specimens will be used for research purposes to identify and/or verify:

Inherited DNA polymorphisms known or hypothesized to be associated with riluzole activity (two 5 ml tube purple top with EDTA) will be taken for DNA extraction from every patient after genetic informed consent at predose at V1. If possible samples will sent in the same day to the CRBM (whole blood in ambient conditions) If not possible, all samples will be stored at +4°C.

Protein biomarker discovery and validation (one 5 ml tube red top without additive) at V1 and V4. If possible samples will sent in the same day to the CRBM (whole blood in ambient conditions) If not possible, all samples after pre-analytic process, will be frozen and stored at -20°C.

A lab manual will be provided.

Samples (1 for the pharmacogenetic and 2 for biomarker per patient) will be stored for 5 years after final database freeze and then destroyed. The storage will be done to the CRB TAC - Centre de Ressources Biologiques Tissus, ADN, Cellules – Département de Génétique Médicale de l’hôpital de la Timone. This laboratory is declared under the number DC 2008-428 at the ministry of « Enseignement Supérieur et de la Recherche » and it is authorized to storage the elements of the human body under the number AC on 2011-1312

The blood samples for the analysis of biomarkers will be preserved with the CIC (AP HM) declared under the number DC 2011-1369

# STATISTICAL METHODS

A Statistical Analysis Plan (SAP) containing detailed methods will be provided before the study begins. Bayesian analysis will be performed by F-CRIN Platform (Dr. Corinne Alberti). Patients’ description, secondary analysis and safety analysis will be performed by the CIC-CPCET (Elisabeth Jouve). Analyses will be conducted in respect of international guidelines and recommendations for clinical trials (ICH, CONSORT) and following internal Standard Operating Procedures.

Quantitative variables will be described as medians [quartiles] or means (standard deviation) depending on the Gaussian distribution or not. Qualitative variables will be described as numbers (percentages). Inter-group comparisons will be conducted using parametric or non-parametric tests according to the nature and the distributions of the variables. Unless otherwise specified, statistical significance is defined as p<0.05.

## Sample size justification

For the first step: the characteristics of the CRM method with Bayesian inference have shown that the inclusion of 30 patients is sufficient for this type of study [O'Quigley et al, 1990; Zohar, 2003]. Stopping rules will be sequentially applied to eventually stop the inclusions before this number.

For the second step, the expected number of patients is arbitrary and conditioned by 1) the recruitment opportunities of all centers involved in the study, 2) the lack of knowledge on success rate of Riluzole inducing a too large sample size for a frequentist approach. The number of patients over 16months will be 30 patients per group randomization (e.g. 60 patients total).

## Populations analysed

In step one, all patients with a valid outcome will be analysed for the CRM.

In step two, the “intention to treat” population will be analysed.

## Patient description

All data will be listed and summary tables will be provided for all included patients, as well as by treatment dose level (step 1) and by treatment group (step 2)

Quantitative data will be described by the following summary statistics: arithmetic mean, standard deviation, 95% confidence interval of the mean, median, inter-quartile range (25th and 75th percentiles), minimum and maximum. Summary statistics will be provided for the original data as well as for the change at each visit versus baseline, where appropriate. Baseline values will be the last values before randomization. Frequency tables will be provided for qualitative data. Graphical illustrations will be provided where appropriate.

Disposition of patients will be describe for each step by treatment groups. The number of patients who prematurely dropped out will be summarized by reason as reported in the CRF.

Demographic and baseline characteristics data will be summarized for all analyzed populations, by step and treatment groups. Medical history findings will also be summarized. They will be classified by system organ class and preferred term (MedDRA dictionary).

Comparability of treatment groups at baseline will be verified using statistical methods for comparison of independent samples according to the nature and the distribution of the variables.

## Pharmacokinetics Analysis

Pharmacokinetics parameters for riluzole will be calculated using standard Bayesian population approach using a reference population matrix already made available in the literature (Bruno et al., 1997). Stationarity of the PK parameters over the treatement (i.e., D0-D14) will be evaluated. Exposure levels (i.e., truncated AUCs) will be calculated. Pharmacokinetics will be studied using compartimental modelling with dedicated tools (KineticPro, MonoLix).

## Statistical Analysis of primary endpoint (F-CRIN Platform, Dr Corinne Alberti)

To assess the dose-response relationship of Riluzole for spinal cord injury, the continual reassessment method (CRM) (O'Quigley et al., 1990) based on Bayesian inference with a modification in order to control outlier observations (Resche-Rigon et al., 2008) will be used. The aim will be to determine the minimal effective dose (MED) of Riluzole in 75% of patients. The study will be double blind and the main judgment criteria will be binary (success or failure).

The CRM is sequential Bayesian method based on a one-parameter model, which aims at estimating the percentile of dose-response among k distinct dose levels d_i (i=1,...,4)_. Each one of the four dose levels is associated by the investigators (according to his/her personal experience and available data in the literature at the time of initiation of the trial) with a prior estimated success probability.

Then, a one-parameter power model will be used to fit the dose-response curve, with an exponential prior distribution for the model parameter. The posterior response probability of each dose level will be re-estimated after each new inclusion of cohort patients (2 patients per cohort). The allocated dose to each new cohort of patients will be the dose level with the updated posterior response probability closest to 0.75. The first cohort of patients will receive a loading dose randomly chosen by F-CRIN (Dr Alberti) with blinding of both patients and investigators. The minimum ED (MED) will be defined as the dose level among the four chosen doses that had a final response probability closest to the target.

The decision to end the study will be based on stopping criteria, in order to detect whether all doses were likely to be inefficient or a suitable estimation of the MED has been reached. The study will be conducted in two steps: 1) Determination of the MED among the four doses of the panel 2) Estimation of the probability of response associated to the DME.

After the inclusion of 30 patients, the minimum efficient dose will be the dose whose mean updated success rate closest to the target success rate of 75%. However, the first stage analysis can also be terminated earlier if there is high evidence that a specific dose will remain unchanged for the next two hypothetical groups of two further patients: *P(unchanged dose|data, z=4)>0.85, stop of the first stage and passage in second stage* or if no dose is estimated to be efficient enough *P(d_4_<target)>0.85*. If the first criterion is satisfied then the dose allocation process will be stopped and the second stage analysis will begin and if the second criterion is satisfied then the trial will be stopped.

The second stage of analysis will ensure that the trial will not terminate too early if a minimum efficient dose is previously selected. Thus, the inclusions will be pursued at this dose level in order to obtain reliable estimates of the success rate. Again, the second stage analysis will be terminated after the inclusion of the total number of patients (n=30 per group) or if there is a high evidence that outcomes from another two hypothetical groups of two patients would not provide further gain on precision of the estimation of the success rate *P(max of the width of the credibility interval |data, z=4)<0.05*. Early termination of the study will be validated by the investigator, the sponsor and the independent monitoring board of the study.

To assess the efficacy of Riluzole compared with placebo on the spasticity of chronic SCI patients, a Bayesian approach will be used. The latter is to update the *a priori* information on the expected success rate from the observations. This produces an expected response rate revalued *a posteriori*.

Statistical analysis will be performed sequentially after each observation of the response at two weeks of patients enrolled. It is to sequentially estimate the probability of observing a success using a Bayesian approach with a beta-binomial model (Berry D A. Statistics: a Bayesian perspective Ed.:. Duxbury, Belmont Californie 1996)

The Bayesian approach is to consider the success rate as a random variable with prior density centered on the expected rate of success. The construction of the prior density is a combination of data from the literature and examination of a panel of experts, before the beginning of the trial.

Two beta densities will be chosen, defined by two parameters a and b considering two types of opinion (i) optimistic and (ii) pessimistic from the elicitation of experts and literature. The *a priori* expected mean of each density will therefore centered on $\left(\pi=\frac{a}{a+b}\right)$based on those opinions. Several choices will be made for the variance given by $\left(\frac{ab}{\left(a+b\right)^{2}\left(a+b+1\right)}\right)$ allowing giving more or less weight on observations relative to the initial *a priori* on the rate of success (more the variance is large, more the weight of observations increases).

The Bayesian estimator of the success rate is the expected value of the *a posteriori* distribution, whose parameters are defined after *n* inclusions by $a_{n}=a+r$ and $b_{n}=b+n-r$ in each arm, where *r* is the number of successes observed on *n* inclusions. So we obtain the following expected value in each arm: $\tilde{\pi}=\frac{a+r}{a+b+n}$.

The difference between the response rates in both arms of randomization is modeled by the random variable $\Delta$. We will study the random variable $\Delta$, i.e. its distribution and credibility intervals as depending on optimistic and pessimistic views of the expertise.

We will also calculate the predictive distribution of the number of successes over the next *k* inclusions or the remaining patients scheduled in each arm. Because of the relative small number of patients, there are no stopping rules based on Bayesian sequential analysis.

Descriptive statistical analyses will be performed with the SAS 9.3 (SAS Inc, Cary, NC, USA) software package for PC and Bayesian statistical analysis with a program written in C language (Zohar, 2003).

## Statistical Analysis of secondary endpoints

The following secondary endpoints will be analysed :

- Electrophysiology parameters : negative peak duration, baseline-to-peak amplitude, mean F-wave amplitude, soleus H reflex and surface EMG (assessed in one centre, analysis of this last parameter will be only descriptive)
- Patient Global Impression of Change : score 1 (no change) to 7 (a great deal better and a considerable improvement that has made all the difference)
- Penn Spasms Frequency Scale : 1+1 component, frequency of spasms scored from 0 (no spasms) to 4 (Spontaneous spasms occurring more than ten times per hour) and severity of spasms scored from 1 (mild) to 3 (severe)
- Numeric Rating Scale (NRS) for the measurement of spasticity : from 0 to 10
- Ashworth score : (6 levels)
- 0 No increase in tone
- 1 Slight increase in muscle tone, manifested by a catch and release or minimal resistance at the end of the ROM when the affected part(s) is moved in flexion or extension
- 2 Slight increase in muscle tone, manifested by a catch, followed by minimal resistance throughout the remainder (less than half) of the ROM
- 3 More marked increase in muscle tone through most of the ROM, but affected part(s) easily moved
- 4 Considerable increase in muscle tone, passive movement difficult
- 5 Affected part(s) rigid in flexion or extension
- Visual Analog Scale Pain : score from 0 to 10
- Neuropathic Pain Symptom Inventory: sum of the scores of the 10 descriptors
- Spinal Cord Injury Independence Measure (SCIM scale): The SCIM includes the following areas of function: self-care (subscore (0-20), respiration and sphincter management (0-40) and mobility (0-40). Each area is scored according to its proportional weight in these patients' general activity. The final score ranges from 0 to 100.

Values and scores will be described by visit and changes will be calculated at Week 2 from baseline. Changes will be compared between the two treatment groups using a Student t-test or a Mann-Whitney test. Few scores cannot be analyzed as continuous variables (severity of spasms in particular) and will be compared as frequencies using a McNemar test.

## Safety analysis

The safety analysis will be performed on the safety population which will include all the exposed patients, i.e. all patients who will have taken at least one dose of study treatment in step 1 or at least one dose of randomized treatment in step 2. The safety analysis will be based on the reported adverse events. Each AE will be coded to a "preferred term" and associated "system-organ class" according to an established and validated adverse reaction dictionary (MedDRA). The AE endpoints are number of patients experiencing:

- at least one event,
- an event under each recorded preferred term,
- an event under each recorded system-organ class.

These endpoints apply to all AEs, regardless of relationship of the event to the study treatment.

AE will be classified as:

- Treatment emergent adverse events (TEAEs): AEs that occurred or worsened during an exposure to drug(s);
- Non-treatment emergent adverse events (NTEAE): AEs that occurred before the first study drug administration.

Individual data listings of all adverse events will be provided. NTEAEs will be only presented as individual data listing.

Analysis of TEAEs will be performed primarily by descriptive statistics. AE endpoints will be summarized by riluzole treatment dose level at step 1 and by treatment group, as treated basis, at step 2. Similar tabulations will be performed to summarize the characteristics of TEAEs: relationship to treatment, severity, delay, duration, as it will be relevant.

# SAFETY – RISkS

## Description of laboratory safety parameters

Patients will undergo laboratory screening analysis including haematology, biochemistry, serologies (only at screening) and urinalysis during all study period as follow:

- **Haematology**: red blood cell (RBC), hematocrit (Ht), hemoglobin (Hb), white blood cell count (WBC) with differential (neutrophils, eaosinophils, basophils, monocytes and lymphocytes), platelets, INR and aPTT;

- **Biochemistry**: sodium, potassium, chloride, calcium, phosphorus, lactic dehydrogenase, creatine phosphokinase, AST, ALT, alkaline phosphatases, amylase, lipase, gamma-glutamyl transferase (GGT), total and conjugated bilirubin, creatinine, urea, uric acid, glucose, albumin, total proteins, total cholesterol, triglycerides,

- **Serologies**: hepatitis B antigen (HBs Ag), Hepatitis C antibodies (Anti-HCV Ab), Anti-HIV1 and Anti HIV2 antibodies (done at screening only)

- **Urinalysis**: proteins, glucose, blood, ketone bodies, pH.

All analyses will be performed by a local laboratory.

## Management of adverse events

*Definitions (R1123-39 of Public Health Code)*

**Adverse event (AE)**

Any noxious or undesirable event experienced by a participant during a clinical trial, whether or not considered related to the experimental drug should be considered as an adverse event.

**Serious adverse event (SAE)**

A serious adverse event is an adverse event that is:

- Fatal
- life-threatening
- significantly, persistently or permanently disabling
- requiring in-patient hospitalization or prolongation of hospitalization
- requiring intervention to prevent permanent impairment or damage
- Any event that could be considered as potentially harmful
- Any event medically accurate according to the investigator’s judgment

In addition, congenital abnormalities, occurrence of malignancy or clinical injuries resulting from overdose are always considered as SAE.

**Adverse drug reaction (ADR)**

An adverse drug event (ADE) refers to any noxious or undesired reaction occurring at the time a drug is used, whether or not it is identified as a cause of the reaction. An ADR is a special type of ADE in which a causative relationship can be shown.

**Unexpected adverse drug reaction (UADR)**

An unexpected adverse event is an event not previously reported (in nature, severity or incidence), i.e. not included in the current Investigator's Brochure or in the International Product Information Document, or local package insert.

## Investigator’s liability

### Regulatory obligations of investigator (Art. R. 1123-54)

**Modalities of collection, verification and presentation of adverse events**

All adverse events occurring during clinical trials have to be collected, verified, registered and reported from the signature of the informed consent until the last day of the study, or as soon as the investigator becomes aware of the AE that he considers linked or not linked to the protocol and this up to its resolution.

Adverse events are collected:

- During clinical examination, from blood sample analysis, or from the investigator questioning the patient

- from the patient’s unsolicited reporting, as encouraged to do towards the investigator

### Protocol particularities

#### Event requiring a specific action

#### In case of aggravation of spasticity under treatment, the investigator should search for an intercurrent condition that is likely to explain this aggravation in SCI patients. Such conditions include: infection, lithiasis, fecaloma, bedsore… (non limitating list). For patients receiving IT baclofen infusion (Step 2), the investigator will first check the pump. These conditions/events are first to be treated. In case of any condition has been identified OR the spasticity does not improve after intercurrent condition/event treatment, experimental drug is to be stopped (failure).

### Notification to the sponsor by the investigator

**Serious adverse events reporting (SAE)**

The investigators must immediately report any serious adverse events as defined above to AP-HM, the sponsor.

The investigator must search for evidence of a causality between the drug and the SAE, and supply when possible a medical prognosis. The investigator must provide information on symptoms, time of onset, subsidence, action taken and subject outcome.

The SAE Form must be sent to the sponsor together with the hospital reports, examination reports and biological results related to the SAE (including negative results); Single patient reports must be anonymised and bear a code/randomization number.

Once completed, dated and signed, the Serious Adverse Event Form must be addressed by fax to:

**Direction of Clinical Research and Innovation of AP-HM**

**80, rue Brochier, 13354 Marseille Cedex 05**

**Phone 04 91 38 27 47 Fax: 04 91 38 14 79**

**E-mail: drci@ap-hm.fr**

All subjects with SAE must be followed up for outcome until resolution (even if the patient dropped out/was excluded from the study); reports must be sent to the sponsor within 8 days of the initial SAE declaration by fax or email.

**Causality link**

The investigator must assess the causality link between the SAE and the experimental drug. If a causality link is suspected, the investigator/sponsor will regard the SAE as a Suspected Unexpected Serious Adverse Reaction (SUSAR).

### Notification period to the sponsor

For each person taking part in research, the investigator is held to notify:

- starting from the date of signature of the consent
- during the treatment period
- up 4 weeks after the end of treatment period
- without limitation of time, when the SAE is likely to be due to the experimental drug or the procedures of research (for example of the serious event being able to appear at long distance from the exposure to the drug, such of cancers or the congenital anomalies).

Any undesirable event and SAE is to be followed until its complete resolution (stabilization on a level considered to be acceptable by the investigator or backward recovery) even if the patient left study.

### Sponsor’s role

#### Unexpected SAE declaration:

The sponsor must assess the causality link between the SAE and the experimental drug.

The sponsor will also report all expected/unexpected SAE based on the SPC (Summary of Product Characteristics).

The sponsor will assume the responsibility for appropriate reporting of adverse events to the European Medical Agency, Ethics Committees and relevant Health Authorities.

The regulatory delays for SAE declaration are:

- 7 days for SAE, fatal SUSARs or life-threatening SUSARs. A further delay of 8 days is granted for full and accurate documentation of the case

- 15 days for all other SUSARs. A further delay of 8 days is granted for full and accurate documentation of the case

- In double-blinded studies, the sponsor must report the SUSAR to the relevant Health Authorities and Ethics committees as soon as possible after the unblinding visit.

### Annual safety report

It will be managed by the DRCI's pharmacovigilance platform, and send to the competent health authorities.
 At the date of the anniversary for the trial authorization issued by Health Authorities and Ethics Committees, the sponsor must write a safety report including:

- The list of serious adverse event that may be linked to the experimental drug including unexpected and expected serious events.

- A critical assessment of patient safety suitable for research.

This report can be submitted for approval to the principal investigator.

The sponsor must send the Annual Security Report within 60 days of the trial authorization

## Data safety monitoring board (DSMB)

The Data safety monitoring board (DSMB) will be set up by the sponsor. It has main mission to follow-up the security data. It can have additional missions, to follow-up the effectiveness data. DSMB is mentioned with the article L. 1123-7 of Public Health Code.

DSMB puts forth recommendations with the sponsor on the continuation, the modification or the stop of research. The recommendations which can be emitted by a DSMB are:

- continuation of research without modification,
- continuation of research with modification of the protocol and/or the monitoring of the patients,
- temporary stop of inclusions,
- final adoption of research to the glance as of:
  - data of security : serious undesirable effects,
  - data of effectiveness : futility or shown effectiveness.

The DSMB is constituted by 3 independent persons, 1 pharmacologist, 1 Physical and Reeducation Medicine specialist, and a methodologist. None of them will have interest link with either the tested drug or research.

DSMB has an advisory function on points of security such as tolerance and the reevaluation of the benefit-risk ratio during research. This committee will have also a role to make sure of the conformity of the protocol.

DSMB will meet at the end of Step 1. In the event of anomaly, this committee will meet in real time.

A charter will detailed methods of this committee and will be approved by sponsor and DSMB.

The sponsor remains decision-maker. He transmits if necessary his decision argued as well as the reports of DSMB to the competent authority (ANSM) and CPP.

# DATA COLLECTION

## Data access

In according with GCP:

- the sponsor is charged to obtain the agreement from all involved parties to guarantee the direct access with all data source, documents source and all study records with an aim of audit and quality control by the sponsor,

- the investigators will agree to allow the sponsor’s representatives and any regulatory agencies a direct access to all documents and individual data strictly necessary to this control.

## Source documents and record retention

Source documents are defined as any original documents or data and records (hospital records, patient’s diary, evaluation check-list, pharmacy dispensing......)

All documents and data will be retained during 15 years by the investigator after the completion of the study.

## Data confidentiality

The sponsor’s monitor (L.1121-3 article of Public Health Code), will take all the precautions to maintain the confidentiality of patients' identities and sponsor’s proprietary information.

All parties are subjected to the professional secrecy(according totheconditionsdefinedbyarticles226-13and226-14ofthepenal code).

All data collected on patients, documents that should identify patients and transmitted to the sponsor by the investigators will be made anonymous.

Identification of patients :

Only the initials (name and first name) will be recorded, accompanied by a number coded suitable for research indicating the order of inclusion of the patients in each center.

The sponsor will make sure that each patient participating to the research gave her/his written consent to the access to the individual data relating to it and strictly necessary to the quality control of the research.

## Data ownership

AP-HM is the owner of the data. Data will be disclosed to AMU-CNRS Institut des Neurosciences Timone, the Orphan Drug Designation holder, for further exploitation. Each party is to receive fair compensation for the commercial use of the results that it has helped to generate. The data will not be used or disclosed to any other third party without AP-HM and AMU-CNRS Institut des Neurosciences Timone prior approval.

# Data management

The clinical data management will be carried out by the CIC-CPCET in respect of international guidelines and recommendations for clinical trials (ICH, CONSORT) and following internal Standard Operating Procedures. A Data Management Plan (DMP) will be established for the study. Patient data will be recorded using an electronic Case Report Form (eCRF) from the paper source documents by investigator sites. Source document verification will be conducted by the study monitor (see 13 Monitoring and quality control) to ensure accuracy and reliability of data collection. Validation checks will be implemented using front-end checks in the e-CRF and back-end checks in the database to verify the data. The clinical data management system will include an audit trial. Discrepancies will be resolved with the investigator sites (queries). Adverse events and concomitant medications will be coded using validated dictionaries (MedDRA, ATC). A SAE data reconciliation will be carried out. A data blind review meeting will conclude the process of data management. In particular, the coding, the potential protocol deviations, and the status of patients will be validated. The database will be locked once all requested investigators and decisions made during the review of data have been incorporated into the data files. Any subsequent changes should be a request for unlocked. The study database will be archived with all the documentation of the data management process.

# Quality contrOl

Every biomedical research project supported by the AP-HM is classified according to the estimated risk for persons participating in research through the classification of biomedical research to promote AP-HM A to D.

## General organization

The sponsor must ensure the safety and respect of the people who agreed to participate in research. It must establish a quality assurance system to ensure that the trial is performed and the data are recorded, and reported in compliance with Good Clinical Practice (GCP)

To this end, the Clinical Research Associates (CRA) primary mission is to conduct regular monitoring visits to the research sites.

A site visit will be performed at the start of the study to review the study protocol in details and assure the availability of appropriate personnel and GCP procedures. The objectives of the monitoring visits, as defined in the Good Clinical Practices (GCP § 5.18.1) are to ensure that:

• the respect of the law, security and protection of patients are guaranteed,

• reported data are accurate, complete and consistent with clinical files

• the research is conducted in accordance with protocol, GCP and the applicable laws.

### Opening strategy

The opening strategy implementation for this research is determined by the adapted monitoring plan.

### Monitoring of the centers

In the case of the D-risk research (according to French Minister of Health procedures for public clinical research), the choice of an appropriate level of monitoring was weighted according to the complexity, impact and research budget. To this end, the developer in accordance with the coordinating investigator determined the logistics and impact score that yielded the level of monitoring to establish research: high.

Monitoring will consist of verification: the existence of included patients, the signed informed consents, criteria of eligibility, the primary endpoint, secondary endpoints, AEs / SAEs / tolerance, pharmacy management and randomization, on scheduled monitoring.

## Quality control

A Clinical Research Assistant (CRA) nominated by the sponsor will ensure the successful completion of the research, data collection, their documentation, recording and reporting in accordance with the DRCD’s Standard Operating Procedures and in accordance with Good Clinical Practice and the laws and regulations in force (applicable French regulatory requirements).

The investigator and his team agree to be available during visits of quality control. During these visits, the following items will be reviewed:

- Written consent

- Compliance with the protocol and procedures

- Data quality in the CRF : missing data, data consistency with documents "source" (medical records, appointment books, original laboratory results, etc..)

- Management of the used treatments.

## CRF

All information required by the protocol must be recorded on the case report forms by the Investigator, or designated representative and an explanation must be provided for each missing data. Data should be collected as and when they are obtained and transcribed in these CRF.

Erroneous data collected on case report forms will be crossed out and the new data will be written to the information side closed, accompanied by initials, date and possibly justified by the investigator or the person authorized to be making the correction. If an e-CRF is used, different process may apply but principles of tracability remain the same.

## Study Deviations

Any event occurring due to non compliance with the protocol, standardized operating procedures, good clinical practices or laws and regulations by an investigator or any other person involved in the conduct of the research should be a statement non-compliance with the sponsor. At first, these statements will be reviewed and processed by the medical coordinator in DRCI to take corrective or preventative actions, then in a second time sent to the plateform BPC-PV of DRCI for verification and analysis. These audits may be a request for information, visits or audit compliance with the investigator in charge of the place of research concerned.

## Audit / inspections

Investigators agree to accept the quality assurance audits conducted by the sponsor as well as inspections by the competent authorities. All data, all documents and reports may be subject to audits and regulatory inspections can be opposed without medical confidentiality.

An audit may be conducted at any time by persons authorized by sponsor and independent of the research to ensure the quality of research, the validity of its results and compliance with the law and regulations in force it aims.

People who supervise research agree to comply with the requirements of the sponsor and to the competent authority regarding an audit or inspection of the research.

The audit can be applied at all stages of research, protocol development to publication of results and archive of data used or generated by the research.

# ETHIc and LEGAl aspects

## Information of patient and consent collect

In accordance with article L1122-1-1 of Code de Santé Publique, no biomedical research can be practised on a person without her free and informed consent. Prior to participation in the trial, the written informed consent form should be signed and personally dated by the patient. It collected after supply information and respect of reflexion delay, in accordance with Good Clinical Practices. Definitive inclusion will occur only after having obtained this consent.

### Information of the study’s patients

The investigator must orally explain the protocol and give each patient the information form during the screening visit. The information and consent forms will be signed by the investigator and the patient.

The patients will receive information about: the aim of the study, the duration of their participation, the procedures that will be followed and the constraints and risks related to the therapeutic protocol. The investigator will also explain the experimental nature of the study and uncertainties related to expected benefits, data confidentiality and insurance coverage. All of this information will be summarised on an information form.

### Patient’s consent

The patient must be given ample time between the information and signature of informed consent form (reflexion delay). Information will be summarised on an information form given to each patient.

Informed consent will be signed in triplicate by the patient and the investigating physician. One copy of this document will be given to the study participant; the investigator must keep the second copy in his/her archives for at least 15 years; the third copy will be given to the sponsor in a sealed envelope at the end of the study.

For biobanking and DNA extraction, an additional and specific informed consent is to be signed. Patients might refuse to sign this additional and specific informed consent form. In this case, they might be included but additional blood samples will not be taken.

### Information in the medical records

The patient’s enrolment in a trial must be recorded in the patient’s medical record. These data should identify the trial and should document the dates of the patient’s informed consent signature and dates of study participation.

### Modification of the informed consent form

The informed consent form will change if there are substantial modifications, under the cases and the conditions envisaged by the law (L .1123-9 and L. 1123-10 of Public Health Code).

## Forbidden of participation IN other research

Patients cannot take part in other biomedical research relating to drugs during the study. However patients can take part in other search for observational type.

At the end of the study, there is no exclusion period.

## Recruitment

During a medical consultation, investigator will explain the study to patients and will supply information letter. Patient’s association will also be informed. Patient from associations who are interested in participating to the study will be provided with a call center number to be refered to the closest study site.

## Study taken charge

The care of the patients included in this research was based on the care consultation except examinations and procedures added by research (cf.8)

## Compensatory damages

No compensatory damages will be offered to the patients. Reasonable compensation for travel expenses will be proposed to the included patients

## Legal obligations

### Sponsor’s role

L’Assistance publique hôpitaux de Marseille (AP-HM) is the sponsor of this study and transferred to “Département de la recherche Clinique et Innovation » (DRCI) the missions, in accordance with L.1121-1 of Code de Santé Publique. Assistance Publique - Hôpitaux de Marseille can discontinue the study for administrative or medical reasons.

### Investigator’s role

Before study starts, each investigator will provide the sponsor with a dated, signed CV with medical license number (RPPS number or Ordre des médecins). Each investigator will sign the protocol (certifying he/she will follow GCPs). Signature pages will be kept by the study sponsor.

### Request of Independent Ethics Committee opinion (CPP according to French Law)

Sponsor obtained for biomedical research the favourable opinion of CPP.

### Request of ANSM authorization

Sponsor obtained for biomedical research the authorization of ANSM

### Commitment of conformity to the CNIL « Méthodology of reference » MR 001

The sponsor signed a commitment of conformity to this “Methodology of Reference”.

## Study’s modifications

Any substantial modification made to the protocol by the coordinating investigator, will have to be transmitted to the sponsor for approval. After this agreement, the sponsor will have to obtain prior to his implementation a favourable opinion of CPP and an authorization of the ANSM. A new consent of the patients will be collected if necessary.

## Clinical study report

A report written according to the competent authority’s reference plan will need to be sent to the competent authority and ethical review board within one year after the end of research, which should be understood as the last follow-up visit of the last included patient.

# Financing and insurance

## Research’s budget

The research’s costs are :

- Protocol writing
- EMA protocol assistance process (OrphanDev)
- Treatment preparation and delivery (distribution on each site)
- Patient’s transportation fees
- Rare patient’s recruitement (OrphanDev)
- Staff : investigator, study nurse, CRA …
- Plasma concentration analysis
- Quality control by a sponsor’s CRA
- Vigilance costs
- Data management : eCRF/CRF, data management, statistic analysis
- Other costs : meetings
- Study Report writing
- Publication

## Insurance

In accordance with the legislation on biomedical research, the sponsor has taken out an insurance from the company SHAM for the full study period, covering its own civil liability and that of any agent (doctor or research staff) (Act no. 2004-806, Art L.1121-10 of the Public Health Code).

# publication’s rules

The coordinating investigator, the sites principal investigator, the program coordinator and the scientific director will be responsible for the publication strategy for this study and will author all papers. Several papers are expected focusing on clinical aspects, pharmacological aspects, methodological aspects, scientific aspects. For investigator’s signature, the number of included patients will be taken into consideration.

The AP-HM will be mentioned in the affiliations of the publication’s authors (when relevant). If an author has several affiliations, different institutions will be quoted (AP-HM, University, CNRS...)

AP-HM (DRCI) will be mentioned as “sponsor” of the study.

The funding source (PHRC national, Ministry of Health) will be mentioned in the “acknowledgments” section of the manuscript as follows: “The study was funded by a grant from Programme Hospitalier de Recherche Clinique - PHRC 2015 (Ministry of Health)”

The mention that the search is registered on the site Clinicaltrials.gov will also be quoted (with registration number).

# ANNEXES

## List of participants

**INVESTIGATORS**(Civility Name First name City Country Health facility Email Phone Speciality)

Mr. Viton Jean-Michel, Marseille, France, APHM, jean-michel.viton@ap-hm.fr +33491384616 PRM

Mr. Delarque Alain, Marseille, France, APHM, alain.delarque@ap-hm.fr +33491384616 PRM

Mr. Bensoussan Laurent, Marseille, France, APHM, laurent.bensoussan@ap-hm.fr +33491384616 PRM

Mrs. Kerzoncuf Marjorie, Marseille, France, APHM, [marjorie.kerzoncuf@ap-hm.fr](mailto:marjorie.kerzoncuf@ap-hm.fr)[+33491385601](tel:%2B33491385601) PRM

Mrs. Micallef Joelle, Marseille, France, APHM, joelle.micallef@ap-hm.fr +33491387563 Pharmacology

Mr. Blin Olivier, Marseille, France APHM olivier.blin@ap-hm.fr +33491387563 Pharmacology

Mrs Audebert Christine, Marseille, France APHM christine.audebert@ap-hm.fr +33491387563 Pharmacology

Mr Braunstein David, Marseille, France APHM david.braunstein@ap-hm.fr +33491387563 Pharmacology

Mr. Salle Jean-Yves, Limoges, France, CHU Limoges, jean-yves.salle@chu-limoges.fr +33555056516 PRM

Mrs. Laffont Isabelle, Montpellier, France, CHU Montpellier, i-laffont@chu-montpellier.fr +33467332346 PRM

Mr Gélis Anthony, Montpellier, France, Propara, a.gelis@propara.fr +33467046704 PRM

Mr. Djawad Abbas, Marseille, France, C Reeduc Valmante, dabbas@ugecampacac.com 0826961819 PRM

Mrs. Lenne-Aurier Karine, Marseille, France, C Reeduc St Martin karine.aurier@free.fr +334 91 27 30 00 PRM

Mrs. Perrouin-Verbe Brigitte Nantes, France, CHU, brigitte.perrouinverbe@chu-nantes.fr +33240846066 PRM

Mr. Attarian Shahram, Marseille, France, APHM, shahram.attarian@ap-hm.fr +33491386579 Neurology

**SCIENTIFIC DIRECTOR**

Frédéric Brocard (Ph.D.), INT, Faculté de Médecine, Marseille, [frederic.brocard@univ-amu.fr](mailto:frederic.brocard@univ-amu.fr), +33491324029

**ANALYTICS AND PHARMACOKINETICS**

Joseph Ciccolini, Marseille, France, APHM, [joseph.ciccolini@ap-hm.fr](mailto:joseph.ciccolini@ap-hm.fr), +33491387565

Amélie Marsot, Marseille, France, APHM, [amelie.marsot@ap-hm.fr](mailto:amelie.marsot@ap-hm.fr), +33491387565

Romain Guilhaumou, Marseille, France, APHM, [romain.guilhaumou@ap-hm.fr](mailto:romain.guilhaumou@ap-hm.fr), +33491387565

Julien Dupouey, Marseille, France, APHM, [julien.dupouey@ap-hm.fr](mailto:julien.dupouey@ap-hm.fr), +33491387565

**PHARMACY**

Pr Stéphane HONORE, Dr Anita COHEN (UEPRB), AP-HM, anita.cohen@ap-hm.f, +33491387065

**STATISTICS**

Corinne Alberti, F-CRIN Platform Partners, Paris, France, APHP, [Corinne.alberti@inserm.fr](mailto:Corinne.alberti@inserm.fr), +33140032465

Elisabeth Jouve, Marseille, France, APHM, [elisabeth.jouve@ap-hm.fr](mailto:elisabeth.jouve@ap-hm.fr), +33491387563

Romain Truillet, Marseille, France, APHM, [romain.truillet@ap-hm.fr](mailto:romain.truillet@ap-hm.fr), +33491387563

**QUALITY ASSURANCE**

Laurence Attolini, Marseille, France, APHM, [laurence.attolini@ap-hm.fr](mailto:laurence.attolini@ap-hm.fr), +33491387563

**PROJECT MANAGER and QUALITY CONTROL**

DRCI, Marseille, France, APHM, [drci@ap-hm.fr](mailto:drci@ap-hm.fr), +334913827

## Echelles

### Score d’Ashworth modifié

MAS /5

0 Pas d’hypertonie

1 Légère hypertonie avec stretch reflex ou minime résistance en fin de course

2 Hypertonie avec stretch reflex et résistance au cours de la première moitié de la course musculaire autorisée

3 Augmentation importante du tonus musculaire durant toute la course musculaire, mais le segment de membre reste facilement mobilisable

4 Augmentation considérable du tonus musculaire. Le mouvement passif est difficile

5 Hypertonie majeure. Mouvement passif impossible

*Ashworth et al, 1964*

*Bonhannon et al, 1987*

### NRS spasticity

Sur une échelle de 0 à 10, indiquez votre niveau de spasticité au cours de 24 dernières heures.


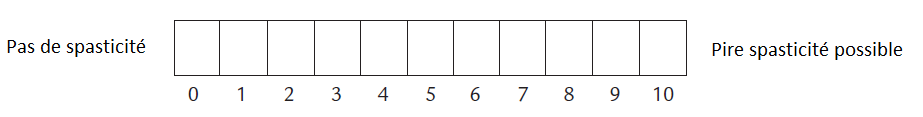


*Commentaires :*

*La spasticité est définie au patient comme la rigidité musculaire ressentie.*

*Farrar et al, 2008*

### Patient global impression to change

Demander au patient d'évaluer la variation globale de son état depuis l'entrée dans l'étude en utilisant l'échelle ci-dessous.

Demander au patient d'enregistrer sa réponse en cochant 1 seule case.


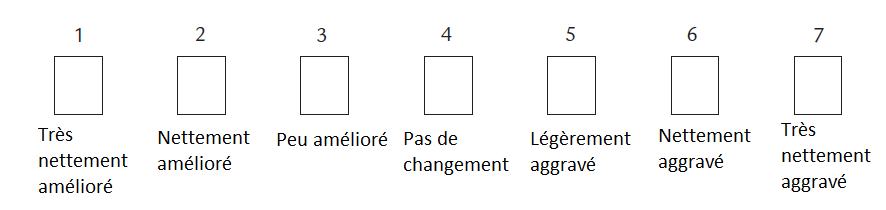


*Farrar et al, 2008*

### Echelle de Penn

0 absence de spasme

1 absence de spasme spontané : présence de spasmes induits par stimulation sensorielle ou mobilisation passive

2 spasmes spontanés occasionnels

3 nombre de spasmes spontanés compris entre 1 et 10 par heure

4 plus de 10 spasmes spontanés par heure

*Penn R. D., 1989*

### EVA Douleur

**
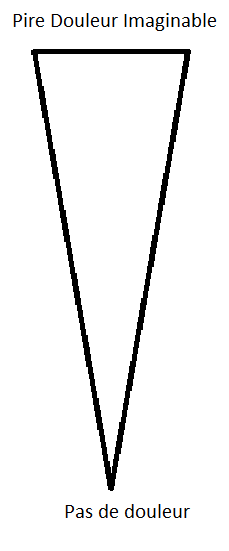
**

### Neuropathic Pain Symptom Inventory

*Vous souffrez de douleurs secondaires à une lésion du système nerveux. Ces douleurs peuvent être de plusieurs types. Il existe des douleurs spontanées, c’est-à-dire des douleurs présentes en l’absence de toute stimulation, qui peuvent être durables ou apparaitre sous forme de crises douloureuses brèves. Il existe également des douleurs provoquées par diverses stimulations (frottement, pression, contact avec le froid). Vous pouvez ressentir un ou plusieurs types de douleur. Le questionnaire que vous allez remplir a été conçu pour permettre à votre médecin de mieux connaitre les différents types de douleurs dont vous souffrez, afin de mieux adapter votre traitement.*

*Nous voudrions savoir si vous avez des douleurs spontanées, c’est-à-dire des douleurs en l’absence de toute stimulation. Pour chacune des questions suivantes, entourez le chiffre qui correspond le mieux à l’intensité de vos douleurs spontanées en moyenne au cours des 24 dernières heures. Entourez le chiffre 0 si vous n’avez pas ressenti ce type de douleur (Veuillez n’entourer qu’un seul chiffre).*

Q1/ Votre douleur est-elle comme une brûlure?


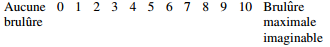


Q2/ Votre douleur est‐elle comme un étau?


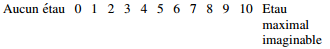


Q3/ Votre douleur est-elle comme une compression?


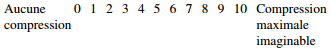


Q4/ **Au cours des dernières 24 heures**, vos douleurs spontanées ont été présentes:

*Veuillez cocher la réponse qui correspond le mieux à votre état :*

En permanence

Entre 8 et 12 heures/jour

Entre 4 et 7 heures/jour

Entre 1 et 3 heures/jour

Moins de 1heure/jour

*Nous voudrions savoir si vous avez des crises douloureuses brèves. Pour chacune des questions suivantes, entourez le chiffre qui correspond le mieux à l’intensité de vos crises douloureuses en moyenne au cours des 24 dernières heures. Entourez le chiffre 0 si vous n’avez pas ressenti ce type de douleur (Veuillez n’entourer qu’un seul chiffre).*

Q5/ Avez‐vous des crises douloureuses comme des décharges électriques?


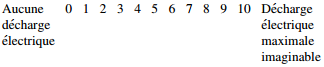


Q6/ Avez-vous des crises douloureuses comme des coups de couteau?


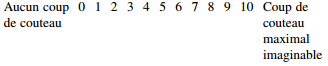


Q7/ **Au cours des dernières 24 heures**, combien de crises douloureuses avez‐vous présenté?

*Veuillez cocher la réponse qui correspond le mieux à votre état :*

Plus de 20

Entre 11 et 20

Entre 6 et 10

Entre 1 et 5

Pas de crise douloureuse

*Nous voudrions savoir si vous avez des douleurs provoquées ou augmentées par le frottement, la pression, le contact d’objets froids sur la zone douloureuse. Pour chacune des questions suivantes, entourez le chiffre qui correspond le mieux à l’intensité de vos douleurs provoquées en moyenne au cours des 24 dernières heures. Entourez le chiffre 0 si vous n’avez pas ressenti ce type de douleur (Veuillez n’entourer qu’un seul chiffre).*

Q8/ Avez-vous des douleurs provoquées ou augmentées par le frottement sur la zone douloureuse?


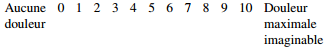


Q9/ Avez‐vous des douleurs provoquées ou augmentées par la pression sur la zone douloureuse?


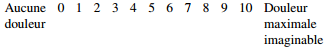


Q10/ Avez-vous des douleurs provoquées ou augmentées par le contact avec un objet froid sur la zone douloureuse?


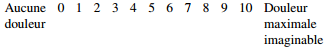


*Nous voudrions savoir si vous avez des sensations anormales dans la zone douloureuse. Pour chacune des questions suivantes, entourez le chiffre qui correspond le mieux à l’intensité de vos sensations anormales en moyenne au cours des 24 dernières heures. Entourez le chiffre 0 si vous n’avez pas ressenti ce type de sensation (Veuillez n’entourer qu’un seul chiffre).*

Q11/ Avez-vous des picotements?


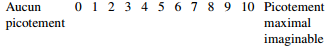


Q12/ Avez‐vous des fourmillements?


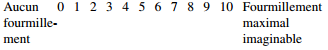


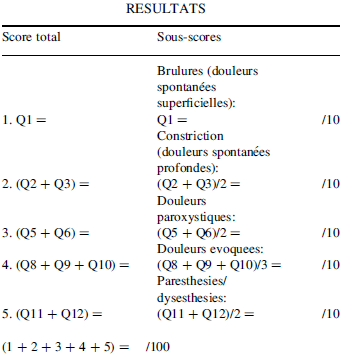


*Bouhassira et al, 2004*

### International Spinal Cord Injury Pain Basic Data Set (ISCIPBDS)

Date :

**Avez-vous eu des douleurs pendant les sept derniers jours, y compris aujourd'hui ?**

Oui Non

Si oui :

Noter que la période de temps des sept derniers jours s’applique à toutes les questions d'interférence de la douleur.

**En général, quelle est l’interférence de la douleur avec vos activités de la vie quotidienne la semaine dernière?**

Pas d’interférence 1 – 2 – 3 – 4 – 5 – 6 – 7 – 8 – 9 – 10 Interférence extrême

**En général, quelle est l’interférence de la douleur avec votre moral la semaine dernière?**

Pas d’interférence 1 – 2 – 3 – 4 – 5 – 6 – 7 – 8 – 9 – 10 Interférence extrême

**En général, quelle est l’interférence de la douleur avec votre capacité à obtenir le sommeil d'une bonne nuit?**

Pas d’interférence 1 – 2 – 3 – 4 – 5 – 6 – 7 – 8 – 9 – 10 Interférence extrême

**Combien de différents problèmes de douleur avez-vous?**

1. ; 2 ; 3 ; 4 ; ≥5

Décrire vos trois principales douleurs :

**
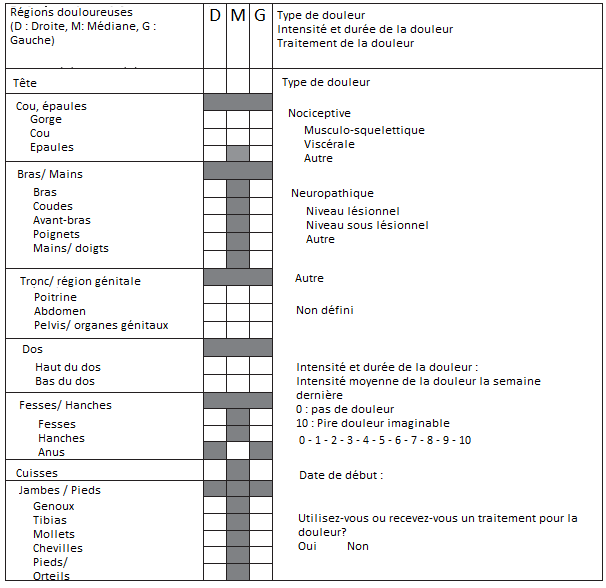
**

**2.**

**
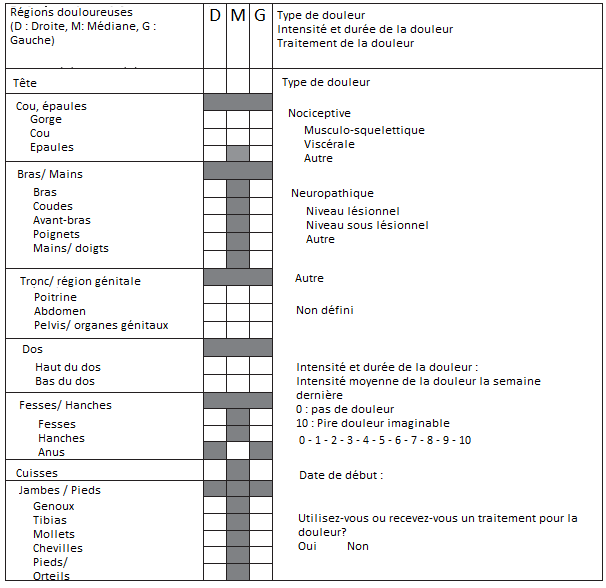
**

**3.**

**
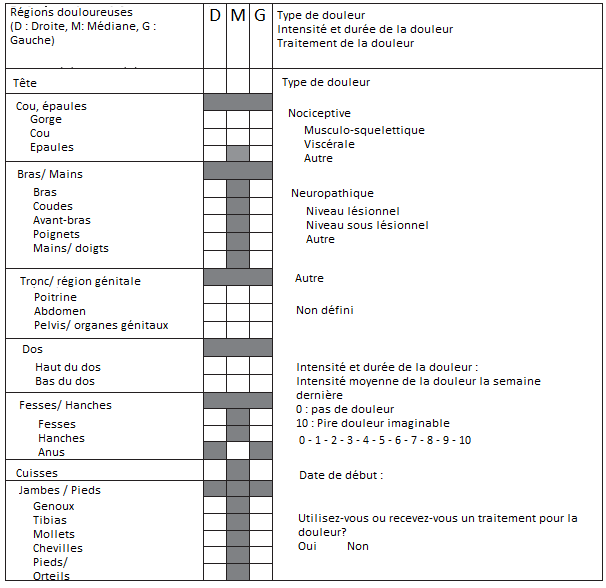
**

### Spinal Cord Independence Measure

*Commentaires :*

*La Spinal Cord Independence Measure (SCIM) s’adresse aussi bien au paraplégique qu’au tétraplégique. Elle couvre 4 domaines fonctionnels : les soins personnels, la respiration, le contrôle sphinctérien et la mobilité. Elle est composée de 16 rubriques. Le score minimum est de 0 et le score maximum de 100.*

**Date**

**SOINS PERSONNELS**

**1. Alimentation (couper la viande, ouvrir une boîte, tenir un gobelet plein, verser du liquide, porter les aliments à la bouche)**

0 Nutrition parentérale, gastrotomie ou assistance totale pour alimentation orale

1 Assistance partielle pour manger et/ou boire, ou pour aliments coupés, assiette et couverts adaptés, incapable de tenir un gobelet

2 Indépendant pour manger, besoin d'AT ou assistance seulement pour couper les aliments et/ou verser et/ou ouvrir une boite)

3 Indépendant dans toutes les tâches sans assistance ou AT

**2. Toilette (utiliser le savon, manipuler les robinets, se laver, se sécher le corps et la tête)**

**A. Partie supérieure du corps**

0 Assistance totale

1 Assistance partielle

2 Indépendant avec AT ou installation spéciale

3 Indépendant sans AT ni installation spéciale

**B. Partie inférieure du corps**

0 Assistance totale

1 Assistance partielle

2 Indépendant avec AT ou installation spéciale

3 Indépendant sans AT ni installation spéciale

**3. Habillage (préparation des habits, habillage, déshabillage, chaussage, mise en place des orthèses permanentes)**

**A. Partie supérieure du corps**

0 Assistance totale

1 Assistance partielle pour avec les vêtements sans boutons, fermetures éclairs ou lacets (vsbfl)

2 Indépendant pour vsbfl ; besoin AT et/ ou installation spéciale

3 Indépendant pour vsbfl ; pas besoin AT et/ou installation spéciale sauf pour bfl

4 Indépendant (pour tout type de vêtement) sans AT et/ ou installation spéciale

**B. Partie inférieure du corps**

0 Assistance totale

1 Assistance partielle pour avec les vêtements sans boutons, fermetures éclairs ou lacets (vsbfl)

2 Indépendant pour vsbfl ; besoin AT et/ ou installation spéciale

3 Indépendant pour vsbfl ; pas besoin AT et/ou installation spéciale sauf pour bfl

4 Indépendant (pour tout type de vêtement) sans AT et/ou installation spéciale

**4. Soins d'apparence (se laver les mains et le visage, se coiffer, brossage des dents, rasage, maquillage)**

0 Assistance totale

1 Assistance partielle

2 Indépendant avec AT

3 Indépendant sans AT

**Sous total (0-20)**

**RESPIRATION ET CONTROLE SPHINCTÉRIEN**

**5. Respiration**

0 Sonde trachéale (ST) et ventilation assistée (VA) permanente ou intermittente

2 Respire spontanément avec ST; besoin oxygène, assistance pour tousser et soins trachéaux

4 Respire spontanément avec ST + peu d'assistance pour tousser ou soins trachéaux

6 Respire spontanément sans ST + besoin d'oxygène et soins importants pour tousser, un masque ou VA

8 Respire sans ST; besoin d'un peu d'assistance mécanique pour tousser

10 Respiration normale sans aide ou AT

**6. Contrôle vésico-sphinctérien - Vessie**

0 Sonde urinaire à demeure

3 Résidu post mictionnel (RPM) > 100 cc, pas de sonde, pas de SI

6 RPM <100 cc, ou auto sondages intermittents, aide nécessaire pour vidange vésicale

9 Auto sondages intermittents < 100 cc, utilise une AT pour vidange vésicale sans assistance

11 Auto sondages intermittents, continent entre les sondages, sans AT

13 RPM < 100 cc, vidange vésicale externe uniquement sans aide

15 RPM < 100 cc, totalement continent sans vidange vésicale

**7. Contrôle sphincter anal**

0 Évacuation des selles inappropriées, ou irrégulières, ou fréquence < à 1 fois/3j

5 Évacuation régulière et adaptée avec assistance (ex : mise du suppo), rares fuites (< 1 fois/mois)

8 Évacuation régulière et adaptée sans assistance, rares fuites (< 1 fois/mois)

10 Évacuation régulière sans assistance pas d'accidents

**8. Utilisation des toilettes (hygiène périnéale, déshabillage, rhabillage, utilisation de couches ou de serviettes périodiques)**

0 Besoin d'assistance totale

1 Assistance partielle, ne peut se laver seul

2 Assistance partielle, peut se laver seul

4 Indépendant dans toutes les tâches, nécessite AT ou installation spéciale

5 Indépendant sans AT ni installation spéciale

**Sous total (0-40)**

**MOBILITÉ (Chambre et Toilettes)**

**9. Mobilité dans le lit et prévention des points d'appui**

0 Besoin d'assistance totale dans toutes les activités : tourner le haut et bas du corps dans le lit, s'asseoir, push-up en fauteuil, avec ou sans AT, mais sans aides électriques

2 Peut accomplir une de ces activités sans aide

4 Peut accomplir deux ou trois activités sans aide

6 Totalement indépendant pour toutes les activités de mobilité dans le lit et prévention des points d'appui

**10. Transferts lit-fauteuil roulant (bloquer le fauteuil, soulever les appuis-pieds, enlever et ajuster les repose-bras, transfert, lever les pieds)**

0 Besoin d'assistance totale

1 Besoin d'assistance partielle et/ou surveillance et/ou AT (ex : planche de transfert)

2 Indépendant (ou n'a pas besoin de fauteuil roulant)

**11. Transferts fauteuil roulant-W.C. (Bloquer le fauteuil, soulever les appuis-pieds, enlever et ajuster les repose-bras, transfert, lever les pieds)**

0 Besoin d'assistance totale

1 Besoin assistance partielle et/ou surveillance ou aménagement (ex : barre d'appui)

2 Indépendant (ou n'a pas besoin de fauteuil roulant)

**DÉPLACEMENTS (à l'intérieur et à l'extérieur, sur surfaces planes)**

**12. Déplacements à l'intérieur (courtes distances)**

0 Assistance totale

1 A besoin d'un FRE ou d'une aide pour déplacer le FRM

2 Se déplace seul avec un FRM

3 Surveillance pour la marche (avec ou sans AT)

4 Marche avec déambulateur ou cannes anglaises (swing)

5 Marche avec 2 cannes anglaises ou cannes simples (marche réciproque)

6 Marche avec 1 canne simple

7 Utilise seulement une orthèse

8 Marche sans AT

**13. Déplacements sur distances moyennes (10 - 100 m)**

0 Assistance totale

1 A besoin d'un FRE ou d'une aide pour déplacer le FRM

2 Se déplace seul avec un FRM

3 Surveillance pour la marche (avec ou sans AT)

4 Marche avec déambulateur ou cannes anglaises (swing)

5 Marche avec 2 cannes anglaises ou cannes simples (marche réciproque)

6 Marche avec 1 canne simple

7 Utilise seulement une orthèse

8 Marche sans AT

**14. Déplacements à l'extérieur (> 100 m)**

0 Assistance totale

1 A besoin d'un FRE ou d'une aide pour déplacer le FRM

2 Se déplace seul avec un FRM

3 Surveillance pour la marche (avec ou sans AT)

4 Marche avec déambulateur ou cannes anglaises (swing)

5 Marche avec 2 cannes anglaises ou cannes simples (marche réciproque)

6 Marche avec 1 canne simple

7 Utilise seulement une orthèse

8 Marche sans AT

**15. Escaliers**

0 Incapable de monter ou descendre des escaliers

1 Monte et descend au moins 3 marches avec aide ou surveillance d'un tiers

2 Monte et descend au moins 3 marches avec appui d'une rampe et / ou canne

3 Monte et descend au moins 3 marches sans aucun appui ni surveillance

**16. Transferts fauteuil roulant - voiture (accéder à la voiture, bloquer le fauteuil roulant, enlever les appuis-pieds et repose-bras, transfert fauteuil roulant-voiture, mettre/sortir le fauteuil roulant)**

0 Besoin d'assistance totale

1 Besoin d'assistance partielle et / ou surveillance et / ou aide technique

2 Indépendant sans aide technique

**16. Transferts fauteuil roulant-sol**

0 Besoin d'assistance totale

1 Indépendant pour les transferts avec ou sans AT

**Sous total (0-40)**

**TOTAL (sur 100)**

*AT : aide technique FRM : Fauteuil roulant manuel FRE : Fauteuil roulant électrique*

*RPM : résidu post-mictionnel*

*Itzkovich M. et al, 2007*

*Version traduite en langue française (Bénédicte Clément CMN Propara)*

### ASIA


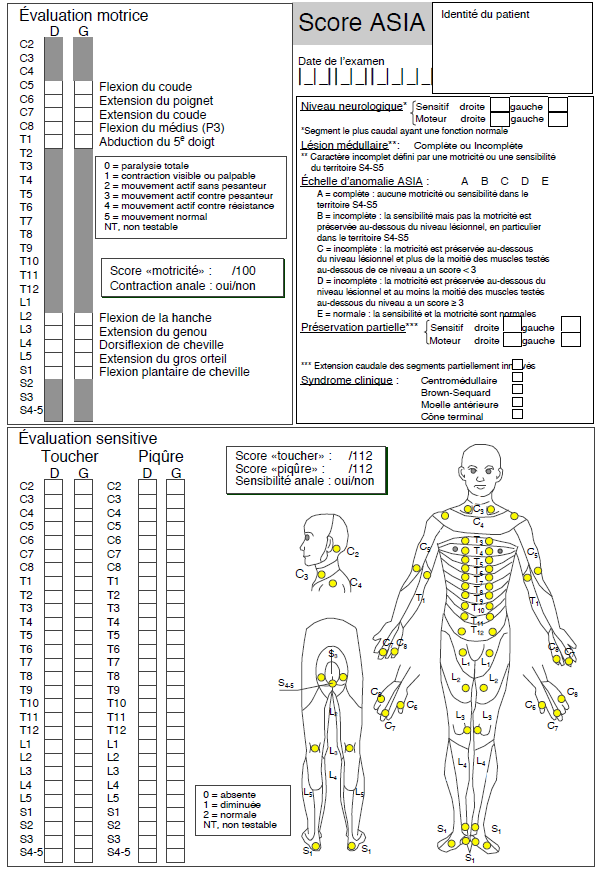


*American Spinal Cord Injury/ ISCOS*

### DN4

Pour estimer la probabilité d’une douleur neuropathique, le patient doit répondre à chaque item des 4 questions ci-dessous par « oui » ou « non ».

QUESTION 1 La douleur présente-t-elle une ou plusieurs des caractéristiques suivantes?

|  | **Oui** | **Non** |
| --- | --- | --- |
| **1.** Brûlure |  |  |
| **2.** Sensation de froid douloureux |  |  |
| **3.** Décharges électriques |  |  |

QUESTION 2 La douleur est-elle associée dans la même région à un ou plusieurs des symptômes suivants ?

|  | **Oui** | **Non** |
| --- | --- | --- |
| **4**. Fourmillements |  |  |
| **5.** Picotements |  |  |
| **6.** Engourdissements |  |  |
| **7.** Démangeaisons |  |  |

QUESTION 3 La douleur est-elle localisée dans un territoire où l’examen met en évidence?

|  | **Oui** | **Non** |
| --- | --- | --- |
| **8.** Hypoesthésie au tact |  |  |
| **9.** Hypoesthésie à la piqûre |  |  |

QUESTION 4 La douleur est-elle provoquée ou augmentée par :

|  | **Oui** | **Non** |
| --- | --- | --- |
| **10.** Le frottement |  |  |

**Score /10**

***Mode d’emploi***

*Lorsque le praticien suspect une douleur neuropathique, le questionnaire DN4 est utile comme outil de diagnostic.*

*Ce questionnaire se répartit en 4 questions représentant 10 items à cocher :*

*- le praticien interroge lui-même le patient et remplit le questionnaire ;*

*- à chaque item, il doit apporter une réponse « oui » ou « non » ;*

*- à la fin du questionnaire, le praticien comptabilise les réponses, 1 pour chaque« oui » et 0 pour chaque « non » ;*

*- la somme obtenue donne le Score du patient, noté sur 10.*

*Si le score du patient est égal ou supérieur à 4, le test est positif.*

***(Sensibilité de 82,9 %; spécificité à 89,9 %)***

*Kuntzer T, Decosterd I, 2005.*

# BIBLIOGRAPHY

1. Adams M.M. and Hicks A.L. (2005). Spasticity after spinal cord injury. Spinal Cord. 43, 577-586.
2. Agrawal S.K. and Fehlings M.G. (1996). Mechanisms of secondary injury to spinal cord axons in vitro: role of Na+, Na(+)-K(+)-ATPase, the Na(+)-H+ exchanger, and the Na(+)-Ca2+ exchanger. J. Neurosci. 16, 545-552.
3. Albright A.L. and Shultz B.L. (1999). Plasma baclofen levels in children receiving continuous intrathecal baclofen infusion. J Child Neurol 14, 408-409.
4. Ashworth B. (1964). Preliminary trial of carisoprodol in multiple sclerosis. Practitioner 192, 540-542.
5. Ates O., Cayli S.R., Gurses I., Turkoz Y., Tarim O., Cakir C.O., and Kocak A. (2007). Comparative neuroprotective effect of sodium channel blockers after experimental spinal cord injury. J. Clin. Neurosci. 14, 658-665.
6. Baastrup C. and Finnerup N.B. (2008). Pharmacological management of neuropathic pain following spinal cord injury. CNS. Drugs 22, 455-475.
7. Bennett D.J., Li Y., Harvey P.J., and Gorassini M. (2001). Evidence for plateau potentials in tail motoneurons of awake chronic spinal rats with spasticity. J. Neurophysiol. 86, 1972-1982.
8. Bennett M.I., Smith B.H., Torrance N., and Potter J. (2005). The S-LANSS score for identifying pain of predominantly neuropathic origin: validation for use in clinical and postal research. J. Pain 6, 149-158.
9. Bensimon G. and Doble A. (2004). The tolerability of riluzole in the treatment of patients with amyotrophic lateral sclerosis. Expert Opin. Drug Saf 3, 525-534.
10. Bensimon G., Lacomblez L., and Meininger V. (1994). A controlled trial of riluzole in amyotrophic lateral sclerosis. ALS/Riluzole Study Group. N. Engl. J. Med. 330, 585-591.
11. Bogdanov EI . Spinal Injury. Lisak RP, Truong DD Carroll WM Bhidayasiri R. International Neurology: A Clinical Approach . 2014. Blackwell Publishing.
12. Bohannon R.W. and Smith M.B. (1987). Interrater reliability of a modified Ashworth scale of muscle spasticity. Phys. Ther. 67, 206-207.
13. Bouhadfane M., Tazerart S., Moqrich A., Vinay L., and Brocard F. (2013). Sodium-mediated plateau potentials in lumbar motoneurons of neonatal rats. J Neurosci. 33, 15626-15641.
14. Bouhassira D, Attal N, Fermanian J, Alchaar H, Gautron M, Masquelier E, Rostaing S, Lanteri-Minet M, Collin E, Grisart J, Boureau F (2004). Development and validation of the Neuropathic Pain Symptom Inventory. Pain, Apr;108(3):248-57
15. Boulenguez P., Liabeuf S., Bos R., Bras H., Jean-Xavier C., Brocard C., Stil A., Darbon P., Cattaert D., Delpire E., Marsala M., and Vinay L. (2010). Down-regulation of the potassium-chloride cotransporter KCC2 contributes to spasticity after spinal cord injury. Nat. Med. 16, 302-307.
16. Boulenguez P. and Vinay L. (2009). Strategies to restore motor functions after spinal cord injury. Curr. Opin. Neurobiol. 19, 587-600.
17. Brocard F., Shevtsova N.A., Bouhadfane M., Tazerart S., Heinemann U., Rybak I.A., and Vinay L. (2013). Activity-dependent changes in extracellular Ca2+ and K+ reveal pacemakers in the spinal locomotor-related network. Neuron 77, 1047-1054.
18. Bruno R., Vivier N., Montay G., Le L.A., Powe L.K., Delumeau J.C., and Rhodes G.R. (1997). Population pharmacokinetics of riluzole in patients with amyotrophic lateral sclerosis. Clin. Pharmacol. Ther. 62, 518-526.
19. Burchiel K.J. and Hsu F.P. (2001). Pain and spasticity after spinal cord injury: mechanisms and treatment. Spine (Phila Pa 1976. ) 26, S146-S160.
20. Catz A. and Itzkovich M. (2007). Spinal Cord Independence Measure: comprehensive ability rating scale for the spinal cord lesion patient. J Rehabil Res. Dev. 44, 65-68.
21. Chew DJ, Carlstedt T, Shortland PJ (2014). The effects of minocycline or riluzole treatment on spinal root avulsion-induced pain in adult rats. J Pain. Jun;15(6):664-75
22. Chow D.S., Teng Y., Toups E.G., Aarabi B., Harrop J.S., Shaffrey C.I., Johnson M.M., Boakye M., Frankowski R.F., Fehlings M.G., and Grossman R.G. (2012). Pharmacology of riluzole in acute spinal cord injury. J. Neurosurg. Spine 17, 129-140.
23. Cifra A., Mazzone G.L., and Nistri A. (2013). Riluzole: what it does to spinal and brainstem neurons and how it does it. Neuroscientist. 19, 137-144.
24. Cleeland CS (1989). Measurement of pain by subjective report. In: Chapman CR, Loeser JD, editors. Advances in Pain Research and Therapy, Volume 12: Issues in Pain Measurement. New York: Raven Press; pp. 391-403.
25. Decq P. (2003). [Pathophysiology of spasticity]. Neurochirurgie 49, 163-184.
26. Eken T., Hultborn H., and Kiehn O. (1989). Possible functions of transmitter-controlled plateau potentials in alpha motoneurones. Prog. Brain Res. 80, 257-267.
27. Estevez A.G., Stutzmann J.M., and Barbeito L. (1995). Protective effect of riluzole on excitatory amino acid-mediated neurotoxicity in motoneuron-enriched cultures. Eur. J. Pharmacol. 280, 47-53.
28. Farrar J.T., Troxel A.B., Stott C., Duncombe P. and Jensen M.P. (2008). Validity, reliability, and clinical importance of change in a 0-10 numeric rating scale measure of spasticity: a post hoc analysis of a randomized, double-blind, placebo-controlled trial. Clin. Ther. 30, 974-985.
29. Fehlings M.G., Wilson J.R., Frankowski R.F., Toups E.G., Aarabi B., Harrop J.S., Shaffrey C.I., Harkema S.J., Guest J.D., Tator C.H., Burau K.D., Johnson M.W. and Grossman R.G. (2012). Riluzole for the treatment of acute traumatic spinal cord injury: rationale for and design of the NACTN Phase I clinical trial. J. Neurosurg. Spine 17, 151-156.
30. Furlan J.C., Sakakibara B.M., Miller W.C. and Krassioukov A.V. (2013). Global incidence and prevalence of traumatic spinal cord injury. Can. J Neurol Sci. 40, 456-464.
31. Gorassini M.A., Knash M.E., Harvey P.J., Bennett D.J. and Yang,J.F. (2004). Role of motoneurons in the generation of muscle spasms after spinal cord injury. Brain 127, 2247-2258.
32. Gorassini M.A., Norton J.A., Nevett-Duchcherer J., Roy F.D. and YangJ.F. (2009). Changes in locomotor muscle activity after treadmill training in subjects with incomplete spinal cord injury. J. Neurophysiol. 101, 969-979.
33. Grossman R.G., Fehlings M.G., Frankowski R.F., Burau K.D., Chow D.S., Tator C., Teng A., Toups E.G., Harrop J.S., Aarabi B., Shaffrey C.I., Johnson M.M., Harkema S.J., Boakye M., Guest J.D. and Wilson J.R. (2013). A Prospective, Multicenter, Phase I Matched-Comparison Group Trial of Safety, Pharmacokinetics, and Preliminary Efficacy of Riluzole in Patients with Traumatic Spinal Cord Injury. J. Neurotrauma.
34. Haanpaa M., Attal N., Backonja M., Baron R., Bennett M., Bouhassira D., Cruccu G., Hansson P., Haythornthwaite J.A., Iannetti G.D., Jensen T.S., Kauppila T., Nurmikko T.J., Rice A.S., Rowbotham M., Serra J., Sommer C., Smith B.H. and Treede R.D. (2011). NeuPSIG guidelines on neuropathic pain assessment. Pain 152, 14-27.
35. Hains B.C., Klein J.P., Saab C.Y., Craner M.J., Black J.A. and Waxman,S.G. (2003). Upregulation of sodium channel Nav1.3 and functional involvement in neuronal hyperexcitability associated with central neuropathic pain after spinal cord injury. J. Neurosci. 23, 8881-8892.
36. Hama A. and Sagen J. (2011). Antinociceptive effect of riluzole in rats with neuropathic spinal cord injury pain. J. Neurotrauma 28, 127-134.
37. Harvey P.J., Li X., Li Y., and Bennett D.J. (2006). 5-HT2 receptor activation facilitates a persistent sodium current and repetitive firing in spinal motoneurons of rats with and without chronic spinal cord injury. J Neurophysiol. 96, 1158-1170.
38. Haute Autorité de Santé. Référentiel d'auto-évaluations des pratiques professionnelles en massokinésithérapie. 2006.

Ref Type: Generic

1. Heckman C.J., Johnson M., Mottram C. and Schuster J. (2008). Persistent inward currents in spinal motoneurons and their influence on human motoneuron firing patterns. Neuroscientist. 14, 264-275.
2. Heurteaux C., Laigle C., Blondeau N., Jarretou G. and Lazdunski M. (2006). Alpha-linolenic acid and riluzole treatment confer cerebral protection and improve survival after focal brain ischemia. Neuroscience 137, 241-251.
3. Jayaraman A., Gregory C.M., Bowden M., Stevens J.E., Shah P., Behrman A.L. and Vandenborne K. (2006). Lower extremity skeletal muscle function in persons with incomplete spinal cord injury. Spinal Cord. 44, 680-687.
4. Jozefczyk P.B. (2002). The management of focal spasticity. Clin. Neuropharmacol. 25, 158-173.
5. Kirshblum S. (1999). Treatment alternatives for spinal cord injury related spasticity. J Spinal Cord Med. 22, 199-217.
6. Lacomblez L., Bensimon G., Leigh P.N., Guillet P., Powe L., Durrleman S., Delumeau J.C., and Meininger V. (1996). A confirmatory dose-ranging study of riluzole in ALS. ALS/Riluzole Study Group-II. Neurology 47, S242-S250.
7. Lampert A., Hains B.C. and Waxman S.G. (2006). Upregulation of persistent and ramp sodium current in dorsal horn neurons after spinal cord injury. Exp. Brain Res. 174, 660-666.
8. Lance J.W. (1980). The control of muscle tone, reflexes, and movement: Robert Wartenberg Lecture. Neurology 30, 1303-1313.
9. Lang-Lazdunski L., Heurteaux C., Vaillant N., Widmann C. and Lazdunski M. (1999). Riluzole prevents ischemic spinal cord injury caused by aortic crossclamping. J Thorac. Cardiovasc. Surg. 117, 881-889.
10. Li Y. and Bennett D.J. (2003). Persistent sodium and calcium currents cause plateau potentials in motoneurons of chronic spinal rats. J Neurophysiol. 90, 857-869.
11. Maynard F.M., Karunas R.S. and Waring W.P., III (1990). Epidemiology of spasticity following traumatic spinal cord injury. Arch. Phys. Med. Rehabil. 71, 566-569.
12. Merskey H. and Bogduk N. (1986). Classification of chronic pain. Descriptions of chronic pain syndromes and definitions of pain terms. Prepared by the International Association for the Study of Pain, Subcommittee on Taxonomy. Pain Suppl 3, S1-226.
13. Moon ES, Karadimas SK, Yu WR, Austin JW, Fehlings MG (2014). Riluzole attenuates neuropathic pain and enhances functional recovery in a rodent model of cervical spondylotic myelopathy. Neurobiol Dis. Feb;62:394-406.
14. Morgan P, Van Der Graaf PH, Arrowsmith J, Feltner DE, Drummond KS, Wegner CD, Street SD (2012). Can the flow of medicines be improved? Fundamental pharmacokinetic and pharmacological principles toward improving Phase II survival. Drug Discov Today17(9-10):419-24
15. Nagoshi N, Nakashima H, Fehlings MG (2015). Riluzole as a Neuroprotective Drug for Spinal Cord Injury: From Bench to Bedside. Molecules. Apr 29;20(5):7775-7789
16. Nicholson KJ, Zhang S, Gilliland TM, Winkelstein BA (2014). Riluzole effects on behavioral sensitivity and the development of axonal damage and spinal modifications that occur after painful nerve root compression. J Neurosurg Spine, Jun;20(6):751-62.
17. Novotna A., Mares J., Ratcliffe S., Novakova I., Vachova M., Zapletalova O., Gasperini C., Pozzilli C., Cefaro L., Comi G., Rossi P., Ambler Z., Stelmasiak Z., Erdmann A., Montalban X., Klimek A., and Davies P. (2011). A randomized, double-blind, placebo-controlled, parallel-group, enriched-design study of nabiximols* (Sativex((R)) ), as add-on therapy, in subjects with refractory spasticity caused by multiple sclerosis. Eur. J Neurol 18, 1122-1131.
18. O'Quigley J., Pepe M. and Fisher L. (1990). Continual reassessment method: a practical design for phase 1 clinical trials in cancer. Biometrics 46, 33-48.
19. Parziale J.R., Akelman E. and Herz D.A. (1993). Spasticity: pathophysiology and management. Orthopedics 16, 801-811.
20. Platz T., Eickhof C., Nuyens G. and Vuadens P. (2005). Clinical scales for the assessment of spasticity, associated phenomena, and function: a systematic review of the literature. Disabil. Rehabil 27, 7-18.
21. Powers R.K. and Rymer W.Z. (1988). Effects of acute dorsal spinal hemisection on motoneuron discharge in the medial gastrocnemius of the decerebrate cat. J Neurophysiol. 59, 1540-1556.
22. Resche-Rigon M., Zohar S., and Chevret S. (2008). Adaptive designs for dose-finding in non-cancer phase II trials: influence of early unexpected outcomes. Clin. Trials 5, 595-606.
23. Sadlaoud K., Tazerart S., Brocard C., Jean-Xavier C., Portalier P., Brocard F., Vinay L. and Bras H. (2010). Differential plasticity of the GABAergic and glycinergic synaptic transmission to rat lumbar motoneurons after spinal cord injury. J. Neurosci. 30, 3358-3369.
24. Sallerin B. and Lazorthes Y. (2003). [Intrathecal baclofen. Experimental and pharmacokinetic studies]. Neurochirurgie 49, 271-275.
25. Schwartz G. and Fehlings M.G. (2001). Evaluation of the neuroprotective effects of sodium channel blockers after spinal cord injury: improved behavioral and neuroanatomical recovery with riluzole. J. Neurosurg. 94, 245-256.
26. Sheean G. (2002). The pathophysiology of spasticity. Eur. J Neurol 9 Suppl 1, 3-9.
27. Siddall P.J. and Loeser J.D. (2001). Pain following spinal cord injury. Spinal Cord. 39, 63-73.
28. Siddall P.J., McClelland J.M. Rutkowski S.B., and Cousins M.J. (2003). A longitudinal study of the prevalence and characteristics of pain in the first 5 years following spinal cord injury. Pain 103, 249-257.
29. Siddall P.J., Taylor D.A. and Cousins M.J. (1997). Classification of pain following spinal cord injury. Spinal Cord. 35, 69-75.
30. Skold C. (2000). Spasticity in spinal cord injury: self- and clinically rated intrinsic fluctuations and intervention-induced changes. Arch. Phys. Med. Rehabil. 81, 144-149.
31. Skold C., Levi R. and Seiger A. (1999). Spasticity after traumatic spinal cord injury: nature, severity, and location. Arch. Phys. Med. Rehabil 80, 1548-1557.
32. St George C.L. (1993). Spasticity. Mechanisms and nursing care. Nurs. Clin. North Am. 28, 819-827.
33. Stys P.K., Waxman S.G. and Ransom B.R. (1992). Ionic mechanisms of anoxic injury in mammalian CNS white matter: role of Na+ channels and Na(+)-Ca2+ exchanger. J. Neurosci. 12, 430-439.
34. Tazerart S., Viemari J.C., Darbon P., Vinay L. and Brocard ,F. (2007). Contribution of persistent sodium current to locomotor pattern generation in neonatal rats. J. Neurophysiol. 98, 613-628.
35. Tazerart S., Vinay L. and Brocard F. (2008). The persistent sodium current generates pacemaker activities in the central pattern generator for locomotion and regulates the locomotor rhythm. J. Neurosci. 28, 8577-8589.
36. Teasell R.W., Mehta S., Aubut J.A., Foulon B., Wolfe D.L., Hsieh J.T., Townson A.F. and Short C. (2010). A systematic review of pharmacologic treatments of pain after spinal cord injury. Arch. Phys. Med. Rehabil. 91, 816-831.
37. Theiss R.D., Hornby T.G., Rymer W.Z., and Schmit B.D. (2011). Riluzole decreases flexion withdrawal reflex but not voluntary ankle torque in human chronic spinal cord injury. J. Neurophysiol. 105, 2781-2790.
38. Van den Berg M.E., Castellote J.M., de Pedro-Cuesta J. and Mahillo-Fernandez I. (2010). Survival after spinal cord injury: a systematic review. J Neurotrauma 27, 1517-1528.
39. Vattanasilp W., Ada L., and Crosbie J. (2000). Contribution of thixotropy, spasticity, and contracture to ankle stiffness after stroke. J Neurol Neurosurg. Psychiatry 69, 34-39.
40. Wang S.J., Wang K.Y., and Wang W.C. (2004). Mechanisms underlying the riluzole inhibition of glutamate release from rat cerebral cortex nerve terminals (synaptosomes). Neuroscience 125, 191-201.
41. Ward A.B. (2003). Long-term modification of spasticity. J Rehabil Med. 60-65.
42. Ward A.B. (2008). Spasticity treatment with botulinum toxins. J Neural Transm. 115, 607-616.
43. Waxman S.G. and Hains B.C. (2006). Fire and phantoms after spinal cord injury: Na+ channels and central pain. Trends Neurosci. 29, 207-215.
44. Wu Y., Satkunendrarajah K., Teng Y., Chow D.S., Buttigieg J. and Fehlings M.G. (2013). Delayed post-injury administration of riluzole is neuroprotective in a preclinical rodent model of cervical spinal cord injury. J. Neurotrauma 30, 441-452.
45. Yelnik A.P., Simon O., Bensmail D., Chaleat-Valayer E., Decq P., Dehail P., Quentin V., Marque P., Parratte B., Pellas F., Rousseaux M., Trocello J.M., Uzzan M. and Dumarcet N. (2009). Drug treatments for spasticity. Ann. Phys. Rehabil. Med. 52, 746-756.
46. Yelnik A.P., Simon O., Parratte B. and Gracies J.M. (2010). How to clinically assess and treat muscle overactivity in spastic paresis. J. Rehabil. Med. 42, 801-807.
47. Yoshimura N. and de Groat W.C. (1997). Plasticity of Na+ channels in afferent neurones innervating rat urinary bladder following spinal cord injury. J Physiol 503 ( Pt 2), 269-276.
48. Zohar S, Chevret S. “Phase I (or phase II) dose-ranging clinical trials: Proposal of a two-stage Bayesian design” Journal of Biopharmaceutical Statistics, 2003 ; 13 : 87-101
49. Zohar S. et al.. Software to compute and conduct sequential Bayesian phase I or II dose-ranging clinical trials with stopping rules. Comput Methods Programs Biomed, 2003. 72(2): p. 117-25.
50. Widerström-Noga E, Biering-Sørensen F, Bryce T, Cardenas DD, Finnerup NB, Jensen MP, Richards JS, Siddall PJ (2008). The international spinal cord injury pain basic data set. Spinal Cord. Dec;46(12):818-23
