# Supplementary material for: Riluzole for treating spasticity in patients with chronic traumatic spinal cord injury: Study protocol in the phase ib/iib adaptive multicenter randomized controlled RILUSCI trial
Source: PLoS One. 2023 Jan 20;18(1):e0276892. doi: 10.1371/journal.pone.0276892 (PMC9858801; doi:10.1371/journal.pone.0276892)
Supplement: S4 File — (DOCX) [file pone.0276892.s004.docx]

### Newsletter

**"Study of Riluzole in the treatment of spasticity after chronic traumatic spinal cord injury: a double-blind, randomized, placebo-controlled, multicenter, adaptive trial in a rare disease: RILUSCI"**

Sponsor: AP-HM (Assistance Publique des Hôpitaux de Marseille)

Direction de la Recherche Clinique et de l'Innovation - 80 rue Brochier - 13 354 Marseille Cedex 5 Coordinating Investigator: Pr Viton

Madam, Sir,

You suffer from a chronic traumatic spinal cord injury for which your doctor is treating you. He/she suggests that you participate in a biomedical research study for which **Professor Jean Michel Viton** is the coordinating investigator and the AP-HM is the promoter.

The purpose of this newsletter is to give you a detailed description of the study, what it involves, the risks and benefits associated with it, and your rights.

If you have any questions after reading this document, please feel free to ask the study doctor. The information given, how the study will be conducted, and what your role will be should be clear in your mind. If you decide to participate in the study, you will need to initial this information letter and sign the informed consent form confirming that you understand the information provided.

If you have other questions during the study, you can ask the study doctor at any time.

You will be able to give your consent after a reflection period. Even after signing the informed consent form, you will be free to leave the study at any time, without having to explain yourself.

### RATIONALE FOR THE STUDY

Spasticity, which is very common in patients with traumatic spinal cord injury, is a disabling symptom with an impact on mobility, hygiene and activities of daily living.

The current therapeutic arsenal for the reduction of spasticity consists of oral benzodiazepines, oral or intrathecal Baclofen, oral Dantrolene, or local botulinum toxin.

Despite the marketing authorization of several pharmaceutical products for the treatment of spasticity and pain related to traumatic spinal cord injury, their relative efficacy reported by healthcare professionals and patients underscores the need to broaden the range of therapeutic options.

Riluzole has a Marketing Authorization for the treatment of Amyotrophic Lateral Sclerosis (ALS). Daily treatment with Riluzole 100 mg/d has been shown to improve life expectancy in ALS patients.

Studies conducted in animals with this drug have shown a reduction in spasticity. Studies are underway in humans in this pathology.

Given the inter-individual variability of Riluzole blood levels, it is necessary in a first step (step 1 of the study) to determine the minimal effective dose (MED) to improve spasticity.

In step 2 of the study, we will investigate the efficacy of Riluzole on spasticity and neuropathic pain in the chronic phase of traumatic spinal cord injury (SCI). We will use two scales (Ashworth scale and a numerical scale, rated from to0 self-report by10, the patient) to assess spasticity from the patient's perspective and from the physician's perspective before and after treatment.

### PURPOSE OF THE STUDY

This study is a multicenter study, 7French centers will participate.

It will include gender2 patients90 with chronic traumatic spinal cord injury (SCI) and will be conducted in two successive stages. Patients30 will be included in the first stage and then in 60the second.

The first step of the study (step 1) will be to determine the Minimum Effective Dose (MED) from the doses 4studied. The second stage of the study (stage 2) will estimate the probability of response with the dose determined in the first study.

The main objective of step 1 is to determine the daily dose of Riluzole that will result in an improvement of spasticity in 75% of treated patients, as judged by your doctor using 2 clinical assessment scales.

The primary objective of this step is2 to demonstrate the efficacy of Riluzole, administered at the Minimum Effective Dose determined in step 1, compared to placebo in improving spasticity in these patients.

The secondary objectives of this study will be multiple: Evaluation of the safety of Riluzole in IBS patients, evaluation of the effects of Riluzole on daily activity, pain and bladder problems in IBS patients, and determination of the pharmacokinetics i.e. the study of Riluzole blood concentrations.

The placebo resembles the study drug in every way (appearance, taste) but it has no pharmacological activity because it does not contain active ingredients. In trials, the use of a placebo to treat a portion of the subjects under double-blind conditions makes it possible to compare this group with those receiving the active product in order to objectively evaluate the real effectiveness of the experimental treatment. During this study, neither you nor the study doctor and his team will know the exact nature of the treatment you will receive (double-blind study). However, in case of an emergency, for your safety, if the doctor needs to know the exact nature of the treatment you will receive, he or she will be able to receive this information quickly.

### STUDY PLAN

The duration of participation for each stage (stage 1 of the study and stage 2 of the study) will be a maximum of 5 weeks and will include the screening visit within 15 days prior to inclusion, the inclusion visit with assignment of study treatment, taking treatment for 14 days on an outpatient basis, an intermediate visit 3 days after the start of treatment, a visit on the last day of treatment, and the follow- up visit one week later.

Your participation can only be voluntary. If you agree to participate in the study, you will be invited to the screening visit in the hospital department.

### o Patient participating in the step1:

Patients will be included in groups of 2 and will receive one of four doses of Riluzole: 50, 100, 150 or 200mg/d. The first 2 patients included in the study will receive a randomly assigned dose and then all patients2 will be analyzed for efficacy on spasticity and the choice of the next dose will be determined based on the result. The treatment, in the form of capsules2 per dose, will be administered twice a day before meals for days.14

Selection visit (V1):

The screening assessment will be performed in the days15 preceding the randomization visit (first day of treatment)

After receiving information about the trial and signing the informed consent form, an initial assessment will be performed. This will include:

- Clinical and neurological assessment: physical examination, blood pressure, electrocardiogram (ECG)
- Biological check-up: hematology, biochemistry, hepatitis B and C serology, HIV serology, urine analysis, urine pregnancy test (if you are a woman of childbearing age),
- Evaluation scales (spasticity, pain)
- Blood sampling for biomarkers and genetics (optional). These biomarkers can be substances such as proteins whose knowledge and evolution under treatment can help to understand the symptoms

If you meet all of the inclusion criteria and none of the non-inclusion criteria, you will be eligible and we will contact you by phone to inform you whether or not you are participating in the study.

Randomization visit (D1) (V2)

You will be summoned to the department on the morning of the visit. After verification of the inclusion and non-inclusion criteria, a clinical and neurological check-up identical to that of the selection visit as well as the biological check-up (hematology and biochemistry) will be performed. Two blood samples (before administration of the treatment and 2 hours after administration1^ère^ of the study treatment) will be taken to measure the amount of drug in your body. The samples will be taken through a catheter placed in a forearm vein beforehand. You will be asked to fill in evaluation scales (spasticity, pain) and quality of life questionnaires and you will be given the study treatment and the patient diaries (to evaluate the day-to-day state of your spasticity and to note the daily intake of the study treatment) before leaving the center.

Intermediate visit (D4) (V3)

You will come to the center without having taken the medication in order to take a blood sample before administration of the treatment and a second one 2 hours after administration which will be done in the center. Your blood pressure will be recorded, and the patient's diaries (spasticity and taking the treatment) will be checked.

Follow-up visit (D14) (V4)

You will be called to the department on the morning of the visit. A clinical, neurological and biological check-up, with sampling for biomarkers (optional) identical to that of the selection visit (except for serologies and pregnancy test) will be performed. You will come to the center without having taken the medication and two blood samples will be taken (before administration of the treatment and 2 hours after administration). You will have to fill in again the evaluation scales (spasticity, pain) and quality of life questionnaires and bring back the patient diaries (spasticity and treatment intake) duly filled in as well as the treatment under study.

End of study visit (D21) (V5)

During this last visit, your blood pressure will be taken and you will be asked to fill out the evaluation scales (spasticity, pain) and quality of life questionnaires again.

A collection of adverse events and concomitant treatments will be systematically performed throughout the study during these visits.

The total volume of blood collected will be 80 ml.

Patients who have participated in the first stage may, if they wish, participate in the second stage.

### o Patient participating in the step 2

Patients will be randomized to one of two treatment groups (30 patients per group) and will receive either Riluzole at the dose defined in step 1 or placebo. The treatment, in the form of 2 capsules per dose, will be administered twice daily before meals for 14 days.

Selection visit (V1):

The screening assessment will be performed in the days15 preceding the randomization visit (first day of treatment)

After receiving information about the trial and signing the informed consent form, an initial assessment will be performed. This will include:

- Clinical and neurological assessment: physical examination, blood pressure, electrocardiogram (ECG)
- Biological check-up: hematology, biochemistry, hepatitis B and C serology, HIV serology, urine analysis, urine pregnancy test (if you are a woman of childbearing age),
- Evaluation scales (spasticity, pain)
- Blood sampling for biomarkers and genetics (optional). These biomarkers can be substances such as proteins whose knowledge and evolution under treatment can help to understand the symptoms
- Delivery of the mictional diary (to be filled out on a day within 3 days before the next visit)

If you meet all of the inclusion criteria and none of the non-inclusion criteria, you will be eligible and we will contact you by phone to inform you whether or not you are participating in the study.

Randomization visit (D1) (V2)

You will be summoned to the department on the morning of the visit. After verification of the inclusion and non-inclusion criteria, a clinical and neurological check-up identical to that of the selection visit as well as the biological check-up (haematology and biochemistry) will be carried out as well as two electromyograms, the first one before administration of the treatment and the second one two hours after administration. Two blood samples (before and 2 hours after the treatment) will be taken to measure the amount of drug in your body. The samples will be taken through a catheter placed in a forearm vein beforehand. You will be asked to fill in evaluation scales and quality of life questionnaires and you will be given the treatment under study as well as the patient diaries (spasticity and treatment intake). You will be asked to bring back the voiding diary.

Intermediate visit (D4) (V3)

You will go to the center without having taken your medication in order to take a blood sample before administering the treatment and a second one 2 hours after administration which will be done in the center. A biological assessment (hematology and biochemistry) will be performed. Your blood pressure will be recorded, and patient schedules will be checked. At the end of the visit, you will be given a voiding diary which will be filled out during the day1 in the 3 days preceding the next visit.

Follow-up visit (D14) (V4)

You will be called to the department on the morning of the visit without having taken the medication. A clinical, neurological and biological check-up, with sampling for biomarkers (optional) identical to that of the selection visit (except for serologies and pregnancy test) will be carried out as well as two electromyograms, the first before administration of the treatment and the second two hours after administration. Two blood samples (before and 2 hours after treatment) will be taken. You will have to fill in again the different evaluation scales and quality of life questionnaires and bring back the patient diaries (spasticity and treatment intake) duly filled in as well as the treatment under study.

End of study visit (D21) (V5)

During this last visit, your blood pressure will be taken and you will be asked to fill out the various evaluation scales and quality of life questionnaires again.

A collection of adverse events and concomitant treatments will be systematically performed throughout the study during these visits.

If you are being treated with Baclofen, six blood samples will be taken at the same time as the Riluzole samples (two samples on D1, D4 and D14 before and 2 hours after taking the study treatment) to measure the amount of Baclofen in your body.

The total volume of blood collected will be 111 ml.

| Visits | V1 | V2 | V3 | V4 | V5 |
| --- | --- | --- | --- | --- | --- |
|  | Screening | Randomization | PK | Efficien cy  é | Follow Up |
|  | 2 weeks  before D1 | J 1 S0 | J 4 | J 14 S 2 | J21 |
| Signature of informed consent | X |  |  |  |  |
| Criteria Inclusion/non-inclusion | X | X |  |  |  |
| fax/email for inclusion | X |  |  |  |  |
| Medical and surgical  history | X |  |  |  |  |
| Demographics | X |  |  |  |  |
| Vital signs, clinical examination and neurological | X | X | X | X | X |
| Blood samples (biology) | X | X | X | X |  |
| Blood samples (biomarkers and genetics) | X |  |  | X** |  |
| Randomization |  | X |  |  |  |
| Voiding diary * | X | X | X | X |  |
| Digital scale 0-10 NRS | X | X |  | X | X |
| Modified Ashworth scale | X | X |  | X | X |
| Skills assessment SCIM * functionalities |  | X |  | X |  |
| Penn Spasm Scale |  | X |  | X |  |
| IOGC Scale |  | X |  | X |  |
| Scale of achievement of objectives |  | X |  | X |  |
| ECG | X |  |  | X |  |
| Samples PK |  | X | X | X |  |
| EMG * |  | X |  | X |  |
| DN4 | X |  |  |  |  |
| Questionnaire for the evaluation of  neuropathic pain | X | X |  | X | X |
| ISCIPDS Questionnaire | X | X |  | X | X |
| Visual Analog Scale for pain | X | X |  | X | X |
| Delivery and return of the treatment under study |  | X |  | X |  |
| Concomitant treatments | X | X |  | X | X |
| Collection of events unwanted | X | X | X | X | X |

| Compliance |  |  |  | X |  |
| --- | --- | --- | --- | --- | --- |
| Rehabilitation (method and frequency) | X |  |  | X |  |

| Handing in and reviewing agendas  patient (treatment and spasticity) |  | X | X | X |  |
| --- | --- | --- | --- | --- | --- |

*Only for patients participating in the stage 2

**Only prelabelled biomarkers at V4 POTENTIAL RISKS

The primary risks associated with participation in this research project are:

- Blood tests may cause mild pain at the time of the prick and a small hematoma afterwards.
- The most frequently reported main effects of Riluzole were asthenia, nausea, and abnormalities of liver function tests.
- The electromyogram (EMG) is a test performed by an experienced physician and used to identify motor and sensory disorders in the limbs, and assess muscle contraction or weakness, paralysis, and pain. An EMG uses tiny devices called electrodes to transmit or detect electrical signals. The electrode is a needle inserted directly into a muscle; it records electrical activity in that muscle. You may experience local pain where the needle is inserted, but this resolves spontaneously after a few hours.

### Genetic sampling and biomarker analysis:

Some of your blood samples may be stored and used, if you agree, for a period of 5 years (after the end of this study).

In this case, the genetic samples will be kept at the Tissue, DNA, Cells Biological Resource Center - Department of Medical Genetics of the Timone Hospital, in an anonymous way, for further studies in the same pathology. This laboratory is declared under the number DC 2008-428 to the Ministry of Higher Education and Research and is authorized to store human body parts under the number AC 2011- 1312

Blood samples for biomarker analysis will be stored at the CIC (AP HM ) declared under the number DC 2011-1369

You may withdraw your consent to use your samples for future research at any time and for any reason. In this case, your samples will be destroyed as soon as they are no longer needed for the main study. You will need to inform your study doctor of your decision. This will not affect your medical care in any way. Your study physician will then inform the Sponsor to destroy your samples as soon as they are no longer needed for the study.

If you do not want your samples to be kept, they will be destroyed at the end of the research.

### CONSTRAINTS OF THE STUDY

The constraints of the study are as follows:

- By participating in this study, you agree to make yourself available for visits
- You will be required to take all scheduled exams during each session, according to the procedures explained in this document
- During your participation in the study, taking certain medications may affect the procedures. You must tell the study doctor about any medications you are taking before you agree to participate. You will also need to tell the study doctor before you take any new medications during the study.
- You will need to be easily reachable in case of an emergency.
- Finally, you must follow the rules and instructions that will be given to you during the study so as not to compromise the smooth running of the study.
- You will not participate in another study during the study and until days15 after the end of the study (exclusion period)

You should be aware that ignoring prohibitions or omitting or concealing information about your health status or the conditions of your participation in the study may result in adverse health consequences.

If you fail to comply with the terms of the protocol of which you have been informed, your participation may be immediately suspended. The study may be stopped at any time by decision of the Investigator, the Sponsor (AP-HM) or the Health Authorities.

### EXPECTED BENEFITS

Patients in the active treatment group may have an improvement in spasticity depending on the dose received. However, we cannot guarantee that you will have a benefit. Your participation in this study provides you with a closer and more specific medical follow-up than in routine medical practice, particularly focused on the evaluation of disability and quality of life, which may lead to better management.

### YOUR RIGHTS AS A VOLUNTEER

Your participation in the study is free and completely voluntary. You can interrupt your participation at any time without any consequences. The medical procedures of this study will be in accordance with the Public Health Code (Title II of Book 1 on biomedical research). This information is available on the Legifrance website [(](http://www.legifrance.gouv.fr/)www.legifrance.gouv.fr).

In accordance with articles L.1123-6, L.1123-7, L.1123-8, L.1123-9 and L.1123-10 of the Public Health Code, this protocol was submitted to the South Mediterranean Committee for the Protection of Individuals 1, which issued a favourable opinion on May 31 2016

In accordance with current legislation, this study also received authorization from the health authority (Agence Nationale de Sécurité du Médicament) on July 102017

You certify that you are covered by a social security system, that you are not subject to a legal protection system, nor deprived of your liberty by judicial or administrative measures.

The sponsor of the study (AP-HM, Marseille13005) has taken out insurance for this study with SHAM whose contract number is 145166.

You will not be deprived of any legal rights by signing this informed consent.

Transportation costs related to the study will be covered by the sponsor, upon presentation of receipts and within reason.

### PRIVACY

All information about you obtained during the study will be treated as confidential. No information bearing your name will be provided to anyone except the investigating physician and the entire clinical team in charge of the study.

The Health Authorities and the Sponsor's representative may have access to your medical record, in accordance with confidentiality and legal requirements, to verify the accuracy of the data collected.

The persons in charge of the quality control of this study and duly mandated for this purpose by the promoter will have access to your individual data strictly necessary for this control; these persons are subject to professional secrecy (article L1121-3 of the Public Health Code).

Your data will be identified by a code (letters and/or numbers) that will be kept strictly confidential in the investigator's file at the site. You will not be individually identified in any report. Your data will be computerized and used anonymously in a final report on the results obtained.

In accordance with the provisions of the law relating to data processing, files and freedoms (Law No. 78-17 of January 6, 1978 amended by Law No. 2018-493 of June 20, 2018), and the General Data Protection Regulation (RGPD) No. 2016/679 which came into force on May25 you 2018have the right to access, of rectification or deletion of your personal data, or a limitation of the processing or a right to object to the transmission of these data, covered by professional secrecy, likely to be used in the context of this research and to be processed.

You can also access all your medical data directly or through a doctor of your choice (article L 1111-7 of the Public Health Code).

Personal data is kept for 15 years

In case of difficulty in connection with the management of the collected data, you can address a complaint to the data protection officer within the Assistance Publique - Hôpitaux de Marseille, by e- mail to [dpo@ap-hm.fr](mailto:dpo@ap-hm.fr) or to the CNIL at n°01-53-73-22-22

The data concerning you, without mentioning your name, will be archived by the Sponsor and may be transmitted outside the European Union in order to be submitted to Health Authorities. Please note the confidential nature of the information related to this research.

### DECISION TO PARTICIPATE IN THE STUDY

It is your choice whether or not to participate in this study. You can refuse to participate and, even if you agree, you can withdraw from the study at any time without having to give reasons and without penalty.

Unless you notify us otherwise, if you withdraw your consent during the course of the study, the data collected during your participation up to the date of withdrawal will be kept for analysis. If new information that could affect your decision to participate becomes available during the course of the study, it will be communicated to you and to the investigating physician in charge of the study, and you will be asked to provide new consent.

The investigating physician in charge of the study and the Sponsor may decide at any time to stop the study without your consent if they feel that such a decision is justified.

If you agree to participate in this study, you will not be required to participate in any other study simultaneously.

A patient card mentioning your participation in the study will be given to you at your first visit, with the contact information of the investigating physician to be contacted in case of emergency. This card must be kept with you for the duration of the study and will be presented to any doctor you may consult during this period.

### RESULTS OF THE STUDY

You have the right to be informed of the overall results of this study. You can obtain them, when they are available, from the investigating physician.

Whatever your decision regarding participation in this research, we thank you for your time and attention.

# Consent Form

**"Study of Riluzole in the treatment of spasticity after chronic traumatic spinal cord injury: a double-blind, randomized, placebo- controlled, multicenter, adaptive trial in a rare disease: RILUSCI"**

**INVESTIGATOR'S SECTION**

Name of investigator: ..............................................................................................

Investigator's first name: ..........................................................................................

Business address:..............................................................................................

Phone number: .................................................................................................

I confirm that I have given the subject a detailed explanation of this biomedical study and have verified that he or she has health coverage. I provided the subject with the information document and answered all questions regarding the study.

# SECTION RESERVED FOR THE SUBJECT

Subject name: .............................................................................................................

Subject's first name: ....................................................................................................... ..

Subject's full address: ........................................................................................

....................................................................................................

....................................................................................................

# I have received, read and understood the information document regarding the study entitled

**"Study of Riluzole in the treatment of spasticity after chronic traumatic spinal cord injury: a double-blind, randomized, placebo-controlled, multicenter, adaptive trial in a rare disease: RILUSCI"**

whose promoter is the AP-HM (13005 Marseille). I have also received sufficient explanations about the biomedical study and its implications, about the potential inconveniences and risks to my health and well-being, as well as about my rights and obligations. I have had the opportunity to ask questions before making my decision and I have had sufficient time to reflect between the time I received the information and the time I gave my consent. I will have the opportunity to ask the investigator further questions at any time. I understand that my consent does not relieve the study sponsor or the investigators of their responsibilities.

I know that this biomedical study was approved by the Comité de Protection des Personnes Sud Méditerranée 1 on May 31, 2016 and authorized by the ANSM on July 10, 2017 ].

I am also aware that, as required by law, the study sponsor has purchased an insurance policy

from SHAM Policy No. 145166.

I understand that the decision to participate in this study is mine alone and that I have the right to change my mind during the course of the study without having to explain myself. If I change my mind, I will have to inform the study doctor. If new information is obtained during the course of the study that may affect my decision to participate, I will be informed. I also understand and agree that the study may require access to certain important data from my medical records and that, in accordance with local law, data collected during the study may be subject to review by judicial and health authorities and by representatives of the sponsor. I am aware that

that it will not be possible to identify me from the information in my medical record and that all data will be treated in strict confidence.

Data collected during the study will be digitized and managed according to local requirements. I agree that my anonymized data may be archived and transmitted outside the European Union for submission to health authorities.

I agree to the computerized processing of my personal data in accordance with the provisions of Law 78-17 of January 6, 1978 relating to data processing, files and freedoms and subsequent laws including Law No. 2018-493 of June 20, 2018 relating to the protection of personal data and in accordance with the General Data Protection Regulation No. 2016/679 which entered into force on May 25, 2018)),

I will have the right to be informed of the overall results of this study. I will be able to obtain them, when available, from the investigating physician.

I have received two originals of this document, one of which I will keep, and I have been informed that the other will be kept by the study physician.

# On this basis, I freely and willingly agree to participate in this study. I certify on my honor that I am not under court protection, guardianship or curatorship.

The doctor suggested that I participate in

- the stage 1
- the stage 2

Location: ..................................................................

Date: ..................................................................

***PATIENT***

Full name: .....................................................................................

Signature:

*(Write below: "Read, understood and approved")*

## INVESTOR

Full name: .....................................................................................

Signature:

# Consent form for optional samples and analyses

**Study of Riluzole in the treatment of spasticity after chronic traumatic spinal cord injury: a double-blind, randomized, placebo-controlled, multicenter, adaptive trial in a rare disease: RILUSCI**

Sponsor: AP-HM (Assistance Publique des Hôpitaux de Marseille)

Clinical Research and Innovation Department - rue 80Brochier - Marseille 13354Cedex 5 Coordinating Investigator: Pr Viton

I, the undersigned, Mrs., Mr, (name, first name) Residing at the following address :

Recognize:

1. To have freely accepted my participation in the biomedical research entitled **Study of Riluzole in the treatment of spasticity after chronic traumatic spinal cord injury: a double-blind, randomized, placebo-controlled, multicenter, adaptive trial in a rare disease: RILUSCI"**, without this releasing the organizers of the research from their responsibilities
2. To have understood that a genetic marker study will be carried out as part of this study and to have received the information defined in article R 1131 of the decree n° 2008-321 of April 04, 2008 in compliance with the provisions of article 35 of the decree n° 95-1000 of September 6, 1995 concerning the code of ethics, and to have consented to the taking of the genetic analysis(s) mentioned above.
3. To have understood that I have a period of reflection between the time I was given the information and the time I sign this document,
4. I have been informed that I may withdraw my agreement to participate at any time, without justification and without affecting my relationship with the health care personnel or my care,
5. I have been informed that I retain all my rights guaranteed by law (Law n° 2012-300 of March5 relating2012 to research involving the human person (known as the Jardé Law) / Public Health Code; title II of book one relating to research involving the human person),
6. Have been informed that this research received a favorable opinion from the Comité de Protection des Personnes Sud Mediterranée 1 on May 31, 2016 and that the Competent Authority, the Agence Nationale de Sécurité des Médicaments et des Produits de Santé (ANSM) authorized this study in July10 2017
7. To have been informed of the objective, progress, benefits and risks of this study, and to have been informed that this study will be conducted according to the Good Clinical Practices defined in the Official Bulletin published by the Ministry of Social Affairs and Employment,
8. To have been able to ask all the questions I wanted and to have received adapted answers that I clearly understood, and to have noted that I could complete this information throughout the study with my doctor or with Dr. , coordinating investigator, at

the TIMONE Hospital, Marseille, department of ................., tel: ..........

1. Have been informed that the Sponsor of this study, which is represented by the Assistance Publique des Hôpitaux de Marseille (80 rue Brochier - Marseille 13354cedex 5), has taken out a "Civil Liability" insurance policy in accordance with the law in force with the company SHAM (policy n°145166),
2. I have been informed that the anonymous use of my data, collected in the framework of this research, will be subject to computerized processing and that the presentation of the results of the study will not allow my direct or indirect identification
3. Have been informed that these data may be consulted by persons, who are all bound by professional secrecy, such as the investigators and their collaborators, the sponsor and the persons mandated by him/her and the persons mandated by the Competent Authorities (administrative, health and judicial),
4. Have been informed that I may if I wish access this data, verify it and request modifications if necessary, in accordance with the law in force (guaranteed by the articles and39 of40 the law n°78-17 of January 6relating1978 to data processing, files and freedoms and subsequent laws including the law n°2018-493 of June 20, 2018 relating to the protection of personal data and in accordance with the General Data Protection Regulation n°2016/679 which entered into force on May 25, 2018)),
5. I have noted that any new information that arises during the course of the study, which could affect my participation, will be communicated to me as soon as possible,
6. Understand that the sponsor or investigator may decide at any time to discontinue the study,
7. I have been informed that the overall results of the study may be communicated to me in accordance with article L 1122-1 of the Public Health Code,
8. Have understood that if I agree to participate in this research, I must sign this document.

# I agree to participate in this study, under the conditions specified above And

**Regarding the samples (for biomarkers) taken in this study:**

# I authorize

- **I do not authorize**

The conservation and future use of these samples and associated data, in the context of another research study on this pathology, knowing that these data will be used in an anonymous manner**.**

# And

**I indicate if I agree to the genetic sample**

# Yes, I accept

- **No, I do not accept**

Done at : On: Last Name, First Name (Patient): Signature (Patient) : (preceded by the words **"Read, understood and approved"**)

**Signature of the investigating physician**, who certifies that: he/she has given the patient the necessary information concerning this study, has allowed the patient the time necessary to read this information leaflet and consent form, and has answered all these questions.

The : Physician's name: Signature:

# Done in duplicate.

(one for the investigator, one for the patient)
